# Mechanism of Silylation of Vinyl Arenes by Hydrodisiloxanes Driven by Stoichiometric Amounts of Sodium Triethylborohydride—A Combined DFT and Experimental Study

**DOI:** 10.3390/ijms24054924

**Published:** 2023-03-03

**Authors:** Mateusz Nowicki, Maciej Zaranek, Magdalena Grzelak, Piotr Pawluć, Marcin Hoffmann

**Affiliations:** 1 Faculty of Chemistry, Adam Mickiewicz University, Uniwersytetu Poznańskiego 8, 61-614 Poznań, Poland; 2 Centre for Advanced Technologies, Adam Mickiewicz University, Uniwersytetu Poznańskiego 10, 61-614 Poznań, Poland

**Keywords:** DFT, M06-2X, silylation, dimethylsilane surrogate, triethylborohydride, disiloxanes

## Abstract

The reactions of vinyl arenes with hydrodisiloxanes in the presence of sodium triethylborohydride were studied using experimental and computational methods. The expected hydrosilylation products were not detected because triethylborohydrides did not exhibit the catalytic activity observed in previous studies; instead, the product of formal silylation with dimethylsilane was identified, and triethylborohydride was consumed in stoichiometric amounts. In this article, the mechanism of the reaction is described in detail, with due consideration given to the conformational freedom of important intermediates and the two-dimensional curvature of the potential energy hypersurface cross sections. A simple way to reestablish the catalytic character of the transformation was identified and explained with reference to its mechanism. The reaction presented here is an example of the application of a simple transition-metal-free catalyst in the synthesis of silylation products, with flammable gaseous reagents replaced by a more convenient silane surrogate.

## 1. Introduction

Interest in hydroelementation reactions, especially hydroboration and hydrosilylation and, to a lesser extent, hydrogermylation and hydrostannylation, has persisted among chemistry researchers for several decades. This has resulted in the literature characterization of numerous catalysts for these reactions, both already-known ones used in other reactions and newly developed ones. For hydrosilylation, the relatively simple catalysts based on peroxides [1] or platinum and other transition metals [2] originally used in this reaction were gradually joined by their more sophisticated successors, such as multinuclear clusters and pincer complexes, rare earth compounds, and finally catalysts that contain only earth-abundant elements of the s and p blocks. As their mechanisms of action can be very diverse and their chemo-, regio-, and stereoselectivity can be quite surprising, computational methods based on quantum chemistry are emerging as an extremely useful tool in research on such catalytic systems. Because of the rapid increase in the computational capabilities of modern computers and the vibrant development of the methods themselves, scientists are utilizing them to take a closer look at compounds and processes under investigation.

The extensive but perhaps non-exhaustive register of hydrosilylation reactions studied with the use of the quantum-chemical approach includes catalysts based on platinum [1], palladium [2,3], rhenium [4,5], ruthenium [6,7,8,9], tungsten [10], molybdenum [11,12,13], nickel [2], cobalt [14,15], iron [16], manganese [17], samarium [18,19], lanthanum [19], yttrium [19], zinc [20], and boron [21,22]. With regard to the latter element, in recent years, we described combined experimental and theoretical studies in which DFT methods were used to explain the hydrosilylation and hydrogermylation reactions. The application of these methods allowed us first to propose a mechanism for the selective Markovnikov hydrosilylation catalyzed by sodium triethylborohydride (and, consequently, to explain such regioselectivity of this catalytic system and its activity towards various hydrosilanes) [22]. Later, the use of DFT modeling led us to find the explanation for the opposite regioselectivity of hydrogermylation under analogous experimental conditions [23]. In this article, we report on a reaction of hydrodisiloxanes with alkenes in the presence of NaHBEt_3_. The experimental result of the reaction under investigation turned out to be a product of formal hydrosilylation with dimethylsilane, Me_2_SiH_2_, for which we initially could not provide any explanation. However, now having practical experience in the DFT modelling of similar systems, we decided to carry out a thorough quantum-chemical study to elucidate the mechanism of the observed transformation. Here, we explain, with reference to the electronic and geometric structures of intermediates and transition states, why, in this reaction, the relatively strong Si-O bond is broken instead of the weaker Si-H one. Reactions leading to formal hydrosilylation products with dimethylsilane and other volatile and/or pyrophoric silanes have gained attention mainly as implementations of transfer hydrosilylation using the so-called silane surrogates studied most comprehensively by Oestreich [23,24,25,26,27,28], with a more limited contribution from others [29,30,31,32]. The last of these works [32] is of particular relevance, since its authors used the same starting materials as ours.

## 2. Results and Discussion

### 2.1. Experimental Results

**Scheme 1:**
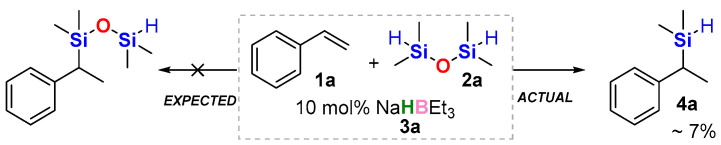
Expected vs. actual product of the initial experiment.

Our experimental investigation started with an observation made during previous studies on the hydrosilylation of vinyl arenes. When 1,1,3,3-tetramethyldisiloxane (TMDSO) was used as a hydrosilane, the only silylated product that could be detected was a small amount of dimethyl(1-phenylethyl)silane **4a** instead of the expected hydrosilylation product of hydrosilylation (Scheme 1).

Initially, this first occurrence of a new process did not seem prospective; nevertheless, we decided to look closer at this reaction. From the very beginning, it was conceived that the amount of sodium triethylborohydride relative to that of styrene might be responsible for the very low yield of the product. It turned out that conversions of styrene greater than 80% were observed only when stoichiometric amounts of NaHBEt_3_ were used, and nearly all styrene was consumed only when this amount was superstoichiometric (1.5 equiv.) with an excess of TMDSO (2.0 equiv.). As a consequence, the possibility of any catalytic action was quickly ruled out. Such a high amount of the needed siloxane could result from its boiling point (70–71 °C) being lower than the reaction temperature (initially 100 °C, then 80 °C), and thus its significant amount could be present in the vapor over the reaction solution. Under all conditions, if conversion of styrene was detected, it only led to product **3a**. Unfortunately, we were unable to isolate this product because of its volatility; therefore, we decided to switch to 4-*tert*-butylstyrene for the next experiments.

The reaction temperature of 100 °C initially used could be lowered to 80 °C without a significant loss of styrene conversion after 20 h (97 vs. 94%), and the latter temperature was used in the subsequent experiments. Table 1 summarizes the selection of the reaction conditions and the first mechanistic experiments. It is worth noting that some of these were conducted in parallel to the computations because of questions arising from the results of the latter and are included in Table 1 for an easier general overview.

A further decrease in the temperature to 70 °C (entry 8) led to a decrease in **4b** yield below the acceptable level. Potassium and lithium triethylborohydrides were also examined, and their application to the reaction system resulted in a general decrease in selectivity toward the desired product (entries 9 and 10). As the computations unfolded, it was pointed out that BEt_3_ could be used in catalytic amounts. Entries 11–14 show that indeed, the use of NaH and BEt_3_ separately, even in superstoichiometric amounts, did not lead to the formation of **4b**, and combining them in a 15:1 ratio resulted in a catalytic reaction, although very slow. Another important aspect of the reaction system is its applicability to other similar disiloxanes. The use of 1,1,1,3,3-pentamethyldisiloxane (PMDSO) **2b** instead of TMDSO **2a** resulted in only an insignificant change in the conversion of **1b**, while hexamethyldisiloxane (HMDSO) **2c** did not produce any silylation products. The origin of these results is explained further in Section 2.2.

**Scheme 2:**
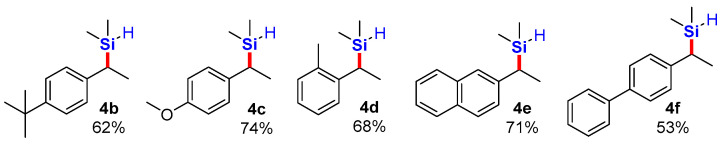
Isolated products of silylation using TMDSO as a dimethylsilane surrogate in the presence of stoichiometric sodium triethylborohydride.

We applied the conditions as in entry 5 to a total of five vinyl arenes and isolated the corresponding products, as shown in Scheme 2.

### 2.2. Computational Results

**Scheme 3:**
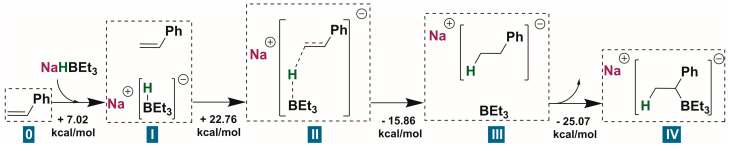
Initial steps in the reactions investigated (described in prior research).

In order to elucidate the mechanism of the reaction under investigation, we decided to employ quantum-chemical methods as in our previous studies on a similar reactions [22,33]. As before, we used BMe_3_ to represent BEt_3_; this practice allows significantly faster calculations with a negligible loss of precision. Errors resulting from such a simplification overlap in the calculations for subsequent structures and eventually cancel out, as ultimately it is the relative energy difference that is important, and it is from this difference that conclusions about the mechanism are drawn. The mode of double-bond activation and the formation of a secondary carbanion (Scheme 3), which promotes the formation of a Markovnikov product, was already elucidated and is not discussed here in detail; only the paths leading to Markovnikov products are presented hereafter. Please see Supplementary Materials for detailed computational results.

#### 2.2.1. Reaction Energy Profiles

**Figure 1:**
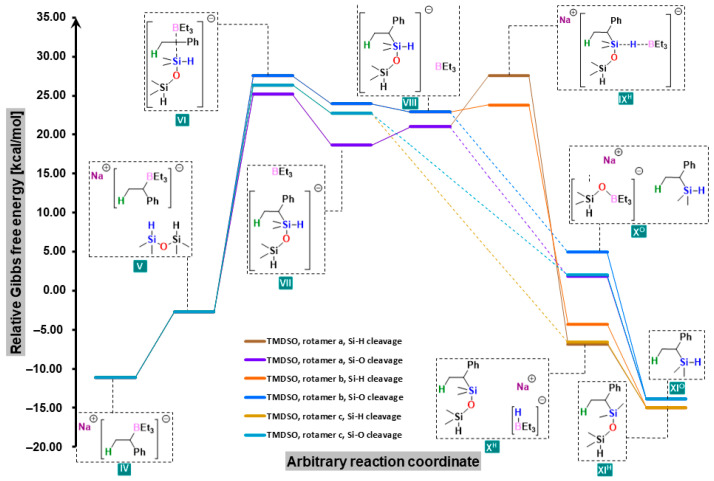
Free-energy profiles for two possible pathways of the reaction of styrene with TMDSO in the presence of sodium trialkylborohydride.

**Figure 2:**
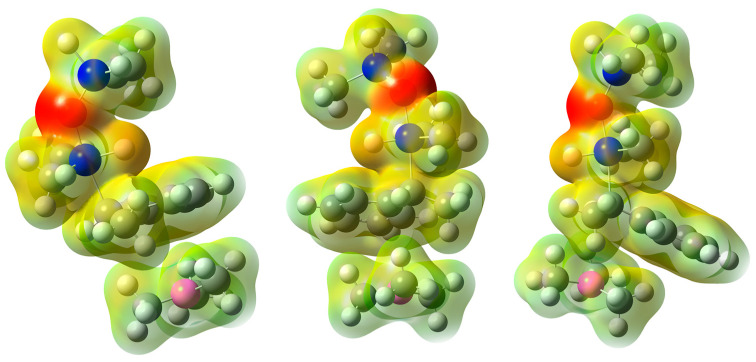
Electron density (mapped by electrostatic potential at 0.01 e^-^·a_0_^−3^ isovalue) for **VIIa**, **VIIb,** and **VIIc** structures (left to right) in the reaction with TMDSO (blue for silicon, magenta for boron, red for oxygen). BMe_3_ was used for computations in place of BEt_3_. Note how the silicon-bonded hydrogen atom is sterically hindered in the lowest-energy **a** and **b** conformers.

Our primary focus was to find an explanation for why regular hydrosilylation was not observed; instead, the breaking of a strong Si-O bond was preferred (Figure 1). Carbanion **IV** approached a TMDSO molecule (**V**) and attacked it (**VI**) to form a pentacoordinate silicanion **VII** in a manner similar to that found in the reaction with phenylsilane. This entity, when approached by a trialkylborane molecule from another side (**VIII**), attacked the boron atom, one of its bonds was broken, and a final product of the reaction was obtained. The attack could be from either the hydrogen atom (**IX^H^**, with Si-H cleavage leading to **X^H^** and final product **XI^H^**) or the oxygen atom (with Si-O cleavage leading to **X^O^** and final product **XI^O^**). Indeed, for the reaction of styrene with TMDSO, the abstraction of the -OSiMe_2_H moiety led to a product which had an approximately 14 kcal/mol lower free energy than the substrates, though slightly higher than that of the product formed after Si-H bond cleavage. However, the reaction led to dimethyl(1-phenylethyl)silane due to lower transition states and intermediates. The intermediates **VII, VIII, IX and X** can exist as three stable rotamers generated by rotation around the C-Si bond (Figure 2). Each of them has rotamer **a** in which a silicon-bonded hydrogen atom is anti-periplanar to a carbon-bonded hydrogen, rotamer **b** in which the former is anti-periplanar to the methyl group, and rotamer **c** in which it is anti-periplanar to phenyl. They are arranged in three distinct pathways differing in energies and even, as it turned out, in the number of stationary points. Although the Gibbs free energy differences between them do not exceed 6 kcal/mol, they turned out to be of great significance. In the case of Si-H bond cleavage, it can proceed without free energy barrier (no identifiable transition state) only from the least favored conformation of **VII** (conformation **c**); in the other two conformations, the transition state **IX** is present between **VIII** and **X**. For the lowest-energy conformer **a**, the additional free energy barrier **IX^H^a** is almost 7 kcal/mol, which is not without significance for already high-energy **VIII** intermediates. When it comes to the breaking of the Si-O bond, the final step is barrierless regardless of the rotamer. These factors not only explained why the Si-O bond was cleaved instead of Si-H and produced dimethyl(1-phenylethyl)silane as the main product, but also elucidated why an equimolar amount of NaHBEt_3_ had to be used in the reaction.

**Figure 3:**
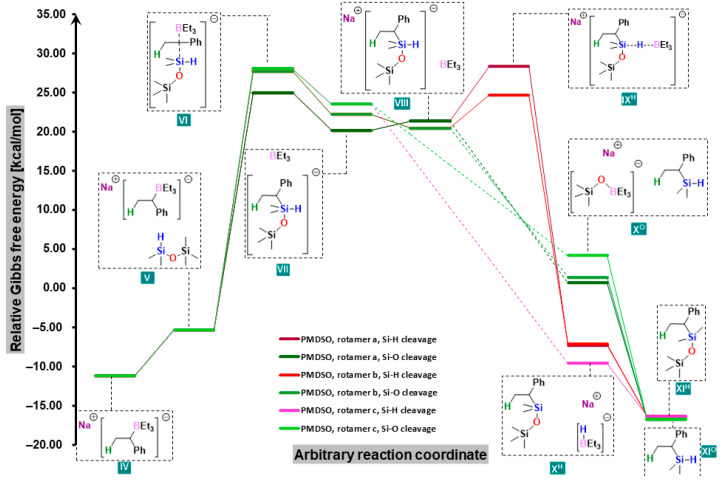
Free-energy profiles for two possible pathways of the reaction of styrene with PMDSO in the presence of sodium trialkylborohydride.

**Figure 4:**
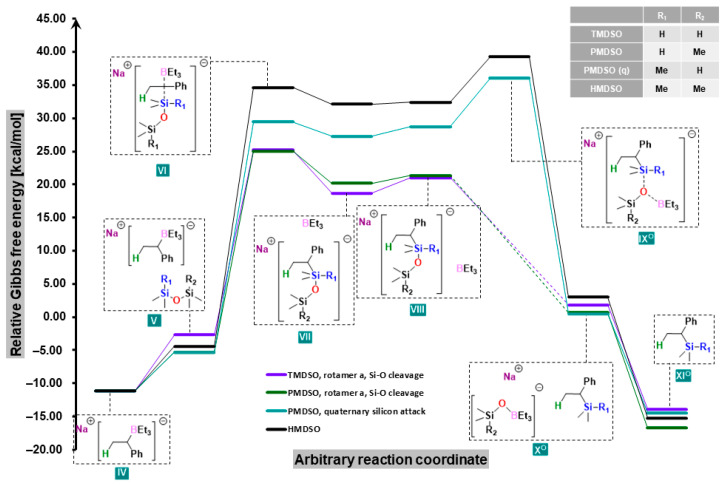
Free-energy profiles for the reactions of styrene with disiloxanes (with Si-O cleavage) in the presence of sodium trialkylborohydride.

Similar relationships are evident for PMDSO when it bonded to the carbanion with a less substituted silicon atom (Figure 3). If, on the other hand, it attached to a more substituted atom (which is energetically unfavorable by ca. 4 kcal/mol), or if HMDSO was used, all VI–IX structures had energies 4–14 kcal/mol higher than those of TMDSO (Figure 4).

#### 2.2.2. O-Si-H 2D Scan

**Figure 5:**
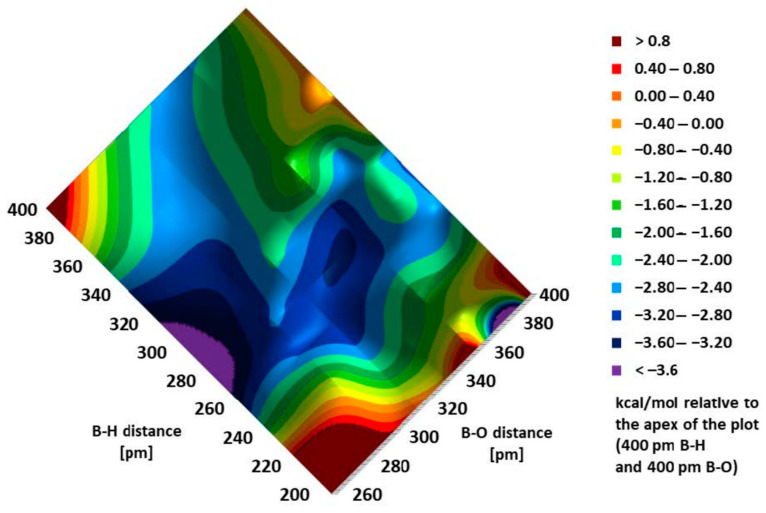
Energy map obtained by a relaxed potential energy hypersurface scan of a quasi-**IX** structure, which determined the formation of borasiloxyl salt.

The oxygen and hydrogen atoms bonded to the silicon atom discussed above, whose interactions with the boron atom determined the further course of the reaction, are relatively close to each other (230 pm). Therefore, we considered that the results of the potential energy hypersurface scans presented above, where only one reaction coordinate (B-O or B-H distance) was controlled, should be verified with more detailed calculations in case the specifics of the optimization algorithm led us to hasty conclusions regarding the reaction pathways. A scan was launched that controlled both of these interatomic distances at the same time to produce a more illustrative three-dimensional graph (Figure 5). Only conformer **c**, in which boron and oxygen were both approached from the same side and appeared to bind to boron with a negligible energy barrier, was selected for this computation.

Three high-energy (red) areas can be distinguished in this plot: left, right, and bottom corners, where one or both atoms (H and O) are approached at an unfavorable angle; the top-central (green and light blue) area is characterized by a low energy gradient; two low-energy areas on the bottom-right and bottom-left edges (dark blue and violet) lead to two possible products. Without recourse to differential calculus, it is easy to see that the formation of the B-O bond occurred spontaneously, without overcoming any energy barrier. Meanwhile, the barrier needed to form a B-H bond, although small (ca. 3 kcal/mol), was on a narrow pathway with a steep energy gradient.

#### 2.2.3. Trialkylborohydride Regeneration

**Figure 6:**
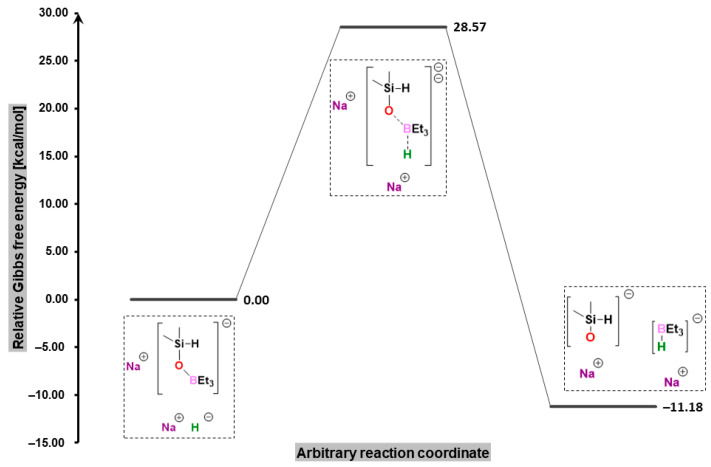
Free-energy profile for the reaction of the borasiloxyl salt with sodium hydride, leading to sodium trialkylborohydride regeneration.

Finally, in order to shed light on the restoration of the catalytic action of triethylborane in the presence of sodium hydride, we investigated the interaction of the latter with the borasiloxyl salt produced in the main reaction. As our findings suggested, the reaction took place in accordance with an S_N_2 mechanism in which the cleavage of the B-O bond and the simultaneous formation of the B-H bond (Figure 6) required overpassing a Gibbs free energy barrier of 28.6 kcal/mol, regenerated sodium trialkylborohydride, produced sodium dimethylsilanolate as the final product, which decreased the overall Gibbs free energy of the system by 11.2 kcal/mol.

#### 2.2.4. Substituted Styrenes

Later on, it was suggested that we carried out additional studies in which 4-methoxy and 4-trifluoromethyl substituents were attached to the aromatic ring in order to examine the influence of their electronic properties on the reaction. The energies obtained for the previously determined reaction pathway suggested a slightly easier activation of the C=C bond in 4-methoxystyrene and a much lower barrier in 4-(trifluoromethyl)styrene (Table 2). In the case of the latter compound, this difference seemed easy to understand and was attributed to a better distribution of the negative charge at transition state **II** and subsequent intermediates due to the influence of the -CF_3_ group. The calculations seemed to suggest a simple activation of the C=C bond, which could not be confirmed by experimental work as the reaction led to completely different products, resulting from the reduction of the CF_3_ group accompanied by precipitation of sodium fluoride. Less obvious at first sight was the influence of the -OMe group. Indeed, its resonance electron-donating properties gave the β carbon atom a slightly higher partial negative charge than in styrene (−0.224 vs. −0.177 APT charge at **I**). At transition state **II**, however, it was the inductive electron-withdrawing effect of the oxygen that tipped the balance; the energy difference between **I** and **II** was thus smaller than for styrene, since the nearby boron atom allowed the carbanion to stabilize immediately by forming **IV**, which had almost the same relative energy as for styrene. This was reflected in the experiment in which the product of silylation of 4-methoxystyrene formed with essentially the same efficiency as that measured when starting from bare styrene. It is therefore safe to claim that the presence of the electron-donating methoxy group did not adversely affect the reaction yield.

## 3. Materials and Methods

### 3.1. Experimental Methods

#### 3.1.1. General Considerations

All experiments were performed under inert atmosphere using standard Schlenk techniques. Styrene (Merck/Sigma-Aldrich, St. Louis, MO, USA) was vacuum-distilled over calcium hydride prior to use; other vinyl arenes (Merck/Sigma-Aldrich) were used as received. The compound 1,1,3,3-tetramethyldisiloxane, TMDSO (ABCR), was stored over dried 4A molecular sieves. Sodium, lithium, and potassium triethylborohydrides (Merck/Sigma-Aldrich) were purchased as 1 mol/L solutions and used as received.

Gas chromatography was performed on a Bruker Scion 436-GC system with a TCD detector. GC-MS analyses were performed on a Bruker Scion 436-GC system with a Scion SQ-MS mass spectrometry detector. NMR analyses were performed on a Bruker Fourier 300 MHz or Bruker Avance III HD 400 MHz spectrometer and referenced to the solvent residual peak.

#### 3.1.2. General Experimental Procedure of Silylation Using TMDSO as a Me_2_SiH_2_ Surrogate

In an oven-dried Schlenk bomb flask filled with argon, 1.0 mmol of vinyl arene and 2.0 mmol (267 mg, 353 µL) of 1,1,3,3-tetramehyldisiloxane were placed, followed by stirring with a magnetic bar. Next, 100 µL of decane was added along with 1.5 mmol (1.5 mL of 1 M solution) of sodium triethylborohydride. No additional solvent was used. A reference GC sample was drawn, and the reaction vessel was sealed with a PTFE plug and placed in an oil bath preheated to 80 °C. The reaction mixture was stirred at this temperature overnight for 20 h. Next, after the vessel was cooled to room temperature, another GC sample was taken. Once a high conversion of the vinyl arene was determined, the reaction mixture containing the desired product was subjected to purification. First, toluene and the unreacted excess of TMDSO were removed under vacuum. Caution should be exercised, as light silylated products may be volatile. The mixture of product and siloxane byproducts was separated from the residues of borohydride by extraction with hexane. The solvent was evaporated, and the residue was extracted once again with methanol/ethyl acetate (4:1 *v*/*v*) to separate the product from most of the siloxanes insoluble in methanol. Upon evaporation of the solvent, the residue was purified by column chromatography using hexane/ethyl acetate (95:5 *v*/*v*) as the eluent.

### 3.2. Identification of the Products

#### 3.2.1. (1-(4-Tert-Butylphenyl)Ethyl)Dimethylsilane, **4b**; Colorless Viscous Oil

^1^H NMR (300 MHz, CDCl_3_), δ: 7.25–7.30 (m, 2H), 7.00–7.05 (m, 2H), 3.79–3.86 (m, 1H), 2.23 (dq, *J* = 6.92, 2.51 Hz, 1H), 1.36 (d, *J* = 7.47 Hz, 3H), 1.29 (s, 9H), 0.01 (dd, *J* = 12.07, 3.52 Hz, 6H); ^13^C NMR (75 MHz, CDCl_3_), δ: 147.1, 142.1, 126.4, 125.0, 34.1, 27.0, 15.3, −6.0

Conforms to the literature [32].

#### 3.2.2. (1-(4-Methoxyphenyl)Ethyl)Dimethylsilane, **4c**; Off-White Viscous Oil

^1^H NMR (300 MHz, CDCl_3_), δ: 6.98–7.05 (m, 2H), 6.80–6.87 (m, 2H), 7.40–7.48 (m, 2H), 3.81–3.86 (m, 1H), 3.80 (s, 3H) 2.22 (qd, *J* = 7.55, 2.86 Hz, 1H), 0.02 (dd, *J* = 12.73, 3.64 Hz, 6H); ^13^C NMR (75 MHz, CDCl_3_), δ: 156.9, 137.2, 127.7, 113.7, 55.2, 26.6, 15.7, −5.9, −6.0

Conforms to the literature [32].

#### 3.2.3. Dimethyl(1-(2-Methylphenyl)Ethyl)Silane, **4d**; Colorless Viscous Oil

^1^H NMR (300 MHz, CDCl_3_), δ: 7.05–7.20 (m, 3H), 6.97–7.03 (m, 1H), 3.84 (pd, *J* = 3.63, 2.73 Hz, 1H), 2.34 (s, 3H), 2.25 (qd, *J* = 7.51, 2.82 Hz, 1H), 1.40 (d, *J* = 7.49 Hz, 3H), 0.03 (dd, *J* = 12.21, 3.62 Hz, 6H); ^13^C NMR (75 MHz, CDCl_3_), δ: 144.6, 136.8, 127.2, 126.9, 124.5, 123.2, 26.7, 20.7, 14.5, −6.8

Conforms to the literature [32].

#### 3.2.4. Dimethyl(1-(Naphthalen-2-yl)Ethyl)Silane, **4e**; Colorless Viscous Oil

^1^H NMR (300 MHz, CDCl_3_), δ: 7.58–7.73 (m, 3H), 7.40 (s, 1H) 7.23–7.37 (m, 2H), 7.09–7.20 (m, 1H) 3.77–3.86 (m, 1H), 2.35 (qd, *J* = 7.39, 2.52 Hz, 1H), 1.40 (d, *J* = 7.45 Hz, 3H), −0.06 (dd, *J* = 12.26, 3.60 Hz, 6H); ^13^C NMR (75 MHz, CDCl_3_), δ: 142.3, 132.8, 130.4, 126.6, 126.5, 126.3, 125.9, 124.8, 123.6, 123.1, 27.0, 14.4, −6.9

Conforms to the literature [32].

#### 3.2.5. (1-([1,1′-Biphenyl]-4-yl)Ethyl)Dimethylsilane, **4f**; Off-White Solid

^1^H NMR (300 MHz, CDCl_3_), δ: 7.64–7.58 (m, 2H), 7.50–7.55 (m, 2H), 7.40–7.48 (m, 2H) 7.29–7.37 (m, 1H), 7.14–7.21 (m, 2H), 3.90 (pd, *J* = 3.65, 2.70 Hz, 1H), 2.35 (qd, *J* = 7.48, 2.78 Hz, 1H) 1.46 (d, *J* = 7.50 Hz, 3H), 0.07 (dd, *J* = 11.01, 3.61 Hz, 6H); ^13^C NMR (75 MHz, CDCl_3_), δ: 144.5, 141.0, 137.2, 128.6, 127.1, 126.9, 126.7, 126.8, 27.4, 15.2, −6.0

Conforms to the literature [32].

### 3.3. Computational Methods

All computational data throughout this study were obtained with the use of M06-2X exchange-correlation functional [34], which is recognized for a credible description of systems composed of main group elements [35,36], including organometallics [22,37]. The 6-31+G(d) basis set [38,39,40,41,42,43,44] was used during geometry optimizations in order to balance the accuracy of the results and a feasible consumption of disc space resources and computational time. The Gaussian 16 program package [45] was used for all quantum-chemical computations within the study. Based on our previous research on trialkylborohydride-catalyzed reactions, [22,33] we replaced sodium triethylborohydride with sodium trimethylborohydride to accelerate and simplify the calculations. The initial structures of all molecules were generated on the basis of the average values of bond lengths and valence angles, [38], followed by a complete optimization of the geometry toward potential energy minima. The approximate geometries of the respective transition states were identified by relaxed potential energy scans as functions of the distances between atoms expected to form bonds, followed by full optimization via the synchronous transit-guided quasi-Newton method (QST3) [46]. All successfully identified transition states were confirmed to correspond to the first-order saddle points by calculating force constants and the resulting vibrational modes, which involved exactly one imaginary frequency corresponding to stretching the bonds being broken and formed (freq calculations), and validated by pseudo-IRC calculations to verify whether a given TS indeed connected the respective potential energy minima. [40] The data for Figure 5 were obtained via a relaxed potential energy scan with two variables (B-O and B-H interatomic distances) controlled in the range of 200–400 pm with a 20 pm step, followed by bicubic interpolation [41] with a 1 pm step to obtain a smooth curvature. All final energies and charge distributions were calculated using the 6-31++G(d,p) basis set, [38,39,40,41,42,43,44], which provides a more reliable description of hydride anions and their transfer, [39] at a temperature T = 373.15 K and with solvent correction (using toluene to reproduce the experimental conditions) within the usual approximation, that is, the polarizable continuum model (PCM) [47,48,49].

## 4. Conclusions

Although the hydrosilylation reaction would lead to thermodynamically more stable products, because of the relatively high-energy barriers, the actual reaction results in the formation of a borasiloxyl salt and a hydrosilane. Figure 2 clearly illustrates how the differences in energy between different rotamers of **VII** and, consequently, different transition states of IX correspond to the steric hindrance of the hydridic hydrogen atom, which is most evident for the lowest-energy conformer **VIIa**. Even in **VIIc**, where hydrogen appears to be quite exposed, the proximity of the oxygen atom, with two lone electron pairs and much higher electron density, prohibits the formation of the B-H bond and provides a clear explanation for the unexpected outcome of the reaction. The study also demonstrated the attention that scientists conducting quantum-mechanical calculations to determine reaction mechanism should pay to the possibility that a given structure may exist in the form of different rotamers, differing slightly in energy but leading to decisive energy differences between the transition states that will follow. It is also worth noting how a potential-energy hypersurface scan with two independent variables, although much more time- and resource-consuming, provides an incomparably more complete picture of the interactions and mechanism than a one-dimensional scan. This is especially important when the transition states are located on a very flat part of the PES, making them very difficult or impossible to identify, and even more so when the reaction path splits smoothly into two, each leading to completely different products [50,51].

## Figures and Tables

**Table 1:** Summary of the experiments performed to establish the reaction conditions and the initial mechanistic investigation.

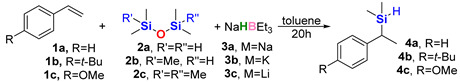		
**Entry**	**Conditions**	**Yield of 4 [%] ^a^**
1	100 °C, [**1a**]:[**2a**]:[**3a**] = 2:1:0.1; toluene added to 1M in **1a**	~7 ^b^
2	[**1a**]:[**2a**]:[**3a**] = 1:1:0.5; otherwise as in *1*	38 ^b^
3	100 °C, [**1a**]:[**2a**]:[**3a**] = 1:1:1; no additional toluene besides the solution of **3a**	81 ^b^
4	[**1a**]:[**2a**]:[**3a**] = 1:2:1.5; otherwise as in *3*	95 ^b^
**5**	[**1b**]:[**2a**]:[**3a**] = 1:2:1.5; otherwise as in *3*	**97**
6	[**1c**]:[**2a**]:[**3a**] = 1:2:1.5; otherwise as in *3*	98
7	80 °C, otherwise as in *5*	94
8	70 °C, otherwise as in *5*	73
9	80 °C; **3b** 1M in THF instead of **3a** , otherwise as in *6*	62 ^c^
10	80 °C; **3c** 1M in THF instead of **3a** , otherwise as in *8*	47 ^d^
11	BEt_3_ instead of **3a** , otherwise as in *6*	- ^e^
12	NaH instead of **3a** , otherwise as in *6*	-
13	80 °C; [**1b**]:[**2a**]:[NaH]:[BEt_3_] = 1:2:1.5:0.1	16%
14	As in *12*; reaction time 120 h	~60% ^f^
15	**2b** instead of **2a** , otherwise as in *6*	93
16	**2c** instead of **2a**, otherwise as in *6*	-

(^a^) Determined using GC and GC-MS; (^b^) **1a** was used; (^c^) isomeric products were present; (^d^) approx. 30% of 1-ethyl-4-*tert*-butylbenzene was detected using GC-MS; (^e^) styrene polymerization; (^f^) after 120 h of running *12*.

**Table 2:** Gibbs free energies (in kcal/mol, relative to separated substrates) in the activation of different styrenes.

Substrate	ΔG_I_	ΔG_II_	ΔG_IV_	ΔΔG_I⭢II_
Styrene	+7.02	+29.78	−11.15	+22.02
4-methoxystyrene	+10.69	+28.53	−11.29	+17.84
4-(trifuloromethyl)styrene	+9.29	+21.47	−17.38	+12.18

## Data Availability

The data presented in this study are available in the Supplementary Materials.

## Supplementary Materials

The following supporting information can be downloaded at: https://www.mdpi.com/article/10.3390/ijms24054924/s1.

## References

[1] Gong Y., Mou Q., Peng D., Wang F., Qin J., Qin J., Ding Y. (2022). New Insight into the Mechanism of Pt(0)-Catalyzed Hydrosilylation Reaction of (CH_3_)_3_SiH with CH_2_CHSi(CH_3_)_3_. J. Mol. Graph. Model..

[2] Xie H., Kuang J., Wang L., Li Y., Huang L., Fan T., Lei Q., Fang W. (2017). A DFT Study on Palladium and Nickel-Catalyzed Regioselective and Stereoselective Hydrosilylation of 1,3-Disubstituted Allenes. Organometallics.

[3] Wang Y.-F., He Y.-H., Su Y., Ji Y., Li R. (2022). Asymmetric Hydrosilylation of β-Silyl Styrenes Catalyzed by a Chiral Palladium Complex. J. Org. Chem..

[4] Wang J., Wang W., Huang L., Yang X., Wei H. (2015). The Unexpected Mechanism Underlying the High-Valent Mono-Oxo-Rhenium(V) Hydride Catalyzed Hydrosilylation of C==N Functionalities: Insights from a DFT Study. ChemPhysChem.

[5] Hassen S., Zouaghi M.O., Slimani I., Arfaoui Y., Özdemir N., Özdemir I., Gürbüz N., Mansour L., Gatri R., Hamdi N. (2022). Synthesis, Crystal Structures, DFT Calculations, and Catalytic Application in Hydrosilylation of Acetophenone Derivatives with Triethylsilane of Novel Rhoduim-N-Heterocyclic Carbene (NHCs) Complex. J. Mol. Struct..

[6] Tuttle T., Wang D., Thiel W., Köhler J., Hofmann M., Weis J. (2006). Mechanism of Olefin Hydrosilylation Catalyzed by RuCl_2_(CO)_2_(PPh_3_)_2_: A DFT Study. Organometallics.

[7] Tuttle T., Wang D., Thiel W., Köhler J., Hofmann M., Weis J. (2007). Mechanism of Olefin Hydrosilylation Catalyzed by [RuCl(NCCH3)5]+: A DFT Study. J. Organomet. Chem..

[8] Wang J., Huang L., Yang X., Wei H. (2015). Mechanistic Investigation Into Catalytic Hydrosilylation with a High-Valent Ruthenium(VI)–Nitrido Complex: A DFT Study. Organometallics.

[9] Yao L., Li Y., Huang L., Guo K., Ren G., Wu Z., Lei Q., Fang W., Xie H. (2018). A DFT Study on the Mechanisms of Hydrogenation and Hydrosilylation of Nitrous Oxide Catalyzed by a Ruthenium PNP Pincer Complex. Comput. Theor. Chem..

[10] Zhang X.-H., Chung L.W., Lin Z., Wu Y.-D. (2008). A DFT Study on the Mechanism of Hydrosilylation of Unsaturated Compounds with Neutral Hydrido(Hydrosilylene)Tungsten Complex. J. Org. Chem..

[11] Drees M., Strassner T. (2007). Mechanism of the MoO_2_ Cl_2_-Catalyzed Hydrosilylation: A DFT Study. Inorg. Chem..

[12] Chen H., Wang W., Wei H. (2018). DFT Study on Mechanism of Carbonyl Hydrosilylation Catalyzed by High-Valent Molybdenum (IV) Hydrides. Tetrahedron.

[13] Itabashi T., Arashiba K., Tanaka H., Yoshizawa K., Nishibayashi Y. (2022). Hydroboration and Hydrosilylation of a Molybdenum–Nitride Complex Bearing a PNP-Type Pincer Ligand. Organometallics.

[14] Bories C.C., Barbazanges M., Derat E., Petit M. (2021). Implication of a Silyl Cobalt Dihydride Complex as a Useful Catalyst for the Hydrosilylation of Imines. ACS Catal..

[15] Zhang J., Lu B., Meng L., Li X. (2022). Charge-Regulated Regioselective Mechanism of Bicobalt-Catalyzed Hydrogermylation of Alkynes: DFT Investigation. Mol. Catal..

[16] Fan G., Shang Z., Li R., Shafiei-Haghighi S., Peng Q., Findlater M., Xu X. (2019). Mechanism of the Iron(0)-Catalyzed Hydrosilylation of Aldehydes: A Combined DFT and Experimental Investigation. Organometallics.

[17] Li Q., Huo S., Meng L., Li X. (2022). Mechanism and Origin of the Stereoselectivity of Manganese-Catalyzed Hydrosilylation of Alkynes: A DFT Study. Catal. Sci. Technol..

[18] Barros N., Eisenstein O., Maron L. (2010). Catalytic Hydrosilylation of Olefins with Organolanthanides: A DFT Study. Part I: Hydrosilylation of Propene by SiH4. Dalton Trans..

[19] Chen W., Jiang C., Zhang J., Xu J., Xu L., Xu X., Li J., Cui C. (2021). Rare-Earth-Catalyzed Selective 1,4-Hydrosilylation of Branched 1,3-Enynes Giving Tetrasubstituted Silylallenes. J. Am. Chem. Soc..

[20] Gajewy J., Gawronski J., Kwit M. (2013). Mechanism and Enantioselectivity of [Zinc(Diamine)(Diol)]-Catalyzed Asymmetric Hydrosilylation of Ketones: DFT, NMR and ECD Studies: Asymmetric Hydrosilylation of Ketones. Eur. J. Org. Chem..

[21] Sakata K., Fujimoto H. (2013). Quantum Chemical Study of B(C _6_ F _5_ ) _3_ -Catalyzed Hydrosilylation of Carbonyl Group. J. Org. Chem..

[22] Nowicki M., Zaranek M., Pawluć P., Hoffmann M. (2020). DFT Study of Trialkylborohydride-Catalysed Hydrosilylation of Alkenes–the Mechanism and Its Implications. Catal. Sci. Technol..

[23] Simonneau A., Oestreich M. (2015). Formal SiH4 Chemistry Using Stable and Easy-to-Handle Surrogates. Nat. Chem..

[24] Simonneau A., Oestreich M. (2013). 3-Silylated Cyclohexa-1,4-Dienes as Precursors for Gaseous Hydrosilanes: The B(C_6_F_5_)_3_-Catalyzed Transfer Hydrosilylation of Alkenes. Angew. Chem. Int. Ed..

[25] Oestreich M. (2016). Transfer Hydrosilylation. Angew. Chem. Int. Ed..

[26] Walker J.C.L., Oestreich M. (2019). Ionic Transfer Reactions with Cyclohexadiene-Based Surrogates. Synlett.

[27] Yuan W., Orecchia P., Oestreich M. (2017). Cyclohexa-1,3-Diene-Based Dihydrogen and Hydrosilane Surrogates in B(C_6_F_5_)_3_-Catalysed Transfer Processes. Chem. Commun..

[28] Keess S., Simonneau A., Oestreich M. (2015). Direct and Transfer Hydrosilylation Reactions Catalyzed by Fully or Partially Fluorinated Triarylboranes: A Systematic Study. Organometallics.

[29] Chauvier C., Thuéry P., Cantat T. (2016). Silyl Formates as Surrogates of Hydrosilanes and Their Application in the Transfer Hydrosilylation of Aldehydes. Angew. Chem. Int. Ed..

[30] Romero R.M., Thyagarajan N., Hellou N., Chauvier C., Godou T., Anthore-Dalion L., Cantat T. (2022). Silyl Formates as Hydrosilane Surrogates for the Transfer Hydrosilylation of Ketones. Chem. Commun..

[31] Buslov I., Keller S.C., Hu X. (2016). Alkoxy Hydrosilanes As Surrogates of Gaseous Silanes for Hydrosilylation of Alkenes. Org. Lett..

[32] Hashimoto T., Shiota K., Ishimaru T., Yamaguchi Y. (2021). Hydrosilylation of Alkenes Using a Hydrosiloxane as a Surrogate for Me_2_SiH_2_ and Catalyzed by a Nickel-Pincer Complex. Eur. J. Org. Chem.

[33] Zaranek M., Nowicki M., Andruszak P., Hoffmann M., Pawluć P. (2022). Hydrogermylation Initiated by Trialkylborohydrides: A Living Anionic Mechanism. Chem. Commun..

[34] Zhao Y., Truhlar D.G. (2008). The M06 Suite of Density Functionals for Main Group Thermochemistry, Thermochemical Kinetics, Noncovalent Interactions, Excited States, and Transition Elements: Two New Functionals and Systematic Testing of Four M06-Class Functionals and 12 Other Functionals. Theor. Chem. Acc..

[35] Leszczynski J., Shukla M. (2012). Practical Aspects of Computational Chemistry II: An Overview of the Last Two Decades and Current Trends.

[36] Pawłowska A., Volle J.-N., Virieux D., Pirat J.-L., Janiak A., Nowicki M., Hoffmann M., Pluskota-Karwatka D. (2018). Perfluorophenyl Phosphonate Analogues of Aromatic Amino Acids: Synthesis, X-Ray and DFT Studies. Tetrahedron.

[37] Macgregor S.A., Eisenstein O. (2016). Computational Studies in Organometallic Chemistry.

[38] Ditchfield R., Hehre W.J., Pople J.A. (1971). Self-Consistent Molecular-Orbital Methods. IX. An Extended Gaussian-Type Basis for Molecular-Orbital Studies of Organic Molecules. J. Chem. Phys..

[39] Clark T., Chandrasekhar J., Spitznagel G.W., Schleyer P.V.R. (1983). Efficient Diffuse Function-Augmented Basis Sets for Anion Calculations. III. The 3-21+G Basis Set for First-Row Elements, Li-F. J. Comput. Chem..

[40] Dill J.D., Pople J.A. (1975). Self-consistent Molecular Orbital Methods. XV. Extended Gaussian-type Basis Sets for Lithium, Beryllium, and Boron. J. Chem. Phys..

[41] Hehre W.J., Ditchfield R., Pople J.A. (1972). Self—Consistent Molecular Orbital Methods. XII. Further Extensions of Gaussian—Type Basis Sets for Use in Molecular Orbital Studies of Organic Molecules. J. Chem. Phys..

[42] Gordon M.S. (1980). The Isomers of Silacyclopropane. Chem. Phys. Lett..

[43] Francl M.M., Pietro W.J., Hehre W.J., Binkley J.S., Gordon M.S., DeFrees D.J., Pople J.A. (1982). Self-consistent Molecular Orbital Methods. XXIII. A Polarization-type Basis Set for Second-row Elements. J. Chem. Phys..

[44] Frisch M.J., Pople J.A., Binkley J.S. (1984). Self-consistent Molecular Orbital Methods 25. Supplementary Functions for Gaussian Basis Sets. J. Chem. Phys..

[45] Frisch M.J., Trucks G.W., Schlegel H.B., Scuseria G.E., Robb M.A., Cheeseman J.R., Scalmani G., Barone V., Petersson G.A., Nakatsuji H. (2016). Gaussian 16 Rev. C.01.

[46] Peng C., Bernhard Schlegel H. (1993). Combining Synchronous Transit and Quasi-Newton Methods to Find Transition States. Isr. J. Chem..

[47] Miertuš S., Scrocco E., Tomasi J. (1981). Electrostatic Interaction of a Solute with a Continuum. A Direct Utilizaion of AB Initio Molecular Potentials for the Prevision of Solvent Effects. Chem. Phys..

[48] Miertuš S., Tomasi J. (1982). Approximate Evaluations of the Electrostatic Free Energy and Internal Energy Changes in Solution Processes. Chem. Phys..

[49] Pascual-ahuir J.L., Silla E., Tuñon I. (1994). GEPOL: An Improved Description of Molecular Surfaces. III. A New Algorithm for the Computation of a Solvent-Excluding Surface: GEPOL. J. Comput. Chem..

[50] Valtazanos P., Ruedenberg K. (1986). Bifurcations and Transition States. Theoret. Chim. Acta.

[51] Ess D.H., Wheeler S.E., Iafe R.G., Xu L., Çelebi-Ölçüm N., Houk K.N. (2008). Bifurcations on Potential Energy Surfaces of Organic Reactions. Angew. Chem. Int. Ed..

