# Supplementary material for: Mechanism of Silylation of Vinyl Arenes by Hydrodisiloxanes Driven by Stoichiometric Amounts of Sodium Triethylborohydride—A Combined DFT and Experimental Study
Source: Int J Mol Sci. 2023 Mar 3;24(5):4924. doi: 10.3390/ijms24054924 (PMC10003527; doi:10.3390/ijms24054924)
Supplement: Supplementary file 1 [file ijms-24-04924-s001.zip › ijms-2181707-supplementary.pdf]

Supplementary information

Mechanism of silylation of vinyl arenes by hydrodisiloxanes driven by stoichiometric amounts of sodium triethylborohydride – a combined DFT and experimental study

Mateusz Nowicki [1], Maciej Zaranek [1,2], Magdalena Grzelak [2], Piotr Pawluć [1,2] and Marcin Hoffmann [1,*]

[1] Faculty of Chemistry, Adam Mickiewicz University, Uniwersytetu Poznańskiego 8, 61-614 Poznań, Poland

[2] Centre for Advanced Technologies, Adam Mickiewicz University, Uniwersytetu Poznańskiego 10, 61-614 Poznań, Poland

* Correspondence: marcin.hoffman@amu.edu.pl

Cartesian coordinates, Mulliken and APT charges of all atoms, and absolute energies at all stationary points

Substrates and products
Styrene activation
1,1,3,3-tetramethyldisiloxane (TMDSO)
1,1,1,3,3-pentamethyldisiloxane (PMDSO)
Hexamethyldisiloxane (HMDSO)
Regeneration of $HBMe_3^-$

Cartesian coordinates, Mulliken and APT charges of all atoms, and absolute energies at all stationary points

All coordinates are given in ångströms.

All energies are given in hartrees.

Substrates and products

TMDSO

Electronic energy: -815.015907

Sum of electronic and zero-point Energies= -814.844844

Sum of electronic and thermal Energies= -814.826362

Sum of electronic and thermal Enthalpies= -814.825180

Sum of electronic and thermal Free Energies= -814.898777

Table S01. Cartesian coordinates, Mulliken and APT charges of all atoms at **TMDSO**.

Symbol	X	Y	Z	Mulliken	APT
Si	-1.601232	-0.090742	-0.489659	0.636859	1.932541
O	0.035332	-0.360704	-0.636964	-0.448368	-1.561537
Si	1.368703	-0.082852	0.319410	0.719192	1.900942
C	2.684596	-1.323041	-0.145280	-0.769215	-0.420905
H	2.326792	-2.346651	-0.003882	0.199543	0.020088
H	2.964387	-1.202614	-1.196575	0.184505	0.019035
H	3.583378	-1.186290	0.464182	0.191441	0.016175
C	1.968112	1.672851	0.071083	-0.776639	-0.431433
H	1.196054	2.398205	0.345371	0.186344	0.018580
H	2.853009	1.872433	0.684168	0.193563	0.017665
H	2.235383	1.840613	-0.977126	0.193508	0.019086
C	-2.355129	-1.393929	0.621907	-0.743880	-0.435726
H	-2.162507	-2.399041	0.235879	0.201750	0.017788
H	-1.932753	-1.329518	1.630215	0.183962	0.020638
H	-3.438962	-1.260597	0.700375	0.186543	0.017969
C	-1.893773	1.620048	0.218368	-0.796231	-0.446820
H	-1.450962	1.705640	1.217352	0.193791	0.019599
H	-1.453941	2.394422	-0.417346	0.214178	0.018231
H	-2.965179	1.825613	0.310722	0.183180	0.022769
H	0.967956	-0.264567	1.743167	-0.033964	-0.388959

H	-2.172749	-0.187273	-1.853782	-0.100064	-0.375726

PMDSO

Electronic energy: -854.329541

Sum of electronic and zero-point Energies= -854.129662

Sum of electronic and thermal Energies= -854.108741

Sum of electronic and thermal Enthalpies= -854.107559

Sum of electronic and thermal Free Energies= -854.184404

Table S02. Cartesian coordinates, Mulliken and APT charges of all atoms at **PMDSO.**

Symbol	X	Y	Z	Mulliken	APT
Si	1.729645	0.000807	-0.600307	0.621463	1.916886
O	0.101156	-0.189862	-0.866905	-0.428692	-1.578933
Si	-1.304755	-0.032010	0.017170	0.815390	2.013187
C	-1.723689	1.788260	0.184543	-0.722005	-0.447407
H	-0.951458	2.321281	0.748897	0.184866	0.013030
H	-1.810263	2.259897	-0.799476	0.196571	0.013826
H	-2.675824	1.921411	0.709279	0.179205	0.014967
C	-2.648300	-0.938423	-0.915279	-0.825139	-0.436961
H	-2.402444	-1.999724	-1.018718	0.189810	0.014604
H	-3.607977	-0.859892	-0.393995	0.174589	0.012907
H	-2.771548	-0.519709	-1.918855	0.186302	0.014793
C	2.042871	1.587418	0.349526	-0.734141	-0.439273
H	1.635318	2.454360	-0.179276	0.210650	0.017647
H	1.579930	1.545596	1.342052	0.197310	0.014541
H	3.116674	1.748955	0.490257	0.184063	0.020261
C	2.397906	-1.465384	0.354646	-0.710350	-0.433855
H	2.164390	-2.404938	-0.154783	0.205805	0.018043
H	3.485576	-1.396643	0.460325	0.188326	0.016572
H	1.962839	-1.505233	1.358817	0.182676	0.019633
H	2.374894	0.071645	-1.933275	-0.124054	-0.375081
C	-1.047083	-0.782834	1.719406	-0.763430	-0.453708
H	-1.964769	-0.719311	2.313939	0.168052	0.021328
H	-0.764409	-1.838134	1.645711	0.204824	0.013091
H	-0.258869	-0.258048	2.271207	0.217910	0.009904

HMDSO

Electronic energy: -893.642711

Sum of electronic and zero-point Energies= -893.414735

Sum of electronic and thermal Energies= -893.390854

Sum of electronic and thermal Enthalpies= -893.389672

Sum of electronic and thermal Free Energies= -893.473371

Table S03. Cartesian coordinates, Mulliken and APT charges of all atoms at **HMDSO**.

Symbol	X	Y	Z	Mulliken	APT
Si	-1.549707	0.002306	0.083630	0.740477	2.019956
O	-0.000047	-0.000066	0.696001	-0.405208	-1.598719
Si	1.549613	-0.002406	0.083716	0.741065	2.019988
C	1.981384	1.723142	-0.512580	-0.697703	-0.449381
H	1.336114	2.027242	-1.343242	0.194409	0.014343
H	1.862830	2.454078	0.293687	0.198173	0.012168
H	3.019424	1.766571	-0.859382	0.176859	0.013790
C	2.692906	-0.524767	1.469374	-0.826980	-0.437005
H	2.439192	-1.527753	1.826152	0.189349	0.013667
H	3.735269	-0.535795	1.134238	0.172637	0.012892
H	2.615425	0.165356	2.315282	0.184504	0.013567
C	-1.651898	1.215685	-1.346863	-0.715406	-0.457050
H	-1.375314	2.225711	-1.026936	0.201297	0.013361
H	-0.984061	0.924478	-2.165441	0.212936	0.010722
H	-2.670176	1.255255	-1.748566	0.171778	0.018216
C	-1.979810	-1.721539	-0.518768	-0.697629	-0.449403
H	-1.861620	-2.455154	0.285125	0.198240	0.012221
H	-3.017581	-1.764212	-0.866498	0.176845	0.013752
H	-1.333838	-2.022592	-1.349967	0.194349	0.014313
C	1.650940	-1.210806	-1.351079	-0.715643	-0.457114
H	2.669406	-1.249704	-1.752391	0.171721	0.018208
H	1.373466	-2.221781	-1.034959	0.201308	0.013350
H	0.983777	-0.916072	-2.168936	0.212994	0.010913
C	-2.693393	0.518451	1.471279	-0.826875	-0.436953
H	-3.735934	0.528908	1.136692	0.172641	0.012953
H	-2.614408	-0.174267	2.314934	0.184503	0.013549
H	-2.441043	1.520658	1.831187	0.189358	0.013695

Styrene

Electronic energy: -309.521915

Sum of electronic and zero-point Energies= -309.387638

Sum of electronic and thermal Energies= -309.378437

Sum of electronic and thermal Enthalpies= -309.377255

Sum of electronic and thermal Free Energies= -309.427176

Table S04. Cartesian coordinates, Mulliken and APT charges of all atoms at **styrene.**

Symbol	X	Y	Z	Mulliken	APT
C	1.780057	-1.042844	-0.000436	0.323588	-0.017353
C	0.406903	-1.281326	-0.000014	-0.816066	-0.080136
C	2.259356	0.265026	-0.000289	-0.515173	-0.069084
C	-0.511471	-0.223716	0.000408	1.297441	0.047501
C	-0.014625	1.088505	0.000562	-1.446540	-0.084226
C	1.354787	1.329411	0.000184	0.386121	-0.037724
H	2.473140	-1.878318	-0.000951	0.164729	0.038071
H	3.327586	0.456765	-0.000426	0.145247	0.042962
H	1.719887	2.351884	0.000276	0.177083	0.037016
H	-0.701104	1.929291	0.000821	0.043929	0.045572
H	0.036731	-2.303435	-0.000188	0.116777	0.042650
C	-1.954308	-0.535184	0.000724	-0.034288	0.127216
C	-2.964541	0.339188	-0.000908	-0.255580	-0.209170
H	-2.190259	-1.598490	0.002181	0.115982	0.015937
H	-2.810355	1.413956	-0.002530	0.134337	0.054622
H	-3.992581	-0.006024	-0.000577	0.162414	0.046145

$NaHBMe_3$

Electronic energy: -307.416588

Sum of electronic and zero-point Energies= -307.292494

Sum of electronic and thermal Energies= -307.280554

Sum of electronic and thermal Enthalpies= -307.279372

Sum of electronic and thermal Free Energies= -307.334624

Table S05. Cartesian coordinates, Mulliken and APT charges of all atoms at **$NaHBMe_3$.**

Symbol	X	Y	Z	Mulliken	APT
H	0.128001	0.001651	-1.355141	-0.068023	-0.441930
C	-0.047094	1.373227	0.610463	-0.669985	-0.162796
H	1.004722	1.546222	0.914825	0.087507	-0.136683
H	-0.347940	2.281109	0.070708	0.162214	-0.056161
H	-0.601461	1.373483	1.559923	0.153751	-0.045180
C	-0.031321	-1.363753	0.617789	-0.675930	-0.164145
H	-0.610353	-1.378479	1.552137	0.153876	-0.043951
H	-0.294791	-2.279345	0.071490	0.162590	-0.056470
H	1.015190	-1.508475	0.953499	0.088986	-0.137209
C	-2.046952	-0.010229	-0.515237	-0.788735	-0.154058
H	-2.380510	0.868669	-1.084082	0.148127	-0.067233
H	-2.369674	-0.897661	-1.077013	0.147970	-0.067095
H	-2.612985	-0.009830	0.429282	0.133532	-0.065778
B	-0.444083	0.000745	-0.230303	0.203570	0.706835
Na	2.003856	0.000315	-0.469291	0.760551	0.891855

$HBMe_3^-$

Electronic energy: -145.196524

Sum of electronic and zero-point Energies= -145.074736

Sum of electronic and thermal Energies= -145.065470

Sum of electronic and thermal Enthalpies= -145.064288

Sum of electronic and thermal Free Energies= -145.111799

Table S06. Cartesian coordinates, Mulliken and APT charges of all atoms at **$HBMe_3^-$.**

Symbol	X	Y	Z	Mulliken	APT
C	-1.186566	1.009803	-0.095878	-0.806663	-0.142209
B	0.000155	0.000460	0.445253	0.672261	0.764594
C	1.468737	0.521580	-0.096402	-0.805971	-0.146608
C	-0.282448	-1.530981	-0.096005	-0.805615	-0.141511
H	0.004279	0.000109	1.692708	-0.418749	-0.445054
H	0.477194	-2.253162	0.244573	0.132117	-0.099089
H	-0.277267	-1.568683	-1.199766	0.124620	-0.097030
H	-1.259537	-1.925644	0.226703	0.131364	-0.101632
H	2.300286	-0.120412	0.236722	0.132210	-0.100001
H	1.708581	1.545631	0.232588	0.131632	-0.096933
H	1.502213	0.530349	-1.200267	0.124754	-0.096778

H	-1.216377	1.027286	-1.199611	0.125121	-0.095879
H	-1.046541	2.052972	0.230957	0.131206	-0.100999
H	-2.191942	0.706840	0.238832	0.131713	-0.100871

BMe_3

Electronic energy: -144.531104

Sum of electronic and zero-point Energies= -144.417891

Sum of electronic and thermal Energies= -144.407714

Sum of electronic and thermal Enthalpies= -144.406532

Sum of electronic and thermal Free Energies= -144.459688

Table S07. Cartesian coordinates, Mulliken and APT charges of all atoms at **BMe_3.**

Symbol	X	Y	Z	Mulliken	APT
C	0.838143	1.333921	0.003204	-0.732597	-0.304270
H	0.252828	2.251646	0.109042	0.164817	-0.019411
H	1.601051	1.308488	0.791880	0.178412	0.012106
H	1.401814	1.394030	-0.938537	0.181994	0.015997
C	-1.577069	0.057410	0.004558	-0.730231	-0.304672
H	-2.076001	-0.911001	0.101809	0.165933	-0.018223
H	-1.946672	0.729889	0.788343	0.177352	0.009244
H	-1.904031	0.511886	-0.942152	0.182307	0.019140
C	0.737943	-1.391652	-0.006728	-0.732049	-0.304998
H	1.815505	-1.341691	-0.187739	0.166089	-0.017155
H	0.586894	-1.853004	0.980771	0.183706	0.019009
H	0.284497	-2.087477	-0.722582	0.175995	0.007778
B	-0.001998	-0.000169	0.002593	0.618273	0.885455

NaH

Electronic energy: -162.828635

Sum of electronic and zero-point Energies= -162.826347

Sum of electronic and thermal Energies= -162.823296

Sum of electronic and thermal Enthalpies= -162.822114

Sum of electronic and thermal Free Energies= -162.849960

Table S08. Cartesian coordinates, Mulliken and APT charges of all atoms at **NaH.**

Symbol	X	Y	Z	Mulliken	APT
Na	0.000000	0.000000	0.164166	0.731584	0.816239
H	-0.000000	-0.000000	-1.805825	-0.731584	-0.816239

$NaBMe_3OSiMe_2H$

Electronic energy: -751.989377

Sum of electronic and zero-point Energies= -751.786042

Sum of electronic and thermal Energies= -751.765075

Sum of electronic and thermal Enthalpies= -751.763893

Sum of electronic and thermal Free Energies= -751.840291

Table S09. Cartesian coordinates, Mulliken and APT charges of all atoms at **$NaBMe_3OSiMe_2H$** in the reaction of styrene with TMDSO.

Symbol	X	Y	Z	Mulliken	APT
O	-0.003496	-0.290546	-0.187988	-0.706798	-1.480894
Si	1.503838	0.134020	0.323943	0.509591	1.854714
C	2.578032	-1.413170	0.263066	-0.808646	-0.409765
H	2.183754	-2.204389	0.909761	0.177577	-0.006613
H	2.641881	-1.802517	-0.759461	0.176817	-0.002071
H	3.597897	-1.193784	0.595825	0.189043	0.008524
C	2.299011	1.450737	-0.752852	-0.722434	-0.426880
H	1.751085	2.395007	-0.701020	0.200451	0.028796
H	3.330968	1.633583	-0.433503	0.184718	0.001780
H	2.319888	1.127279	-1.799145	0.188247	0.005753
H	1.471106	0.620956	1.732737	-0.043018	-0.393556
B	-1.326514	0.488568	0.112290	1.143419	1.119011
C	-1.050176	2.094694	0.097195	-0.965810	-0.229691
H	-0.296417	2.411165	0.833950	0.145261	-0.055884
H	-0.712749	2.449624	-0.887487	0.145996	-0.058011
H	-1.968358	2.647229	0.338115	0.123061	-0.041231
C	-1.871994	-0.014358	1.574952	-0.831075	-0.207353
H	-2.827889	0.462928	1.830301	0.142639	-0.042033

H	-2.055731	-1.102616	1.653192	0.105577	-0.119358
H	-1.165082	0.232278	2.379409	0.154482	-0.047625
C	-2.397426	0.114671	-1.090790	-0.786670	-0.215136
H	-2.903193	-0.870429	-1.049905	0.109957	-0.125161
H	-3.232082	0.825790	-1.057462	0.159844	-0.024706
H	-1.946529	0.211555	-2.089334	0.157622	-0.052494
Na	-1.084220	-2.112169	-0.512559	0.850147	0.919883

$NaBMe_3OSiMe_3$

Electronic energy: -791.301672

Sum of electronic and zero-point Energies= -791.069951

Sum of electronic and thermal Energies= -791.045083

Sum of electronic and thermal Enthalpies= -791.043901

Sum of electronic and thermal Free Energies= -791.130454

Table S10. Cartesian coordinates, Mulliken and APT charges of all atoms at **$NaBMe_3OSiMe_3$** in the reaction of styrene with TMDSO.

Symbol	X	Y	Z	Mulliken	APT
O	0.202103	0.381780	-0.229329	-0.737028	-1.510191
Si	-1.393435	0.014422	-0.027569	0.588922	1.970291
C	-2.331604	1.644219	-0.195936	-0.888507	-0.423024
H	-2.030871	2.364047	0.574415	0.161499	-0.015076
H	-2.161641	2.099142	-1.178306	0.175066	-0.004542
H	-3.410372	1.486392	-0.089157	0.172224	0.005078
C	-2.046375	-1.164426	-1.339860	-0.650486	-0.445030
H	-1.669135	-2.180189	-1.193792	0.212386	0.025521
H	-3.141410	-1.197926	-1.306044	0.169748	-0.000639
H	-1.747753	-0.834864	-2.340641	0.192251	0.005701
B	1.452602	-0.520902	-0.003355	1.111327	1.123130
C	1.116700	-2.074070	-0.366449	-0.940791	-0.225722
H	0.311418	-2.500636	0.249592	0.179904	-0.060322
H	0.822223	-2.202800	-1.418100	0.139449	-0.056947
H	1.999878	-2.706021	-0.201683	0.115826	-0.042378
C	1.909753	-0.356100	1.563954	-0.785220	-0.200459
H	2.829974	-0.919330	1.771718	0.146954	-0.044960
H	2.124372	0.684184	1.873677	0.117407	-0.119680
H	1.142414	-0.726674	2.257630	0.176328	-0.049843

C	2.636549	0.023310	-1.024442	-0.788043	-0.215942
H	3.175306	0.951086	-0.745270	0.110603	-0.125831
H	3.436878	-0.725154	-1.077856	0.159851	-0.026938
H	2.265889	0.156847	-2.051389	0.156904	-0.053204
Na	1.366566	2.167400	-0.080183	0.845983	0.918868
C	-1.757833	-0.693725	1.678325	-0.695237	-0.451486
H	-1.436587	-0.004696	2.467042	0.185192	-0.001035
H	-2.833934	-0.865148	1.796797	0.169522	0.001434
H	-1.243757	-1.646543	1.837205	0.197964	0.023222

Styrene activation

I

Electronic energy: -454.727438

Electronic energy + zero-point energy: -454.469600

Electronic energy + thermal energy correction: -454.448794

Electronic energy + thermal enthalpy correction: -454.447612

Electronic energy + thermal free energy correction: -454.525329

Table S11. Cartesian coordinates, Mulliken and APT charges of all atoms at **I** in the reaction of styrene with disiloxanes.

Symbol	X	Y	Z	Mulliken	APT
C	-3.779070	-0.539130	0.088559	-0.274053	-0.035519
C	-3.128640	0.693370	0.074239	0.256598	-0.078645
C	-3.041980	-1.713300	-0.051691	0.015569	-0.072276
C	-1.738450	0.777590	-0.081391	0.671882	0.063431
C	-1.004780	-0.411330	-0.224591	-1.048043	-0.103224
C	-1.655790	-1.639980	-0.207831	-0.169335	-0.026664
H	-4.858100	-0.581200	0.211609	0.135160	0.018444
H	-3.541870	-2.677940	-0.036951	0.140951	0.023276
H	-1.068080	-2.547600	-0.311891	0.170941	0.036487
H	0.076110	-0.380810	-0.349061	-0.068035	0.144698
H	-3.704370	1.610060	0.186979	0.128765	0.024771
C	-1.090020	2.103020	-0.082431	-0.202878	0.065997
C	0.220000	2.336230	-0.217561	-0.149507	-0.176922
H	-1.770080	2.946440	0.046769	0.101007	-0.010619
H	0.957560	1.545700	-0.351321	0.099196	0.140046

H	0.594130	3.355950	-0.196621	0.154966	0.020045
C	3.798500	-1.624480	-0.568011	-0.851289	-0.139150
B	2.863070	-0.331250	-0.162401	0.496460	0.725739
C	3.792730	1.027200	-0.054421	-0.836767	-0.147311
C	2.116270	-0.613410	1.282489	-0.897735	-0.159595
H	2.011140	-0.169280	-1.062891	-0.184584	-0.456282
H	1.454080	0.205640	1.612989	0.203771	-0.093447
H	2.869080	-0.739420	2.079449	0.125328	-0.102253
H	1.505580	-1.531910	1.284789	0.168399	-0.087547
H	3.226010	1.927800	0.236169	0.170534	-0.088335
H	4.304740	1.265600	-1.000421	0.130619	-0.097253
H	4.580770	0.901490	0.708449	0.131752	-0.098784
H	4.575540	-1.810200	0.194209	0.124634	-0.101162
H	4.323170	-1.482640	-1.526281	0.115987	-0.099750
H	3.216630	-2.556100	-0.658121	0.139706	-0.088196

II

Electronic energy: -454.700826

Electronic energy + zero-point energy: -454.446921

Electronic energy + thermal energy correction: -454.427491

Electronic energy + thermal enthalpy correction: -454.426310

Electronic energy + thermal free energy correction: -454.500030

Table S12. Cartesian coordinates, Mulliken and APT charges of all atoms at **II** in the reaction of styrene with disiloxanes.

Symbol	X	Y	Z	Mulliken	APT
C	-3.836028	-0.300143	0.447149	-0.290771	0.200546
C	-2.749528	-1.159252	0.362529	0.013418	-0.407050
C	-3.713589	1.047528	0.090699	-0.003765	-0.381405
C	-1.478728	-0.721061	-0.089091	0.007262	0.826438
C	-1.369779	0.655479	-0.413801	-0.700878	-0.263578
C	-2.464709	1.508358	-0.334721	-0.293342	0.193759
H	-4.793428	-0.683153	0.795009	0.118790	-0.003172
H	-4.563039	1.720857	0.157649	0.126408	0.013058
H	-2.335039	2.557698	-0.591191	0.144886	0.014128
H	-0.401119	1.056239	-0.699831	-0.006996	0.042478
H	-2.867347	-2.205572	0.638359	0.111435	0.008162

C	-0.369718	-1.629131	-0.190331	0.719516	-1.277510
C	0.850832	-1.288550	-0.764431	-0.749325	1.417320
H	-0.461127	-2.581611	0.326749	0.129283	-0.019362
H	0.877862	-0.521430	-1.540021	0.214600	-0.056486
H	1.584483	-2.077170	-0.917721	0.173368	-0.038891
C	3.266122	0.155291	1.772009	-0.735345	-0.327757
B	2.874902	0.250431	0.198469	0.961615	1.406704
C	3.933252	-0.476679	-0.798091	-0.888591	-0.266207
C	2.437451	1.733960	-0.301041	-0.899247	-0.303077
H	1.748792	-0.490680	0.094619	-0.437873	-1.104247
H	2.017891	1.734910	-1.319801	0.147944	-0.057976
H	3.314791	2.401981	-0.330071	0.115317	-0.092316
H	1.697091	2.206900	0.359009	0.165278	-0.050778
H	3.574712	-0.525409	-1.838171	0.133186	-0.046415
H	4.179202	-1.503609	-0.488491	0.144251	-0.066457
H	4.883182	0.082091	-0.823191	0.125966	-0.122862
H	4.184661	0.733341	1.972639	0.137647	-0.132499
H	3.465262	-0.875379	2.098709	0.155863	-0.061582
H	2.485191	0.563050	2.428139	0.160103	-0.042966

III

Electronic energy: -454.710828

Electronic energy + zero-point energy: -454.454449

Electronic energy + thermal energy correction: -454.432180

Electronic energy + thermal enthalpy correction: -454.430998

Electronic energy + thermal free energy correction: -454.512741

Table S13. Cartesian coordinates, Mulliken and APT charges of all atoms at **III** in the reaction of styrene with disiloxanes.

Symbol	X	Y	Z	Mulliken	APT
C	3.341920	-0.333953	-0.937640	-0.148479	0.375908
C	2.226100	-1.095712	-0.664840	0.059833	-0.415358
C	3.578071	0.900157	-0.295740	-0.292088	-0.735671
C	1.221120	-0.692081	0.292480	-0.182014	0.788465
C	1.490661	0.576359	0.931290	-0.362754	-0.517683
C	2.622582	1.323868	0.636400	-0.164380	0.473369
H	4.059740	-0.701093	-1.671770	0.106138	-0.018578

H	4.458312	1.495036	-0.518510	0.104046	0.005569
H	2.765592	2.274238	1.151950	0.109065	-0.014572
H	0.781371	0.958959	1.661920	0.122264	0.010870
H	2.081059	-2.042702	-1.183870	0.113910	-0.015724
C	0.090750	-1.454020	0.565350	-0.211581	-0.981922
C	-0.909830	-1.047050	1.599650	-0.771787	0.453852
H	-0.030461	-2.414970	0.071050	0.075412	-0.018766
H	-0.466530	-0.883840	2.599970	0.144757	-0.143260
H	-1.695381	-1.803889	1.715800	0.140378	-0.053553
C	-3.219440	-1.021788	-0.993000	-0.818407	-0.331939
B	-2.890439	0.454542	-0.540860	0.920316	0.891533
C	-3.802438	1.185982	0.528090	-0.825698	-0.315281
C	-1.666398	1.170891	-1.213470	-0.802103	-0.338750
H	-1.429909	-0.089999	1.370760	0.133732	-0.191249
H	-1.569908	2.239581	-0.998010	0.137632	-0.026887
H	-1.632219	1.015891	-2.299100	0.142079	-0.005690
H	-0.763869	0.662500	-0.827210	0.210604	0.089736
H	-3.304978	2.016242	1.039440	0.170446	-0.006159
H	-4.220799	0.501123	1.274120	0.168892	-0.006492
H	-4.659458	1.612063	-0.019470	0.157185	-0.013486
H	-3.348260	-1.085458	-2.081370	0.148938	-0.003598
H	-4.086960	-1.480057	-0.505940	0.138880	-0.043487
H	-2.323731	-1.622249	-0.766920	0.274781	0.108802

IV

Electronic energy: -454.772817

Electronic energy + zero-point energy: -454.513658

Electronic energy + thermal energy correction: -454.493209

Electronic energy + thermal enthalpy correction: -454.492027

Electronic energy + thermal free energy correction: -454.569791

Table S14. Cartesian coordinates, Mulliken and APT charges of all atoms at **IV** in the reaction of styrene with disiloxanes.

Symbol	X	Y	Z	Mulliken	APT
C	2.706540	1.131190	-0.502080	-0.242638	0.058156
C	1.385300	0.920020	-0.885630	0.525437	-0.149807
C	3.357190	0.216300	0.328120	-0.051631	-0.187616

C	0.655550	-0.204820	-0.458850	-0.749226	0.318115
C	1.331670	-1.110860	0.377710	-0.305010	-0.167752
C	2.654650	-0.906010	0.765760	-0.225500	0.059943
H	3.233540	2.015760	-0.853370	0.127672	0.005626
H	4.389180	0.377020	0.628480	0.126734	0.013490
H	3.139280	-1.629000	1.418450	0.129110	0.006171
H	0.800730	-1.986420	0.742090	0.124766	0.034671
H	0.886570	1.644370	-1.526250	0.150543	0.025385
C	-0.776720	-0.387590	-0.842640	-0.192834	-0.389968
C	-1.143550	-1.862230	-1.054100	-0.526023	0.121324
H	-0.927720	0.140130	-1.798540	0.040982	-0.096164
H	-0.388210	-2.410760	-1.637690	0.149350	-0.090750
H	-2.099810	-1.936600	-1.581210	0.140281	-0.039910
C	-3.374330	0.065860	-0.215910	-0.870477	-0.161143
B	-1.824340	0.374160	0.259490	0.795261	0.773735
C	-1.585040	-0.190210	1.787610	-0.797555	-0.171509
C	-1.571100	2.000390	0.228930	-0.830100	-0.163754
H	-1.271940	-2.387800	-0.099300	0.164946	-0.026888
H	-0.567730	2.292280	0.575600	0.185391	-0.070491
H	-2.292700	2.514780	0.884180	0.115817	-0.097881
H	-1.701640	2.429420	-0.779750	0.150262	-0.087220
H	-0.591380	0.065340	2.187250	0.190707	-0.071615
H	-1.695290	-1.284030	1.879810	0.147637	-0.079837
H	-2.325360	0.251120	2.474290	0.123981	-0.091676
H	-4.084320	0.661670	0.380040	0.121438	-0.103705
H	-3.670790	-0.987850	-0.091770	0.135657	-0.082567
H	-3.551690	0.327550	-1.273320	0.145022	-0.086361

1,1,3,3-tetramethyldisiloxane (TMDSO)

V

Electronic energy: -1269.799326

Electronic energy + zero-point energy: -1269.366835

Electronic energy + thermal energy correction: -1269.328638

Electronic energy + thermal enthalpy correction: -1269.327456

Electronic energy + thermal free energy correction: -1269.442082

Table S15. Cartesian coordinates, Mulliken and APT charges of all atoms at **V** in the reaction of styrene with TMDSO.

Symbol	X	Y	Z	Mulliken	APT
Si	-0.087487	1.819706	-1.866917	1.470247	-0.374020
O	-0.390188	-1.505022	-3.417298	1.026912	0.095784
Si	0.998799	1.863018	-4.041323	-0.381138	0.703308
C	-0.757835	-0.424703	-3.997729	-1.727741	-0.599854
H	0.190364	0.004433	-4.573012	-1.440864	-1.486670
H	0.178215	0.039758	-2.962197	-1.909591	-0.912021
H	0.185060	0.008099	-4.408156	-2.668637	-0.214303
C	-0.835933	-0.425050	-3.078126	-0.922366	2.220817
H	0.202470	0.010411	-3.076753	-0.143238	2.991076
H	0.170768	0.011844	-3.502603	-1.834232	2.657479
H	0.209734	0.024189	-2.035674	-1.133114	1.952851
C	-0.729547	-0.404003	-2.054065	2.969972	-1.485255
H	0.173577	0.002998	-2.538315	3.791126	-0.944571
H	0.171165	0.019484	-1.074570	3.319079	-1.831010
H	0.179175	0.009895	-2.663021	2.734788	-2.364375
C	-0.553529	-0.429977	-0.879065	1.913160	1.152518
H	0.173856	-0.000736	-1.378729	2.718598	1.704957
H	0.227189	0.031690	-0.765157	1.052556	1.820353
H	0.240220	0.049122	0.129548	2.245864	0.883733
C	-0.014991	-0.440236	2.424675	0.621379	0.270626
H	0.117393	-0.069761	2.035394	0.893792	1.265541
C	-0.532719	0.131370	2.510838	1.892936	-0.583226
H	0.156814	-0.024644	3.172365	1.753912	-1.448222
H	0.162814	-0.105128	1.532221	2.223838	-0.972254
H	0.138034	-0.037014	2.934238	2.713779	0.005888
C	-0.747622	0.304132	1.499043	-0.393587	-0.310464
C	0.007566	-0.169753	0.720104	-1.227807	0.513681
C	-0.244699	-0.166832	1.392935	-0.603795	-1.697067
C	-0.316628	0.029284	-0.114314	-2.210962	-0.011602
H	0.131581	0.035477	0.793826	-1.099630	1.593192
C	-0.220252	0.063820	0.561045	-1.586906	-2.229880
H	0.134137	0.039332	1.989128	0.009927	-2.368048
C	-0.094957	-0.193780	-0.203404	-2.399220	-1.392948
H	0.134711	0.015554	-0.696588	-2.841578	0.659106
H	0.156124	0.009791	0.509757	-1.719560	-3.308801
H	0.152320	0.022282	-0.853103	-3.166785	-1.807187
H	-0.176666	-0.384907	-5.452129	-0.097359	1.084930
H	0.226510	-0.293868	-1.278185	0.336747	-1.122264
B	1.057631	0.803482	3.965887	-0.039690	0.571757

C	-0.805104	-0.167860	3.836033	-1.239022	1.690768
H	0.168633	-0.065611	3.256770	-2.107611	1.337652
H	0.119432	-0.099546	4.833582	-1.619918	1.966399
H	0.131224	-0.088814	3.362260	-0.897655	2.628854
C	-0.859834	-0.166946	4.916800	1.164281	1.175773
H	0.134237	-0.077662	5.153262	1.950442	0.439675
H	0.141261	-0.084866	4.461678	1.668127	2.046987
H	0.120928	-0.112545	5.883291	0.757935	1.517432
C	-0.752415	-0.177775	4.630224	-0.642365	-0.808056
H	0.132252	-0.079655	4.722754	0.098743	-1.620992
H	0.128157	-0.091012	5.652603	-1.003643	-0.607012
H	0.168057	-0.061464	4.069873	-1.497954	-1.218451

VIa

Electronic energy: -1269.749890

Electronic energy + zero-point energy: -1269.317809

Electronic energy + thermal energy correction: -1269.277661

Electronic energy + thermal enthalpy correction: -1269.276480

Electronic energy + thermal free energy correction: -1269.397609

Table S16. Cartesian coordinates, Mulliken and APT charges of all atoms at **VIa** in the reaction of styrene with TMDSO.

Symbol	X	Y	Z	Mulliken	APT
Si	0.555427	2.229913	1.316688	-1.159169	0.326629
O	-0.506155	-1.588875	3.060059	-1.277267	0.405035
Si	0.709043	1.884571	4.145516	-0.098902	0.061476
C	-0.730519	-0.418010	4.441225	0.056631	-1.793709
H	0.190549	0.000692	4.762225	-0.899716	-2.221306
H	0.177632	0.020046	3.515374	0.357362	-2.297297
H	0.171059	-0.010377	5.210580	0.807007	-2.013817
C	-0.750686	-0.420235	3.587673	1.574867	0.725074
H	0.197674	0.005327	3.448207	1.541336	1.811509
H	0.159249	-0.003774	4.322244	2.358428	0.500945
H	0.238277	0.018900	2.629624	1.870895	0.278074
C	-0.795156	-0.446090	0.930431	-2.994165	0.087883
H	0.147544	-0.043014	1.691201	-3.570685	0.627367

H	0.161176	0.013728	-0.061895	-3.267070	0.459387
H	0.170452	0.003260	0.987391	-3.274211	-0.969832
C	-0.827668	-0.459446	0.976550	-0.561248	2.088062
H	0.143449	-0.016567	1.788539	-0.914506	2.734983
H	0.256957	0.032823	0.943812	0.532362	2.138769
H	0.156186	-0.001396	0.023726	-0.937694	2.474409
C	-0.558382	-1.360338	-1.200188	-0.708399	-0.242847
H	0.309293	0.060609	-1.540480	-1.266381	0.628027
C	-0.616894	0.288755	-1.210683	-1.386260	-1.587561
H	0.128441	-0.090723	-1.991221	-1.000460	-2.268391
H	0.095448	-0.082083	-0.254589	-1.259032	-2.129166
H	0.151936	-0.023782	-1.378348	-2.463355	-1.484635
C	-1.335143	0.689297	-1.175162	0.714613	-0.166832
C	0.522386	-0.322531	-1.359147	1.432416	1.051478
C	0.037937	-0.382434	-0.907765	1.521319	-1.310488
C	-0.145525	0.237499	-1.302488	2.814544	1.113961
H	0.141746	0.019286	-1.549025	0.866363	1.960827
C	-0.218404	0.244458	-0.851965	2.910626	-1.236604
H	0.155655	0.035981	-0.751476	1.039224	-2.272199
C	-0.214423	-0.431740	-1.050923	3.584885	-0.031433
H	0.127912	0.001262	-1.456717	3.306459	2.073537
H	0.132933	-0.003334	-0.648182	3.476898	-2.144647
H	0.131091	0.012146	-1.008599	4.669310	0.020377
H	-0.172914	-0.413373	5.447963	-0.452530	0.703450
H	0.346496	-0.356570	1.263361	-0.229455	-0.837029
B	1.079463	1.160802	-3.933601	-0.830659	0.022093
C	-0.808361	-0.319008	-4.054764	-0.619935	1.593663
H	0.203165	0.012633	-3.737567	0.373550	1.926788
H	0.146051	-0.035436	-5.124803	-0.710970	1.848097
H	0.153565	-0.013770	-3.528925	-1.379192	2.187060
C	-0.816527	-0.313160	-4.065731	-2.321167	-0.521761
H	0.144133	-0.009071	-3.861477	-2.432368	-1.592359
H	0.170164	-0.005766	-3.437754	-3.035220	0.026728
H	0.147269	-0.051716	-5.110045	-2.637162	-0.358075
C	-0.779830	-0.329095	-4.233691	0.389190	-0.940791
H	0.170962	0.006871	-3.767315	0.282593	-1.928242
H	0.138815	-0.032731	-5.323808	0.428801	-1.109538
H	0.207052	0.005585	-3.925578	1.356353	-0.526585

VIb

Electronic energy: -1269.749396

Electronic energy + zero-point energy: -1269.318616

Electronic energy + thermal energy correction: -1269.280338

Electronic energy + thermal enthalpy correction: -1269.279156

Electronic energy + thermal free energy correction: -1269.393891

Table S17. Cartesian coordinates, Mulliken and APT charges of all atoms at **VIb** in the reaction of styrene with TMDSO.

Symbol	X	Y	Z	Mulliken	APT
Si	1.034809	2.238058	1.277681	-1.130450	-0.214605
O	-0.589303	-1.618128	2.978998	-1.344320	0.155163
Si	0.922012	1.891255	4.106315	-0.305455	0.718106
C	-0.767056	-0.423626	4.997687	0.586794	-0.687305
H	0.195828	0.003699	5.372753	-0.127089	-1.429298
H	0.169296	0.016752	4.316751	1.276773	-1.199285
H	0.179567	-0.010391	5.849180	1.166333	-0.309053
C	-0.815134	-0.422399	3.371281	0.998851	1.863123
H	0.198761	0.009874	2.880169	0.539206	2.727603
H	0.160003	-0.007192	4.146241	1.683951	2.229338
H	0.194633	0.007576	2.611547	1.590902	1.337575
C	-0.907992	-0.431151	1.458215	0.084662	-1.651282
H	0.306277	0.012107	1.499397	1.112196	-1.264537
H	0.149023	-0.025659	2.394747	-0.123734	-2.182830
H	0.182422	0.020498	0.628293	0.055170	-2.362592
C	-0.889694	-0.443141	0.868143	-2.928824	-0.639776
H	0.141071	0.015022	0.480814	-3.015459	-1.660405
H	0.165110	-0.045679	1.774289	-3.538255	-0.554882
H	0.146966	0.005390	0.104694	-3.330751	0.035818
C	-0.837810	-1.287558	-1.289107	-0.727250	-0.396122
H	0.193427	0.058529	-1.339532	-1.279237	0.540960
C	-0.603535	0.283813	-1.772691	-1.421498	-1.643149
H	0.124521	-0.103450	-2.758960	-1.070252	-1.999132
H	0.105529	-0.082343	-1.083196	-1.286441	-2.495539
H	0.163528	-0.028523	-1.860780	-2.500544	-1.477987
C	-1.200363	0.690415	-1.283668	0.696868	-0.324413
C	0.236369	-0.350853	-0.943952	1.406093	0.866592
C	0.213698	-0.370986	-1.555767	1.514400	-1.460648
C	0.030750	0.244458	-0.868349	2.790422	0.905472
H	0.090311	0.027502	-0.728298	0.835951	1.767096
C	-0.370793	0.247338	-1.480211	2.900674	-1.408635
H	0.146512	0.027697	-1.830371	1.037931	-2.399317
C	-0.131884	-0.438907	-1.132483	3.567402	-0.230086

H	0.136173	0.000502	-0.598170	3.277196	1.841878
H	0.127814	-0.004577	-1.700578	3.474157	-2.308294
H	0.129065	0.012084	-1.074827	4.651769	-0.193642
H	-0.161134	-0.416716	5.139269	-1.074311	1.477671
H	0.218240	-0.360271	0.876779	-0.616895	1.119171
B	1.059756	1.077970	-3.812050	-0.880671	0.844128
C	-0.785653	-0.305543	-4.069507	-2.384911	0.396918
H	0.179855	-0.004309	-3.259137	-3.063463	0.692202
H	0.151781	-0.044789	-4.973511	-2.733586	0.924600
H	0.141138	-0.009987	-4.254829	-2.512012	-0.675519
C	-0.781702	-0.321705	-4.454924	0.306574	0.019547
H	0.195880	0.009896	-4.059081	1.291971	0.290934
H	0.173776	0.010712	-4.340858	0.186388	-1.065177
H	0.139818	-0.025902	-5.539816	0.311996	0.222238
C	-0.779042	-0.312432	-3.346453	-0.613181	2.337123
H	0.148900	-0.015400	-2.691623	-1.389895	2.751394
H	0.215718	0.026230	-2.863665	0.362232	2.460357
H	0.152760	-0.025760	-4.254654	-0.594066	2.963557

VIc

Electronic energy: -1269.746896

Electronic energy + zero-point energy: -1269.315288

Electronic energy + thermal energy correction: -1269.274063

Electronic energy + thermal enthalpy correction: -1269.272882

Electronic energy + thermal free energy correction: -1269.396994

Table S18. Cartesian coordinates, Mulliken and APT charges of all atoms at **VIc** in the reaction of styrene with TMDSO.

Symbol	X	Y	Z	Mulliken	APT
Si	1.140065	2.364758	1.434965	-0.787442	0.031049
O	-0.494165	-1.692899	3.129800	-1.204367	0.169568
Si	0.605400	1.956122	4.586101	-0.484307	0.250790
C	-0.698174	-0.433054	5.213863	0.054771	-1.445710
H	0.198083	0.006422	5.185345	-0.780342	-2.154425
H	0.171361	0.019986	4.593895	0.860568	-1.854694
H	0.177540	-0.011298	6.248042	0.416304	-1.384865
C	-0.758476	-0.435367	4.583690	1.033638	1.375776

H	0.197772	0.008154	4.353114	0.766678	2.412694
H	0.160012	-0.004605	5.561369	1.531701	1.361739
H	0.186868	0.010950	3.830128	1.759877	1.047107
C	-0.755827	-0.443040	1.093595	-0.182722	1.789447
H	0.142829	-0.030484	2.014453	-0.187815	2.383473
H	0.244022	0.030311	0.690413	0.835875	1.770099
H	0.164934	-0.004226	0.353859	-0.815781	2.292260
C	-0.959755	-0.439411	1.593638	0.560770	-1.287133
H	0.244435	-0.025056	2.286229	1.327018	-0.910850
H	0.187996	-0.012342	2.038214	0.145333	-2.199957
H	0.168342	0.049742	0.654002	1.059082	-1.537993
C	-0.934250	-1.332067	-1.167782	-0.647594	-0.333656
H	0.314941	0.057964	-1.263391	-1.293495	0.539193
C	-0.631964	0.281917	-1.190659	-1.302647	-1.692592
H	0.123088	-0.104370	-2.159388	-1.206999	-2.216939
H	0.151949	-0.066326	-0.434715	-0.878967	-2.376797
H	0.129491	-0.018542	-0.972843	-2.372379	-1.610406
C	-0.589066	0.731756	-1.609896	0.699586	-0.194949
C	0.207546	-0.348918	-1.829268	1.316617	1.074139
C	0.187216	-0.412837	-1.848593	1.540877	-1.322313
C	-0.353768	0.261969	-2.245494	2.631552	1.198629
H	0.151718	0.019718	-1.666777	0.721782	1.970739
C	-0.337493	0.272510	-2.262014	2.862158	-1.185427
H	0.169710	0.027689	-1.722774	1.130912	-2.322004
C	-0.188347	-0.467092	-2.469647	3.435270	0.070772
H	0.123604	-0.000517	-2.401267	3.044060	2.194819
H	0.122221	-0.004834	-2.435124	3.454308	-2.083534
H	0.124268	0.011389	-2.798131	4.465848	0.172091
H	-0.158096	-0.422698	5.580650	-1.455274	0.800111
H	-0.049334	-0.442496	0.990305	-2.146787	-0.354463
B	0.941682	1.056615	-3.769267	-1.655163	0.291234
C	-0.778005	-0.319535	-4.534956	-0.558828	-0.552559
H	0.176923	0.007615	-4.210214	-0.516374	-1.599805
H	0.139176	-0.024586	-5.604962	-0.830013	-0.564169
H	0.213542	0.009168	-4.452298	0.450301	-0.132264
C	-0.774297	-0.311890	-3.702070	-1.505725	1.870270
H	0.148572	-0.025725	-4.661329	-1.875271	2.271489
H	0.157132	-0.013751	-2.913505	-2.104747	2.342898
H	0.201812	0.019152	-3.605609	-0.465735	2.199654
C	-0.786438	-0.306685	-3.491980	-3.090726	-0.332512
H	0.174837	-0.002966	-2.604646	-3.576885	0.091419
H	0.152136	-0.038451	-4.351797	-3.731951	-0.074029
H	0.146232	-0.007842	-3.402795	-3.097584	-1.424156

VIIa

Electronic energy: -1269.755694

Electronic energy + zero-point energy: -1269.326112

Electronic energy + thermal energy correction: -1269.285395

Electronic energy + thermal enthalpy correction: -1269.284213

Electronic energy + thermal free energy correction: -1269.408013

Table S19. Cartesian coordinates, Mulliken and APT charges of all atoms at **VIIa** in the reaction of styrene with TMDSO.

Symbol	X	Y	Z	Mulliken	APT
Si	0.939876	2.208919	-1.654549	-0.986068	-0.517505
O	-0.530598	-1.603848	-3.454910	-0.744148	-0.274470
Si	0.643174	1.893586	-4.232492	0.479849	0.449718
C	-0.751699	-0.416905	-4.201120	0.368241	2.339114
H	0.188097	-0.006481	-4.608653	-0.590504	2.679724
H	0.168266	0.009157	-3.170749	0.442588	2.705232
H	0.167398	-0.020493	-4.789503	1.173335	2.797865
C	-0.752155	-0.418094	-3.536019	2.174244	-0.024811
H	0.193147	-0.002151	-3.585162	2.329799	-1.108756
H	0.152010	-0.014790	-4.087188	2.987918	0.464052
H	0.230528	0.006754	-2.481946	2.248927	0.271598
C	-0.887330	-0.423146	-1.918725	-2.868492	-0.713220
H	0.146258	-0.046115	-2.854164	-3.030645	-1.259207
H	0.161448	-0.022476	-1.096683	-3.371274	-1.235546
H	0.165525	-0.015437	-2.035169	-3.338032	0.272029
C	-0.945490	-0.423257	-1.640473	-0.021985	-2.167016
H	0.143726	-0.028510	-2.639614	-0.051032	-2.614606
H	0.243303	0.004940	-1.368289	1.029060	-2.006112
H	0.136896	-0.021862	-0.918275	-0.438376	-2.879947
C	-0.656099	-0.870170	0.486997	-1.225935	-0.464132
H	0.054066	-0.051661	0.759925	-1.719256	-1.408438
C	-0.423873	0.169253	0.764848	-2.124961	0.736931
H	0.126425	-0.087570	1.807950	-2.079549	1.086680
H	0.135691	-0.033059	0.129785	-1.855009	1.595760
H	0.141689	-0.036617	0.543062	-3.170417	0.496260
C	-1.595687	0.451726	1.085944	0.110879	-0.386918
C	0.761042	-0.207753	1.422930	0.846889	-1.548186
C	0.039463	-0.240938	1.266819	0.780426	0.844065

C	-0.067792	0.100316	1.846008	2.167832	-1.489043
H	0.158637	0.024687	1.323240	0.360220	-2.516660
C	-0.086325	0.100280	1.696219	2.106877	0.905173
H	0.168680	0.046240	1.045194	0.253055	1.768400
C	-0.206108	-0.257685	1.979351	2.820554	-0.257495
H	0.148882	0.011016	2.082695	2.695734	-2.410832
H	0.147343	0.012981	1.809989	2.584464	1.876630
H	0.145456	0.021106	2.312859	3.853809	-0.209896
H	-0.183178	-0.438052	-5.679276	0.469946	0.050340
H	0.292914	-0.381617	-1.411621	-0.309995	0.817348
B	0.818233	0.851285	4.469615	-0.576173	0.537903
C	-0.773256	-0.297432	5.007197	0.722636	-0.172383
H	0.271005	0.076424	4.116611	1.333455	-0.395222
H	0.137068	-0.022474	5.644817	1.342214	0.471066
H	0.144539	-0.024421	5.515049	0.544943	-1.127336
C	-0.783695	-0.297167	4.170509	-1.871626	-0.312289
H	0.146336	-0.015677	4.078020	-2.792153	0.275948
H	0.293592	0.067418	3.196390	-1.714999	-0.804239
H	0.142365	-0.025966	4.899704	-2.027553	-1.117258
C	-0.808613	-0.296241	4.287553	-0.569376	2.108928
H	0.182343	0.004006	3.523966	-1.263571	2.475812
H	0.134766	-0.015043	5.249954	-0.901784	2.536056
H	0.181712	0.003016	4.088627	0.428591	2.516215

VIIb

Electronic energy: -1269.754205

Electronic energy + zero-point energy: -1269.322847

Electronic energy + thermal energy correction: -1269.284027

Electronic energy + thermal enthalpy correction: -1269.282846

Electronic energy + thermal free energy correction: -1269.399618

Table S20. Cartesian coordinates, Mulliken and APT charges of all atoms at **VIIb** in the reaction of styrene with TMDSO.

Symbol	X	Y	Z	Mulliken	APT
Si	1.460324	2.229345	1.598235	1.106054	-0.241085
O	-0.563001	-1.620403	3.422224	0.888402	-0.289265
Si	0.796721	1.894846	4.392867	-0.405136	-0.314049

C	-0.754574	-0.419702	4.934662	-0.948367	1.418887
H	0.191776	-0.004966	5.379597	-0.110840	1.968743
H	0.163637	0.006804	4.075769	-1.308221	1.997726
H	0.175333	-0.018386	5.676121	-1.756250	1.366740
C	-0.800719	-0.419269	3.619986	-1.918522	-1.147214
H	0.192005	0.008840	3.335864	-1.696823	-2.181819
H	0.156387	-0.019339	4.319875	-2.764230	-1.152896
H	0.205321	0.004237	2.708894	-2.230654	-0.621868
C	-1.009232	-0.398302	1.517143	0.426941	1.547739
H	0.276538	-0.009300	1.547745	-0.672007	1.510343
H	0.164573	-0.025050	2.397497	0.762649	2.107425
H	0.121549	-0.014047	0.612879	0.697120	2.102586
C	-0.936969	-0.425797	1.760389	2.994123	-0.486530
H	0.138193	-0.013328	1.466051	3.520216	0.431120
H	0.167863	-0.044197	2.796547	3.256748	-0.716459
H	0.134181	-0.022857	1.107057	3.356290	-1.290250
C	-0.718496	-0.870554	-0.562899	1.241771	-0.385605
H	0.094324	-0.033273	-0.654452	1.503962	-1.449582
C	-0.594430	0.166644	-1.145873	2.339636	0.497620
H	0.149519	-0.095833	-2.239819	2.272853	0.621424
H	0.147741	-0.037082	-0.714112	2.328851	1.509797
H	0.157618	-0.033352	-0.926974	3.326091	0.075489
C	-1.196019	0.457067	-1.092805	-0.106564	-0.149907
C	0.425543	-0.220388	-0.934612	-1.132489	-1.114277
C	-0.140871	-0.270837	-1.702036	-0.489761	1.065754
C	0.069472	0.114456	-1.296247	-2.448767	-0.855204
H	0.123484	0.034260	-0.497941	-0.875521	-2.076362
C	-0.094061	0.099714	-2.070102	-1.810413	1.323070
H	0.146619	0.045101	-1.869360	0.261089	1.834893
C	-0.302113	-0.255005	-1.861265	-2.808083	0.373267
H	0.162109	0.012308	-1.140814	-3.205402	-1.621887
H	0.153396	0.009471	-2.530816	-2.057575	2.278237
H	0.138355	0.020149	-2.148784	-3.836906	0.573390
H	-0.160544	-0.442674	5.657015	-0.094161	-1.060570
H	0.056802	-0.409043	1.474640	0.188581	-1.424590
B	0.843967	0.877292	-4.593805	0.312542	-0.204204
C	-0.785183	-0.301293	-4.169365	1.394400	-1.274188
H	0.329336	0.074150	-3.072842	1.342576	-1.371035
H	0.142081	-0.025148	-4.574262	1.193737	-2.274185
H	0.141437	-0.020667	-4.407166	2.426603	-0.989144
C	-0.814405	-0.302719	-4.898561	0.735049	1.289302
H	0.188906	0.010895	-4.714429	-0.067183	2.012712
H	0.179763	0.003453	-4.375787	1.640863	1.615853
H	0.131932	-0.015564	-5.979037	0.958786	1.333131
C	-0.816356	-0.295611	-4.830639	-1.171715	-0.685025

H	0.257238	0.051552	-3.884743	-1.562833	-1.088858
H	0.162337	-0.020080	-5.164521	-1.863114	0.096611
H	0.140596	-0.016516	-5.550540	-1.201497	-1.515828

VIIc

Electronic energy: -1269.751092

Electronic energy + zero-point energy: -1269.318828

Electronic energy + thermal energy correction: -1269.276884

Electronic energy + thermal enthalpy correction: -1269.275702

Electronic energy + thermal free energy correction: -1269.401530

Table S21. Cartesian coordinates, Mulliken and APT charges of all atoms at **VIIc** in the reaction of styrene with TMDSO.

Symbol	X	Y	Z	Mulliken	APT
Si	1.463859	2.333767	1.370414	-0.797103	-0.000438
O	-0.550434	-1.684928	3.163659	-1.158540	0.196754
Si	0.585335	1.945707	4.609054	-0.457641	0.327758
C	-0.725218	-0.431653	5.345741	0.075491	-1.335575
H	0.194754	-0.000901	5.321306	-0.752781	-2.053185
H	0.174882	0.012489	4.780516	0.908452	-1.769689
H	0.171084	-0.022721	6.389475	0.394816	-1.217543
C	-0.754799	-0.428638	4.609111	1.083687	1.434063
H	0.191344	-0.000956	4.357134	0.836012	2.471086
H	0.153131	-0.017672	5.592662	1.571549	1.429068
H	0.183738	0.003169	3.867238	1.810701	1.080451
C	-0.787568	-0.408556	1.177913	-0.139591	1.784480
H	0.161719	-0.032514	2.108374	-0.240558	2.350655
H	0.220099	-0.002255	0.890063	0.919835	1.772220
H	0.125830	-0.028702	0.387847	-0.680177	2.321339
C	-0.929587	-0.387848	1.754845	0.484576	-1.373748
H	0.250045	-0.032319	2.415591	1.256021	-0.954548
H	0.190279	-0.024336	2.314395	-0.001543	-2.182946
H	0.125521	-0.008561	0.884024	0.992272	-1.798688
C	-0.861274	-0.880751	-0.758401	-0.710915	-0.368956
H	0.290547	-0.049831	-1.117238	-1.371804	0.436154
C	-0.662758	0.142443	-0.963215	-1.372083	-1.730027
H	0.151373	-0.097030	-2.005990	-1.334187	-2.089383

H	0.158419	-0.027322	-0.343485	-0.900336	-2.507223
H	0.099946	-0.021464	-0.658686	-2.424149	-1.690140
C	-0.939622	0.439582	-1.402055	0.609709	-0.240446
C	0.325104	-0.224941	-1.777920	1.128560	1.019706
C	0.185668	-0.232995	-1.674314	1.438226	-1.351010
C	-0.345044	0.112508	-2.380171	2.372334	1.161124
H	0.188760	0.034459	-1.601149	0.520756	1.904278
C	-0.351269	0.113863	-2.271674	2.689908	-1.212624
H	0.169260	0.037606	-1.430925	1.083883	-2.349460
C	-0.066651	-0.249847	-2.635620	3.173080	0.043375
H	0.132293	0.011549	-2.660917	2.719372	2.153968
H	0.129290	0.006966	-2.466389	3.287870	-2.101067
H	0.148501	0.016776	-3.109169	4.145337	0.151878
H	-0.165187	-0.446379	5.602474	-1.411316	0.924160
H	-0.134194	-0.479086	1.180443	-2.258610	-0.277860
B	0.921819	0.866465	-4.150837	-1.449646	0.327547
C	-0.824202	-0.293464	-4.639443	-0.308215	-0.641519
H	0.165417	0.002345	-4.328274	-0.447896	-1.683250
H	0.130984	-0.009152	-5.743646	-0.350156	-0.631309
H	0.227400	0.021448	-4.352753	0.699069	-0.318816
C	-0.794585	-0.298227	-4.061421	-1.214620	1.889666
H	0.151216	-0.012173	-4.828220	-1.828733	2.387518
H	0.200409	0.021150	-3.097645	-1.576340	2.275014
H	0.187423	-0.006110	-4.188402	-0.174193	2.204894
C	-0.790255	-0.283073	-3.858490	-2.900642	-0.228574
H	0.241772	0.049968	-2.786697	-2.933569	-0.476917
H	0.146742	-0.026163	-4.034014	-3.699892	0.501992
H	0.138688	-0.021690	-4.394900	-3.133787	-1.156526

VIIIa

Electronic energy: -1269.757491

Electronic energy + zero-point energy: -1269.327456

Electronic energy + thermal energy correction: -1269.288965

Electronic energy + thermal enthalpy correction: -1269.287783

Electronic energy + thermal free energy correction: -1269.404282

Table S22. Cartesian coordinates, Mulliken and APT charges of all atoms at **VIIIa** in the reaction of styrene with TMDSO.

Symbol	X	Y	Z	Mulliken	APT
Si	-0.266118	2.164182	-0.578681	-1.264232	-0.388059
O	-0.463605	-1.627817	-2.200718	-0.456800	-0.806772
Si	0.391711	1.897498	-3.536502	-0.203930	0.073496
C	-0.780670	-0.416362	-3.159797	0.356100	1.842604
H	0.208258	0.003801	-2.586670	-0.407994	2.381337
H	0.221111	0.004213	-2.553578	1.270167	1.833877
H	0.145390	-0.022872	-4.080960	0.555596	2.405192
C	-0.642394	-0.411224	-4.642273	1.099676	-0.734885
H	0.189956	-0.003447	-4.890433	0.809494	-1.762145
H	0.181791	-0.026846	-5.580578	1.220343	-0.178211
H	0.172398	0.010469	-4.138554	2.072160	-0.774700
C	-0.921323	-0.416181	-1.481688	-2.923937	-0.030574
H	0.149727	-0.049522	-2.274648	-3.063009	-0.773989
H	0.152434	-0.027492	-0.813194	-3.792663	-0.053198
H	0.187752	-0.021138	-1.968990	-2.905639	0.954967
C	-0.627692	-0.398476	0.022425	-1.070336	-2.185621
H	0.162880	-0.019607	-0.841525	-1.030743	-2.856070
H	0.264986	-0.003823	0.591681	-0.140863	-2.315283
H	0.179221	-0.029284	0.677270	-1.897685	-2.485425
C	-0.507534	-0.777908	1.235640	-2.032269	0.354704
H	0.033774	-0.073322	1.459042	-2.921512	-0.253678
C	-0.286592	0.151234	0.979674	-2.435373	1.807581
H	0.166992	-0.092710	1.907800	-2.597488	2.379207
H	0.134206	-0.030063	0.404197	-1.662712	2.342361
H	0.134390	-0.032183	0.390409	-3.356873	1.859001
C	-0.567386	0.428410	2.332597	-1.054458	0.209122
C	0.784295	-0.193971	3.151869	-1.022304	-0.940629
C	-0.649216	-0.220862	2.598540	-0.076524	1.190515
C	0.020260	0.091211	4.171520	-0.089899	-1.095542
H	0.146107	0.024980	2.977934	-1.759367	-1.722426
C	-0.281037	0.081038	3.623018	0.857447	1.039247
H	0.142720	0.044405	1.989704	-0.050453	2.090627
C	-0.151424	-0.224534	4.422733	0.860449	-0.101568
H	0.145670	0.008766	4.778947	-0.104963	-1.998366
H	0.124275	0.014145	3.791724	1.595109	1.821600
H	0.143204	0.018247	5.217887	1.591776	-0.221383
H	-0.155234	-0.437621	-4.383834	-1.439986	0.196912
H	0.724212	-0.383910	-0.375465	-0.167933	0.642318
B	0.537586	0.848010	0.193732	2.772245	-0.028831
C	-0.752259	-0.292483	1.359469	2.561246	-1.067635
H	0.135110	-0.027239	1.073212	2.714616	-2.114625
H	0.171661	-0.005293	2.249167	3.162650	-0.839507
H	0.172397	0.041591	1.693835	1.515511	-0.966624
C	-0.824045	-0.286528	0.502395	2.857398	1.520763

H	0.126907	-0.022328	-0.050164	3.676104	2.002351
H	0.283390	0.038073	0.124754	1.927436	1.973697
H	0.201217	-0.019258	1.564647	2.939885	1.775322
C	-0.909121	-0.343707	-1.298109	2.820067	-0.527372
H	0.208404	-0.026076	-2.026877	3.158058	0.219689
H	0.137087	-0.015250	-1.422730	3.411859	-1.444013
H	0.504170	0.109060	-1.562443	1.782872	-0.800860

VIIIb

Electronic energy: -1269.756405

Electronic energy + zero-point energy: -1269.325621

Electronic energy + thermal energy correction: -1269.287358

Electronic energy + thermal enthalpy correction: -1269.286177

Electronic energy + thermal free energy correction: -1269.401230

Table S23. Cartesian coordinates, Mulliken and APT charges of all atoms at **VIIIb** in the reaction of styrene with TMDSO.

Symbol	X	Y	Z	Mulliken	APT
Si	-0.478392	2.151194	0.227515	-0.925054	0.789430
O	-0.419081	-1.605571	1.971449	-1.226049	0.194878
Si	0.701000	1.854069	2.653725	-1.251158	-1.272393
C	-0.813281	-0.407759	2.008183	0.117053	-2.409958
H	0.208537	0.008827	0.916230	0.076612	-2.505429
H	0.213198	0.005294	2.264754	1.101410	-1.997962
H	0.150130	-0.022592	2.445802	0.042120	-3.413849
C	-0.715603	-0.404348	4.528438	-1.034917	-1.135519
H	0.189057	-0.004900	4.963048	-1.806459	-0.489988
H	0.176062	-0.023233	5.010772	-1.098986	-2.119344
H	0.163644	0.003790	4.769711	-0.058388	-0.698497
C	-0.656516	-0.381056	-0.360109	-2.431199	-0.239877
H	0.214352	-0.012871	-0.290644	-2.174909	-1.306625
H	0.141579	-0.019289	0.322475	-3.272668	-0.073074
H	0.196406	-0.020786	-1.387932	-2.757454	-0.056546
C	-0.526008	-0.410341	0.873737	-1.140583	2.580957
H	0.169151	-0.026613	0.961490	-2.213360	2.802492
H	0.188900	-0.033198	1.882971	-0.720610	2.648825
H	0.167370	-0.025804	0.238349	-0.685774	3.348560

C	-1.207223	-0.757695	-1.708083	-0.413382	1.431001
H	0.123425	-0.040379	-1.508021	0.492422	2.022653
C	-0.741380	0.151113	-2.334239	-1.479058	2.329354
H	0.169286	-0.106192	-3.387510	-1.266595	2.577469
H	0.121421	-0.028627	-2.311450	-2.476943	1.869680
H	0.127185	-0.030543	-1.783535	-1.560833	3.272596
C	0.158766	0.396222	-2.526733	-0.043145	0.257128
C	0.039069	-0.180944	-2.334014	1.197273	-0.390458
C	0.329745	-0.209484	-3.477502	-0.906947	-0.322520
C	-0.067121	0.088851	-3.026329	1.542588	-1.545220
H	0.173412	0.032690	-1.618704	1.897518	0.034924
C	-0.199422	0.091745	-4.174683	-0.563304	-1.480052
H	0.113100	0.030731	-3.674052	-1.869875	0.142622
C	-0.033256	-0.227921	-3.955391	0.662277	-2.107081
H	0.133873	0.014067	-2.840498	2.508829	-2.010844
H	0.128313	0.003346	-4.897836	-1.263177	-1.894809
H	0.134059	0.013267	-4.498617	0.929581	-3.009861
H	-0.133387	-0.434426	2.432792	-2.551108	-1.992755
H	0.099987	-0.438498	0.290660	0.441041	0.147463
B	0.931332	0.917939	1.376335	2.651411	0.798003
C	-0.813926	-0.300900	0.547626	2.649943	2.144376
H	0.143995	-0.009394	-0.536135	2.736456	2.006920
H	0.182445	0.015486	0.755515	1.775889	2.773125
H	0.143291	-0.019852	0.867672	3.537036	2.718262
C	-0.858242	-0.290273	0.798496	3.350462	-0.500686
H	0.137181	-0.028962	1.563485	3.901068	-1.063334
H	0.352686	0.043796	0.438577	2.554142	-1.170086
H	0.153906	-0.029250	-0.053757	4.014905	-0.310279
C	-0.812335	-0.340668	2.829400	2.037025	0.805367
H	0.172307	-0.030519	3.469198	2.357216	-0.026227
H	0.124601	-0.021747	3.356231	2.222453	1.751606
H	0.402404	0.102210	2.715559	0.942206	0.733897

IX^{H}a

Electronic energy: -1269.752297

Electronic energy + zero-point energy: -1269.321297

Electronic energy + thermal energy correction: -1269.284072

Electronic energy + thermal enthalpy correction: -1269.282890

Electronic energy + thermal free energy correction: -1269.393767

Table S24. Cartesian coordinates, Mulliken and APT charges of all atoms at $\mathbf{IX^{H}a}$ in the reaction of styrene with TMDSO.

Symbol	X	Y	Z	Mulliken	APT
Si	0.370172	2.313783	-0.511758	-1.062161	-0.356339
O	-0.455225	-1.605363	-2.150034	-0.405482	-0.828198
Si	0.573416	1.898353	-3.587336	-0.322960	-0.083480
C	-0.721351	-0.418581	-3.453981	0.072313	1.765416
H	0.186206	0.001169	-2.914150	-0.716045	2.303143
H	0.161883	0.009143	-2.911239	1.010042	1.929166
H	0.167907	-0.019573	-4.449346	0.167761	2.218566
C	-0.754715	-0.414715	-4.713025	0.962547	-0.891456
H	0.181802	-0.000695	-4.767508	0.793473	-1.973046
H	0.173485	-0.024860	-5.730114	0.891568	-0.484831
H	0.174736	0.017601	-4.346354	1.981400	-0.729211
C	-0.773522	-0.416439	-1.369811	-2.704219	0.213690
H	0.162231	-0.050105	-2.050550	-3.018659	-0.586886
H	0.149865	-0.031719	-0.664780	-3.518631	0.417670
H	0.203788	-0.024240	-1.986957	-2.570131	1.112578
C	-0.827441	-0.426420	0.125544	-1.206575	-2.140533
H	0.151902	-0.011592	-0.693344	-1.025721	-2.843479
H	0.245427	0.005601	0.931107	-0.494032	-2.348450
H	0.153736	-0.025843	0.524790	-2.215139	-2.315325
C	-0.552753	-0.742477	1.233287	-1.653178	0.622753
H	0.053432	-0.072170	1.394624	-2.676149	0.250297
C	-0.302131	0.124484	0.905127	-1.691443	2.116017
H	0.159684	-0.089162	1.788014	-1.887241	2.745421
H	0.181016	-0.020639	0.474184	-0.735688	2.452943
H	0.136785	-0.027214	0.164042	-2.467498	2.333354
C	-0.550572	0.357442	2.443533	-0.853905	0.312914
C	0.851128	-0.174208	3.176378	-1.079521	-0.871209
C	-0.608818	-0.203605	2.917191	0.168305	1.154738
C	-0.031482	0.077102	4.283248	-0.311656	-1.210891
H	0.153867	0.027608	2.853519	-1.876721	-1.537754
C	-0.424093	0.069274	4.025984	0.944997	0.817258
H	0.145006	0.042959	2.400414	0.368431	2.088388
C	-0.125388	-0.200401	4.716986	0.718174	-0.370195
H	0.141835	0.009912	4.813209	-0.514953	-2.139202
H	0.145712	0.011197	4.346483	1.738857	1.489445
H	0.147769	0.016516	5.577809	1.325723	-0.637187
H	-0.118000	-0.436525	-4.341161	-1.620017	-0.168834
H	-0.253718	-0.613790	-0.276126	0.324493	0.322191
B	1.580592	1.154406	-0.051224	2.256625	0.020401
C	-0.924166	-0.291317	1.019491	2.216160	-1.153994

H	0.126940	-0.005469	0.598703	1.874403	-2.107260
H	0.111741	-0.033741	1.359292	3.255580	-1.308308
H	0.199165	0.005003	1.915582	1.626368	-0.932996
C	-0.790691	-0.297134	0.406854	2.603161	1.510020
H	0.132951	-0.050118	0.222661	3.678974	1.670501
H	0.174609	-0.006963	-0.190561	2.075163	2.264216
H	0.205615	-0.018178	1.466101	2.417498	1.712486
C	-1.019956	-0.306738	-1.543027	2.628148	-0.381098
H	0.235779	-0.031180	-2.269996	2.532466	0.434483
H	0.095575	-0.047661	-1.531720	3.693130	-0.673045
H	0.198264	-0.002717	-1.905624	2.053893	-1.238545

IX^{H}b

Electronic energy: -1269.754488

Electronic energy + zero-point energy: -1269.323311

Electronic energy + thermal energy correction: -1269.284380

Electronic energy + thermal enthalpy correction: -1269.283198

Electronic energy + thermal free energy correction: -1269.399811

Table S25. Cartesian coordinates, Mulliken and APT charges of all atoms at **IX^{H}b** in the reaction of styrene with TMDSO.

Symbol	X	Y	Z	Mulliken	APT
Si	-0.134173	2.248755	0.283120	-0.859203	0.814580
O	-0.423486	-1.587156	2.003219	-1.069186	0.196724
Si	0.748401	1.858486	2.708708	-1.144126	-1.256946
C	-0.801020	-0.411713	2.033039	0.120124	-2.495017
H	0.208301	0.004749	0.936492	0.097789	-2.523751
H	0.175103	0.004884	2.333791	1.138222	-2.221218
H	0.155867	-0.020753	2.405524	-0.083626	-3.507212
C	-0.759360	-0.406815	4.574127	-0.873153	-1.105393
H	0.179113	-0.004682	5.016421	-1.614506	-0.430319
H	0.179414	-0.021449	5.069652	-0.959758	-2.080889
H	0.143496	0.006977	4.790391	0.120568	-0.696306
C	-0.627500	-0.391297	-0.291478	-2.305814	-0.307626
H	0.230075	-0.006195	-0.316056	-1.962415	-1.351902
H	0.140196	-0.027961	0.439402	-3.120944	-0.256867
H	0.192125	-0.019466	-1.287065	-2.700077	-0.083723

C	-0.620169	-0.423590	0.867235	-1.214641	2.602051
H	0.163665	-0.023539	0.681981	-2.272156	2.838032
H	0.153649	-0.029040	1.944246	-1.042067	2.687493
H	0.185944	-0.021734	0.340112	-0.615178	3.353095
C	-1.367960	-0.762650	-1.673802	-0.407285	1.429746
H	0.127875	-0.034153	-1.525112	0.505702	2.023274
C	-0.698219	0.147797	-2.274279	-1.494535	2.320538
H	0.165771	-0.106622	-3.346003	-1.335093	2.524822
H	0.130111	-0.030383	-2.180431	-2.496293	1.877597
H	0.135265	-0.027823	-1.757521	-1.531122	3.285132
C	1.151474	0.387480	-2.491169	-0.074141	0.240759
C	0.191763	-0.183916	-2.312392	1.156740	-0.425937
C	-0.393134	-0.208253	-3.423333	-0.963360	-0.329846
C	-0.114121	0.085769	-2.997835	1.470054	-1.593840
H	0.101653	0.046453	-1.611794	1.873279	-0.007110
C	-0.335742	0.085203	-4.115162	-0.651543	-1.499660
H	0.096274	0.032639	-3.608177	-1.921562	0.149697
C	-0.187570	-0.220176	-3.907871	0.565227	-2.147589
H	0.141732	0.015781	-2.820450	2.429888	-2.075556
H	0.132617	0.002924	-4.823984	-1.369577	-1.908050
H	0.136388	0.013155	-4.445894	0.806920	-3.060660
H	-0.085702	-0.428920	2.536983	-2.489786	-1.903874
H	-0.178797	-0.593325	0.351583	0.594033	0.325194
B	1.231286	1.107858	1.147435	2.431896	0.821215
C	-0.795911	-0.312098	0.343108	2.606435	2.179430
H	0.150780	-0.014844	-0.742074	2.694686	2.050904
H	0.146420	0.003090	0.546223	1.818806	2.914508
H	0.131546	-0.031166	0.684129	3.556796	2.625644
C	-0.856210	-0.300439	0.660766	3.195219	-0.491125
H	0.125271	-0.037937	1.417197	3.943736	-0.774848
H	0.273124	0.020531	0.579590	2.507075	-1.341474
H	0.156172	-0.038796	-0.300869	3.713342	-0.387907
C	-0.840463	-0.310931	2.658924	1.960489	0.900659
H	0.274030	-0.023676	3.117346	1.783688	-0.079119
H	0.115494	-0.042389	3.228386	2.774265	1.382169
H	0.249143	0.031356	2.798172	1.057084	1.503569

X^{o}a

Electronic energy: -1269.793257

Electronic energy + zero-point energy: -1269.359945

Electronic energy + thermal energy correction: -1269.321978

Electronic energy + thermal enthalpy correction: -1269.320797

Electronic energy + thermal free energy correction: -1269.434891

Table S26. Cartesian coordinates, Mulliken and APT charges of all atoms at $\mathbf{X^{O}a}$ in the reaction of styrene with TMDSO.

Symbol	X	Y	Z	Mulliken	APT
Si	0.954921	1.501453	0.622543	-0.803159	0.445113
O	-0.589758	-1.479787	-2.743804	0.025164	0.183440
Si	0.016026	1.837093	-2.743085	1.521143	0.809387
C	-0.589839	-0.385734	-2.296692	1.429999	2.645679
H	0.185662	-0.008409	-3.055341	0.859230	3.193262
H	0.152034	-0.001428	-1.333380	0.924037	2.781122
H	0.183202	-0.019560	-2.223428	2.427988	3.096165
C	-0.599427	-0.403821	-1.491547	2.662656	-0.038112
H	0.226676	0.008807	-1.687161	2.705369	-1.116065
H	0.157824	-0.025639	-1.551507	3.682781	0.363364
H	0.184083	-0.006170	-0.465272	2.302082	0.102524
C	-0.931240	-0.448348	-0.540558	-2.114082	1.110809
H	0.341901	0.093108	-1.560559	-1.716172	1.055467
H	0.182624	0.011773	-0.500751	-3.026731	0.503758
H	0.146401	-0.002967	-0.304720	-2.372128	2.149942
C	-1.072476	-0.437056	0.234405	-0.248690	-1.301635
H	0.259210	0.084941	-0.810449	0.077328	-1.340074
H	0.187719	0.005236	0.884289	0.577657	-1.611643
H	0.149381	0.011743	0.356215	-1.072848	-2.015477
C	-0.643581	-0.428324	2.422939	-1.491489	0.507198
H	0.098971	-0.038736	2.440746	-2.350904	-0.179273
C	-0.521290	0.081921	2.760223	-1.989681	1.919149
H	0.179895	-0.049378	3.819988	-2.262904	2.009342
H	0.178811	-0.006670	2.546069	-1.232241	2.683140
H	0.157162	-0.014968	2.158400	-2.870014	2.166048
C	-0.757589	0.197292	3.382574	-0.460433	-0.020152
C	0.292720	-0.129057	3.724628	-0.432975	-1.380385
C	0.190527	-0.141546	3.926844	0.533882	0.804992
C	-0.013077	0.024802	4.573292	0.542355	-1.896638
H	0.161226	0.034855	3.305622	-1.187719	-2.043286
C	-0.118052	0.028960	4.778469	1.511742	0.293028
H	0.138556	0.046553	3.673781	0.549995	1.862032
C	-0.127195	-0.121838	5.108165	1.522535	-1.060774
H	0.162077	0.025612	4.816216	0.537654	-2.956264
H	0.145874	0.023586	5.181559	2.271794	0.957695

H	0.141825	0.029301	5.770974	2.285153	-1.460268
H	-0.161286	-0.415206	-4.068074	2.215005	0.718452
H	0.257864	-0.356463	0.655484	0.370423	1.368827
B	1.361453	1.193835	-3.652203	-0.529475	-0.935143
C	-0.975344	-0.211338	-3.618085	0.493944	-2.226498
H	0.126105	-0.082538	-3.984598	1.505038	-1.976098
H	0.165973	-0.076896	-2.609669	0.616084	-2.656512
H	0.117157	-0.071532	-4.263388	0.124939	-3.039790
C	-0.997316	-0.222485	-5.181223	-0.661867	-0.348024
H	0.118746	-0.075202	-5.862184	-1.120845	-1.083208
H	0.126984	-0.075612	-5.208504	-1.293593	0.554927
H	0.116786	-0.072405	-5.620264	0.311385	-0.074749
C	-0.921649	-0.198125	-3.078723	-2.011451	-1.344548
H	0.126246	-0.066978	-3.147368	-2.727462	-0.509234
H	0.091650	-0.080797	-3.657933	-2.439941	-2.178302
H	0.234846	-0.085856	-2.024023	-1.994842	-1.665158

X^Ha

Electronic energy: -1269.804958

Electronic energy + zero-point energy: -1269.371100

Electronic energy + thermal energy correction: -1269.331992

Electronic energy + thermal enthalpy correction: -1269.330810

Electronic energy + thermal free energy correction: -1269.448705

Table S27. Cartesian coordinates, Mulliken and APT charges of all atoms at **X^Ha** in the reaction of styrene with TMDSO.

Symbol	X	Y	Z	Mulliken	APT
Si	0.642976	2.050717	0.193246	-1.760529	-0.331131
O	-0.370248	-1.646644	-1.369910	-2.031345	0.148843
Si	1.154967	1.962539	-2.929032	-1.458786	0.094503
C	-0.794906	-0.441362	-3.306936	-0.395113	1.585705
H	0.179496	-0.003551	-3.095823	-0.935262	2.515799
H	0.156797	0.070866	-2.707874	0.521271	1.558587
H	0.159077	0.001033	-4.365637	-0.107322	1.588596
C	-0.803022	-0.439086	-3.273483	-0.593265	-1.529280
H	0.179420	-0.007876	-3.108230	-1.266664	-2.378668
H	0.171044	0.009659	-4.313525	-0.246189	-1.563368

H	0.177176	0.073352	-2.627922	0.285559	-1.631832
C	-0.831792	-0.427643	0.946925	-3.443375	-0.727450
H	0.170078	-0.004614	0.465981	-3.899239	-1.600466
H	0.174075	0.009512	2.017232	-3.340055	-0.946645
H	0.168814	-0.003592	0.836322	-4.131204	0.118823
C	-0.633360	-0.465758	0.274290	-0.693856	-1.869686
H	0.146639	-0.001719	-0.357309	-1.128694	-2.654818
H	0.322694	0.074896	-0.068199	0.328925	-1.676299
H	0.183493	0.004611	1.304105	-0.646543	-2.244947
C	-1.278287	-0.397685	1.175881	-1.048518	1.144414
H	0.102821	-0.053768	1.599845	-1.923660	1.660944
C	-0.239314	0.066840	0.284591	-0.283750	2.136880
H	0.150931	-0.056704	0.887603	0.135839	2.952210
H	0.249093	0.061213	-0.253884	0.538232	1.654632
H	0.164911	-0.026752	-0.464156	-0.956895	2.567996
C	0.105677	0.152475	2.326253	-0.227446	0.618423
C	0.402800	-0.114444	3.613762	-0.768023	0.513035
C	-0.331502	-0.132818	2.119845	1.085913	0.170803
C	-0.010306	-0.003426	4.667430	-0.025927	-0.021535
H	0.139231	0.021169	3.790091	-1.786570	0.856863
C	-0.073898	0.008184	3.170231	1.829101	-0.359788
H	-0.315500	0.121341	1.122970	1.521661	0.219934
C	0.147439	-0.098538	4.449927	1.278723	-0.459382
H	0.141939	0.014415	5.658542	-0.468438	-0.092313
H	0.140898	0.041151	2.978993	2.842119	-0.704898
H	0.144757	0.021189	5.267692	1.861170	-0.876253
H	-0.193697	-0.415487	-3.789455	-2.680522	0.165020
H	-1.935058	-0.442548	-1.253003	1.249880	-0.082269
B	2.270285	0.691263	-1.443989	2.486291	-0.122310
C	-0.845818	-0.140642	-0.612994	3.114681	-1.399775
H	0.101730	-0.084585	-0.940079	2.690311	-2.364727
H	0.131812	-0.104584	-0.776499	4.204440	-1.470569
H	0.231283	-0.093052	0.480204	2.971040	-1.352905
C	-0.848001	-0.145880	-0.942230	3.151221	1.300828
H	0.139476	-0.105641	-1.123445	4.240183	1.301005
H	0.114900	-0.083634	-1.483555	2.749010	2.175233
H	0.201523	-0.091659	0.132624	3.020321	1.515110
C	-0.932782	-0.152813	-3.052567	2.785879	-0.326565
H	0.117667	-0.083186	-3.691317	2.343860	0.456640
H	0.141515	-0.105627	-3.241135	3.873696	-0.298943
H	0.110057	-0.081109	-3.446488	2.431964	-1.293634

$X^{O}b$

Electronic energy: -1269.789431

Electronic energy + zero-point energy: -1269.357621

Electronic energy + thermal energy correction: -1269.321265

Electronic energy + thermal enthalpy correction: -1269.320083

Electronic energy + thermal free energy correction: -1269.4429922

Table S28. Cartesian coordinates, Mulliken and APT charges of all atoms at $\mathbf{X^{O}b}$ in the reaction of styrene with TMDSO.

Symbol	X	Y	Z	Mulliken	APT
Si	-0.081562	1.494905	-0.529264	-0.783860	0.514741
O	-0.559585	-1.473089	2.798572	-0.061015	0.133835
Si	0.118699	1.849398	3.130239	-0.519522	-1.380645
C	-0.618459	-0.386962	3.137009	-2.413267	-1.434057
H	0.191174	-0.010172	2.184418	-2.809404	-1.059923
H	0.177444	-0.020546	3.288008	-2.796243	-2.451091
H	0.184399	-0.006172	3.931256	-2.812652	-0.793194
C	-0.595178	-0.403218	1.869541	0.069757	-2.666283
H	0.213933	0.013844	1.802132	1.162988	-2.667942
H	0.167452	-0.027288	2.156247	-0.261927	-3.673220
H	0.180961	-0.009744	0.867879	-0.322307	-2.451865
C	-0.696834	-0.398941	-1.064133	-1.943119	-0.882616
H	0.196575	0.008373	-1.680449	-1.414665	-1.618872
H	0.180777	0.011921	-0.192154	-2.355160	-1.402988
H	0.173175	-0.002300	-1.648158	-2.787672	-0.494785
C	-0.869614	-0.442066	0.595950	-1.688161	1.714442
H	0.132075	-0.012004	0.287825	-2.735321	1.828134
H	0.271913	0.083743	1.624308	-1.632841	1.340457
H	0.242399	0.020427	0.586679	-1.208778	2.699714
C	-0.643921	-0.422924	-2.125826	-0.251133	1.451906
H	0.128516	-0.022066	-1.808372	0.573561	2.106379
C	-0.560261	0.072620	-2.657064	-1.391599	2.325704
H	0.184034	-0.050852	-3.614343	-1.131391	2.797541
H	0.181493	-0.012797	-2.812901	-2.314330	1.751730
H	0.131653	-0.007392	-1.940245	-1.625850	3.118762
C	-0.557335	0.196111	-3.119294	0.294603	0.461053
C	0.485909	-0.128785	-2.905010	1.554040	-0.124291
C	-0.131829	-0.143556	-4.242203	-0.431973	0.043680
C	-0.027697	0.030348	-3.775091	2.063513	-1.082766
H	0.207048	0.044322	-2.032231	2.131792	0.174552

C	-0.057219	0.023300	-5.116453	0.075976	-0.918400
H	0.121312	0.044393	-4.439994	-1.411255	0.471151
C	-0.173078	-0.123343	-4.889585	1.326043	-1.487408
H	0.153224	0.029024	-3.580238	3.039870	-1.519037
H	0.155214	0.018761	-5.979144	-0.512338	-1.221938
H	0.138474	0.027113	-5.569530	1.722050	-2.236777
H	-0.158943	-0.424359	4.470701	-0.063802	-1.876761
H	0.650555	-0.303360	0.052194	0.446367	-0.064825
B	1.415517	1.191532	3.070227	1.283084	0.830882
C	-1.007811	-0.204598	2.621438	2.523822	-0.155169
H	0.188222	-0.080560	1.557501	2.470023	-0.442060
H	0.114243	-0.072038	2.770155	3.496679	0.340266
H	0.121196	-0.084368	3.207192	2.561126	-1.090763
C	-0.965899	-0.213967	4.675101	1.368831	1.173611
H	0.121524	-0.075596	4.933155	2.295692	1.711335
H	0.127429	-0.078521	4.995500	0.526863	1.808784
H	0.113241	-0.073116	5.300466	1.343134	0.266340
C	-0.910425	-0.185826	2.194000	1.307267	2.219653
H	0.114548	-0.076016	2.442623	0.457678	2.876033
H	0.091346	-0.079955	2.391196	2.226288	2.794532
H	0.239977	-0.103637	1.104676	1.274275	2.043556

X^{H}b

Electronic energy: -1269.805606

Electronic energy + zero-point energy: -1269.371622

Electronic energy + thermal energy correction: -1269.333822

Electronic energy + thermal enthalpy correction: -1269.332640

Electronic energy + thermal free energy correction: -1269.444621

Table S29. Cartesian coordinates, Mulliken and APT charges of all atoms at **X^{H}b** in the reaction of styrene with TMDSO.

Symbol	X	Y	Z	Mulliken	APT
Si	0.377762	2.041780	-0.242265	1.847221	-0.102967
O	-0.301358	-1.588226	0.098113	1.140535	1.349738
Si	1.166656	1.954840	1.211718	0.435786	2.351391
C	-0.803889	-0.437250	0.980274	-1.415826	2.457064
H	0.221182	0.011470	-0.067619	-1.663025	2.659573

H	0.234070	0.070439	1.278591	-1.894045	1.518119
H	0.148748	-0.009761	1.596478	-1.827124	3.266915
C	-0.820878	-0.439182	2.960972	0.903222	1.868926
H	0.182196	-0.005733	3.097884	1.990887	1.865868
H	0.160902	-0.002160	3.681058	0.474726	2.577425
H	0.214135	0.076236	3.191181	0.516092	0.870178
C	-0.689458	-0.438419	-1.458400	3.238836	0.279288
H	0.189918	0.011772	-2.406132	2.832231	0.652816
H	0.178227	-0.001724	-1.049096	3.896637	1.054625
H	0.183340	0.000731	-1.672096	3.848754	-0.606528
C	-0.565611	-0.464027	1.304449	2.541314	-0.906891
H	0.170348	-0.016506	1.825176	3.242019	-0.243331
H	0.329912	0.060189	1.986774	1.719262	-1.154790
H	0.154435	0.009488	1.057425	3.074957	-1.833335
C	-1.454268	-0.482919	-1.059652	0.624635	-1.303486
H	0.066711	0.051972	-0.214260	0.072407	-1.728970
C	-0.443674	0.087958	-1.774996	1.368349	-2.439711
H	0.190215	-0.035024	-2.205684	0.659044	-3.157303
H	0.155394	-0.018435	-2.585517	2.012778	-2.078687
H	0.154529	-0.020015	-1.071024	2.007187	-2.986495
C	0.983399	0.180879	-1.923100	-0.371482	-0.576835
C	0.095386	-0.131765	-1.343282	-1.560756	-0.109661
C	-0.104999	-0.125484	-3.283777	-0.156331	-0.323232
C	-0.201037	0.015052	-2.099017	-2.498748	0.586412
H	-0.072709	0.108140	-0.286038	-1.735983	-0.306091
C	-0.337920	0.006811	-4.042446	-1.095511	0.376808
H	0.113762	0.032706	-3.762336	0.753908	-0.678365
C	-0.044686	-0.114074	-3.454014	-2.271943	0.836176
H	0.157795	0.028902	-1.623485	-3.412823	0.935099
H	0.129677	0.013232	-5.097442	-0.905347	0.561364
H	0.143466	0.019244	-4.044036	-3.005581	1.379809
H	-0.179463	-0.413385	0.936949	1.009319	3.706307
H	-1.497970	-0.436674	1.666341	-0.574026	-0.515196
B	1.407178	0.695920	2.316650	-1.279828	-1.318402
C	-0.753344	-0.157679	1.537419	-1.320043	-2.771214
H	0.189729	-0.084563	0.529860	-1.770275	-2.725700
H	0.148555	-0.086188	1.420444	-0.322803	-3.232672
H	0.134401	-0.103080	2.110563	-1.926785	-3.493628
C	-0.789214	-0.148786	2.447301	-2.804564	-0.706945
H	0.137092	-0.107218	2.989404	-3.460470	-1.410546
H	0.124935	-0.087970	3.008119	-2.839608	0.242672
H	0.196929	-0.087733	1.475294	-3.295572	-0.519539
C	-0.773912	-0.152836	3.819530	-0.633943	-1.522945
H	0.117212	-0.089321	4.435055	-0.651148	-0.608006
H	0.152715	-0.108480	4.382340	-1.207310	-2.280387

H	0.123476	-0.083141	3.807756	0.411236	-1.877563

X^{O}c

Electronic energy: -1269.792894

Electronic energy + zero-point energy: -1269.358728

Electronic energy + thermal energy correction: -1269.320138

Electronic energy + thermal enthalpy correction: -1269.318956

Electronic energy + thermal free energy correction: -1269.434570

Table S30. Cartesian coordinates, Mulliken and APT charges of all atoms at **X^{O}c** in the reaction of styrene with TMDSO.

Symbol	X	Y	Z	Mulliken	APT
Si	1.064838	1.492550	-0.680846	-1.684247	0.063981
O	-0.607684	-1.482244	2.476112	0.240065	0.251565
Si	0.116041	1.844247	2.404236	1.837943	0.009382
C	-0.587613	-0.407546	2.419522	2.327846	-1.820815
H	0.213997	0.005589	3.359330	2.030473	-2.298189
H	0.157183	0.001200	1.600475	1.831478	-2.355989
H	0.175825	-0.023188	2.297980	3.412633	-1.940666
C	-0.765007	-0.380092	0.789036	2.497698	0.743104
H	0.190894	0.000606	0.737054	2.291337	1.818325
H	0.152070	-0.021242	0.694354	3.581203	0.596147
H	0.340423	-0.013738	-0.077867	2.016484	0.272000
C	-0.975051	-0.443709	-0.166231	-0.898553	1.684609
H	0.302904	0.104640	0.901008	-0.650722	1.632036
H	0.191946	0.017798	-0.718279	0.033192	1.855624
H	0.140788	-0.007923	-0.349211	-1.571678	2.530797
C	-0.997066	-0.442524	-0.116208	-0.709415	-1.437796
H	0.388351	0.093826	0.968892	-0.572627	-1.364181
H	0.126960	-0.002719	-0.354441	-1.220087	-2.378238
H	0.247251	0.019143	-0.579027	0.285187	-1.454480
C	-0.583624	-0.406535	-2.606055	-1.835247	0.016267
H	0.111413	-0.044180	-2.885081	-2.381538	0.929028
C	-0.451654	0.086931	-3.052486	-2.660397	-1.198129
H	0.173438	-0.053024	-4.146642	-2.695696	-1.284505
H	0.164880	0.005157	-2.660251	-2.254739	-2.137667
H	0.155450	-0.019168	-2.680315	-3.686948	-1.116057

C	-1.064886	0.167516	-3.232916	-0.469065	0.089838
C	0.597451	-0.122582	-3.551161	0.103164	1.330589
C	0.098720	-0.138571	-3.474033	0.299944	-1.057706
C	-0.059523	0.020679	-4.082731	1.386506	1.425104
H	0.192007	0.036584	-3.364271	-0.470766	2.236541
C	-0.127366	0.021994	-4.007275	1.584885	-0.968507
H	0.173884	0.038668	-3.230424	-0.105884	-2.036317
C	-0.162990	-0.114643	-4.315181	2.136958	0.273027
H	0.152563	0.027453	-4.310872	1.803527	2.402801
H	0.158719	0.027632	-4.176231	2.158685	-1.876319
H	0.155295	0.030697	-4.724528	3.140828	0.343775
H	-0.177992	-0.426103	3.522771	2.598156	0.660047
H	-0.092793	-0.378909	-0.203999	-3.093610	-0.026035
B	1.350048	1.207068	3.670045	-0.728873	0.234268
C	-0.889406	-0.204958	3.050075	-2.239391	0.069092
H	0.147865	-0.076107	2.484611	-2.367237	-0.869552
H	0.094297	-0.081918	3.843896	-3.003806	0.064515
H	0.167735	-0.076490	2.365915	-2.502431	0.893281
C	-0.950812	-0.210952	4.467926	-0.583254	1.662183
H	0.131475	-0.075684	4.886514	0.425931	1.808925
H	0.129491	-0.078027	3.799080	-0.780584	2.516339
H	0.120910	-0.077129	5.308733	-1.291636	1.740835
C	-0.989795	-0.217225	4.657084	-0.366129	-1.031785
H	0.149360	-0.074499	4.130999	-0.426769	-1.999608
H	0.125961	-0.078787	5.087576	0.648336	-0.960661
H	0.122830	-0.069560	5.509480	-1.061891	-1.087958

X^{H}c

Electronic energy: -1269.804025

Electronic energy + zero-point energy: -1269.370458

Electronic energy + thermal energy correction: -1269.331247

Electronic energy + thermal enthalpy correction: -1269.330066

Electronic energy + thermal free energy correction: -1269.448323

Table S31. Cartesian coordinates, Mulliken and APT charges of all atoms at **X^{H}c** in the reaction of styrene with TMDSO.

Symbol	X	Y	Z	Mulliken	APT

Si	1.160731	2.079337	-0.121500	-1.032156	-0.588198
O	-0.339795	-1.677306	1.334200	-1.573232	-0.003760
Si	0.981281	2.024555	2.910544	-1.141661	0.289685
C	-0.877353	-0.416037	3.772001	-2.755214	0.764813
H	0.178377	0.001025	3.301594	-3.207979	1.644753
H	0.159744	-0.007607	3.720230	-3.481822	-0.054720
H	0.164252	-0.006614	4.829698	-2.580933	0.998184
C	-0.842772	-0.439518	3.723639	-0.475444	-1.260700
H	0.229958	0.055425	3.220422	0.438546	-1.588463
H	0.166752	0.001475	4.777161	-0.232014	-1.077744
H	0.159997	-0.007371	3.676721	-1.224146	-2.061824
C	-0.508441	-0.460818	0.064824	-0.189715	-2.254715
H	0.175083	-0.009961	0.701716	-0.790800	-2.915501
H	0.186173	0.006981	-0.910261	-0.069260	-2.742237
H	0.238206	0.058920	0.523053	0.797114	-2.128355
C	-0.903902	-0.428090	-1.159063	-2.594851	-0.789460
H	0.156326	-0.001909	-0.634169	-3.314280	-1.429020
H	0.180022	0.008731	-1.337193	-3.077499	0.179433
H	0.188379	0.005273	-2.134524	-2.376140	-1.239073
C	-0.952670	-0.496725	-0.971549	0.104721	0.680918
H	-0.123606	0.054387	-0.558050	1.103228	0.495914
C	-0.601291	0.079531	-0.580946	-0.313135	2.103034
H	0.177604	-0.046117	-1.150413	0.253872	2.851064
H	0.133071	-0.025326	-0.743498	-1.383217	2.290850
H	0.130592	0.016390	0.481963	-0.104639	2.260165
C	-0.033267	0.219136	-2.449596	0.137714	0.404675
C	-0.269457	-0.138111	-2.948905	0.975496	-0.605994
C	-0.002808	-0.147213	-3.366231	-0.674503	1.085834
C	0.025634	0.035607	-4.302550	0.991946	-0.929983
H	0.101337	0.052297	-2.254937	1.628994	-1.132479
C	-0.050618	0.024898	-4.723514	-0.660760	0.764367
H	0.141900	0.046640	-3.012991	-1.328805	1.878976
C	-0.138675	-0.130039	-5.200574	0.169279	-0.248376
H	0.162691	0.023386	-4.659212	1.656242	-1.713470
H	0.142696	0.015792	-5.410888	-1.302402	1.311077
H	0.136829	0.023023	-6.258340	0.181758	-0.498085
H	0.036126	-0.368006	3.020203	-0.220110	1.442914
H	-1.500168	-0.449237	1.576869	1.199302	-0.073116
B	1.818741	0.704502	1.751461	2.407002	0.187384
C	-0.693484	-0.143772	1.421667	2.667267	1.779118
H	0.118378	-0.078323	2.050322	2.057294	2.449909
H	0.140519	-0.104128	1.612913	3.721707	2.046056
H	0.210184	-0.090115	0.373032	2.466458	2.059285
C	-0.817933	-0.137687	0.745454	3.301894	-0.764722
H	0.110849	-0.086010	0.930099	3.160160	-1.843616

H	0.206923	-0.105673	-0.326393	3.093380	-0.596622
H	0.141680	-0.110591	0.877284	4.380257	-0.567923
C	-0.982766	-0.153166	3.321865	2.789411	-0.125436
H	0.150406	-0.077316	4.034858	2.152585	0.426533
H	0.102409	-0.087646	3.588272	2.712401	-1.193370
H	0.125158	-0.106879	3.537802	3.830404	0.172596

XI°

Electronic energy: -680.018574

Sum of electronic and zero-point Energies= -679.787872

Sum of electronic and thermal Energies= -679.768156

Sum of electronic and thermal Enthalpies= -679.766974

Sum of electronic and thermal Free Energies= -679.842435

Table S32. Cartesian coordinates, Mulliken and APT charges of all atoms at **XI°** in the reaction of styrene with TMDSO.

Symbol	X	Y	Z	Mulliken	APT
Si	-1.824013	-0.329375	-0.469992	1.088412	1.496089
C	-3.625706	0.003376	-0.036190	-0.842422	-0.420083
H	-4.292417	-0.607990	-0.652202	0.184363	-0.005007
H	-3.820079	-0.240801	1.013649	0.180948	0.014222
H	-3.889908	1.052974	-0.195160	0.184617	0.017690
C	-1.434756	-2.161160	-0.314367	-0.871325	-0.413284
H	-2.090391	-2.756176	-0.957329	0.179840	0.008292
H	-0.397944	-2.367786	-0.595523	0.206915	0.024152
H	-1.578639	-2.497799	0.718169	0.177261	0.015904
C	-0.683389	0.684689	0.670097	-0.538950	-0.386170
H	-0.883289	0.337128	1.693364	0.138493	-0.032212
C	-1.017209	2.182569	0.588808	-0.534680	0.076866
H	-0.294269	2.778922	1.155864	0.183864	-0.031677
H	-1.013091	2.548514	-0.443533	0.177547	-0.002927
H	-2.012866	2.377882	0.997825	0.158988	-0.018021
C	0.753607	0.356890	0.344800	-0.806136	0.155743
C	1.442344	-0.622227	1.074113	0.221261	-0.115759
C	1.425402	0.977191	-0.717548	0.114606	-0.130244
C	2.753554	-0.970892	0.757303	-0.083291	0.010437
H	0.939199	-1.116222	1.902544	0.149269	0.033677

C	2.738666	0.633214	-1.036064	-0.145196	0.020375
H	0.918326	1.737494	-1.305270	0.128112	0.047598
C	3.409759	-0.342902	-0.301142	-0.096799	-0.105220
H	3.264620	-1.730469	1.341416	0.159821	0.031317
H	3.237791	1.130398	-1.862489	0.152655	0.030649
H	4.432453	-0.609441	-0.548238	0.144239	0.036940
H	-1.556945	0.130125	-1.862064	-0.012408	-0.359347

XI^{H}

Electronic energy: -1124.59207

Sum of electronic and zero-point Energies= -1124.281111

Sum of electronic and thermal Energies= -1124.251281

Sum of electronic and thermal Enthalpies= -1124.250100

Sum of electronic and thermal Free Energies= -1124.349877

Table S33. Cartesian coordinates, Mulliken and APT charges of all atoms at **XI^{H}** in the reaction of styrene with TMDSO.

Symbol	X	Y	Z	Mulliken	APT
Si	0.451287	-0.685077	-0.074890	0.417892	2.036484
O	1.962205	-0.475378	-0.742187	-0.370001	-1.660258
Si	3.506435	-0.008701	-0.350279	0.894410	1.965765
C	3.593442	1.853612	-0.167981	-0.798947	-0.445065
H	3.334124	2.362805	-1.100686	0.203199	0.021938
H	2.902955	2.197544	0.610297	0.175441	0.019760
H	4.604100	2.163807	0.117991	0.173371	0.014166
C	4.057630	-0.833143	1.240500	-0.767737	-0.446479
H	3.859987	-1.909463	1.218221	0.206744	0.017973
H	5.132893	-0.690488	1.390815	0.188594	0.017837
H	3.539267	-0.407012	2.105496	0.188281	0.026438
C	-0.098016	-2.444273	-0.392736	-0.829923	-0.448991
H	0.620308	-3.146981	0.041962	0.175016	0.010321
H	-1.078797	-2.637741	0.052918	0.211900	0.024663
H	-0.163166	-2.647917	-1.466384	0.185855	0.020090
C	0.510542	-0.337643	1.769432	-0.557227	-0.461509
H	1.111568	-1.095398	2.282754	0.211875	0.018472
H	0.943618	0.644635	1.989490	0.255127	0.014348
H	-0.498307	-0.364974	2.194515	0.197527	0.036055

C	-0.698160	0.554408	-0.936977	-1.029175	-0.429541
H	-0.696218	0.268106	-1.997838	0.087465	-0.024515
C	-0.135927	1.978313	-0.819432	-0.261527	0.065984
H	-0.826214	2.713673	-1.247019	0.188695	-0.034707
H	0.050833	2.266354	0.221215	0.227268	-0.002250
H	0.817309	2.051488	-1.349832	0.174113	-0.005547
C	-2.106589	0.392946	-0.417992	-0.203488	0.164502
C	-2.972637	-0.539557	-1.007826	0.624183	-0.115998
C	-2.582439	1.124048	0.678791	-0.457287	-0.130307
C	-4.261370	-0.740599	-0.519955	-0.158828	0.012843
H	-2.626161	-1.113794	-1.864542	0.165063	0.036325
C	-3.873107	0.926845	1.170149	-0.194587	0.016039
H	-1.940699	1.858496	1.157609	0.102047	0.047936
C	-4.719047	-0.006996	0.575033	0.016582	-0.102531
H	-4.911106	-1.466830	-0.999231	0.150790	0.031751
H	-4.217531	1.508384	2.020200	0.156842	0.030909
H	-5.724071	-0.158485	0.955766	0.153772	0.036287
H	4.373637	-0.448062	-1.469895	-0.103323	-0.379188

1,1,1,3,3-pentamethyldisiloxane (PMDSO)

V

Electronic energy: -1309.111966

Electronic energy + zero-point energy: -1308.651245

Electronic energy + thermal energy correction: -1308.609135

Electronic energy + thermal enthalpy correction: -1308.607953

Electronic energy + thermal free energy correction: -1308.731847

Table S34. Cartesian coordinates, Mulliken and APT charges of all atoms at **V** in the reaction of styrene with PMDSO.

Symbol	X	Y	Z	Mulliken	APT
Si	0.287311	1.829929	1.623197	1.498345	0.504116
O	-0.369439	-1.531500	3.146984	0.974160	0.038359
Si	0.876455	1.973571	3.729203	-0.457819	-0.563740
C	-0.610650	-0.439452	3.667519	-1.776072	0.770672
H	0.182891	-0.000947	4.300595	-1.503158	1.622786
H	0.212298	0.032822	2.641184	-1.894837	1.137556

H	0.184765	0.006616	4.007656	-2.746808	0.389795
C	-0.812690	-0.440071	2.708705	-0.997350	-2.046298
H	0.180577	0.006862	2.713080	-0.230277	-2.829625
H	0.154890	0.008953	3.099003	-1.926890	-2.478591
H	0.276823	0.021903	1.666343	-1.175277	-1.757126
C	-0.765374	-0.404404	1.885620	3.040624	1.541686
H	0.173256	0.001629	2.376136	3.824608	0.953506
H	0.172782	0.018533	0.926363	3.432099	1.899342
H	0.178511	0.007800	2.512754	2.826016	2.413455
C	-0.596225	-0.436093	0.631278	1.917111	-1.026573
H	0.174959	-0.000920	1.161176	2.669197	-1.624500
H	0.256300	0.034916	0.457921	1.033929	-1.650475
H	0.227999	0.047882	-0.353878	2.314425	-0.758156
C	-0.017878	-0.443392	-2.688097	0.623196	-0.349000
H	0.112995	-0.066904	-2.217155	0.855211	-1.318433
C	-0.556098	0.129747	-2.891946	1.935072	0.418787
H	0.156533	-0.025053	-3.626545	1.819977	1.226484
H	0.146423	-0.100951	-1.964659	2.322331	0.875261
H	0.138248	-0.034230	-3.284762	2.705603	-0.253478
C	-0.768537	0.312861	-1.793140	-0.329717	0.368107
C	-0.087817	-0.174636	-0.949643	-1.209733	-0.337005
C	-0.274423	-0.165163	-1.783806	-0.440806	1.770073
C	-0.199462	0.027282	-0.147843	-2.142758	0.314246
H	0.113569	0.036711	-0.948282	-1.160179	-1.425558
C	-0.141008	0.068179	-0.984694	-1.373593	2.428809
H	0.123928	0.039832	-2.430961	0.209106	2.354123
C	-0.119540	-0.197870	-0.156581	-2.233196	1.708315
H	0.147211	0.012897	0.485470	-2.810505	-0.268820
H	0.157253	0.009143	-1.010738	-1.430063	3.515289
H	0.152380	0.024734	0.467132	-2.962079	2.220785
H	0.076336	-0.300329	0.993445	0.434795	1.319157
B	1.076460	0.803580	-4.175752	-0.105151	-0.748219
C	-0.804005	-0.171412	-3.915767	-1.350429	-1.791945
H	0.167626	-0.063692	-3.349003	-2.184968	-1.348292
H	0.121354	-0.099642	-4.875454	-1.772251	-2.134415
H	0.128765	-0.086542	-3.369556	-1.038754	-2.700420
C	-0.872399	-0.162152	-5.108020	1.035718	-1.489134
H	0.135286	-0.080377	-5.438003	1.841549	-0.812706
H	0.142121	-0.089160	-4.592995	1.521044	-2.337223
H	0.120812	-0.111761	-6.024744	0.579217	-1.897938
C	-0.750243	-0.179543	-4.938031	-0.664747	0.598687
H	0.133007	-0.078413	-5.113701	0.108228	1.367314
H	0.129275	-0.094842	-5.932151	-1.060964	0.331900
H	0.168378	-0.066932	-4.394825	-1.486338	1.093104
C	-0.938449	-0.425762	5.507252	-0.149660	-1.089486

H	0.168336	0.003032	6.113156	0.180360	-0.238269
H	0.157330	0.005866	5.961942	-1.060157	-1.497673
H	0.170795	0.006865	5.556499	0.628950	-1.858853

VIa

Electronic energy: -1309.061721

Electronic energy + zero-point energy: -1308.601264

Electronic energy + thermal energy correction: -1308.558659

Electronic energy + thermal enthalpy correction: -1308.557477

Electronic energy + thermal free energy correction: -1269.683501

Table S35. Cartesian coordinates, Mulliken and APT charges of all atoms at **VIa** in the reaction of styrene with PMDSO.

Symbol	X	Y	Z	Mulliken	APT
Si	0.702615	2.245353	-1.087166	-1.095010	-0.242149
O	-0.479714	-1.621435	-2.832250	-1.170910	-0.256931
Si	0.756565	1.985969	-3.887840	0.031240	0.108575
C	-0.682626	-0.436046	-4.067861	0.247358	1.974296
H	0.182694	-0.002642	-4.379683	-0.691069	2.447666
H	0.187087	0.017565	-3.111562	0.546664	2.417970
H	0.164996	-0.012421	-4.812128	1.016661	2.216234
C	-0.732566	-0.440504	-3.329892	1.674723	-0.629401
H	0.183384	0.002399	-3.251789	1.607083	-1.721161
H	0.151410	-0.003008	-4.034332	2.481157	-0.388965
H	0.274175	0.015376	-2.342952	1.963159	-0.245030
C	-0.825459	-0.447183	-0.740278	-2.938751	-0.006209
H	0.147541	-0.043449	-1.530999	-3.497030	-0.521382
H	0.156145	0.008152	0.233548	-3.239342	-0.404155
H	0.168296	0.004218	-0.775232	-3.213260	1.053962
C	-0.840339	-0.458991	-0.788066	-0.513343	-2.016449
H	0.142358	-0.019694	-1.633412	-0.837723	-2.635378
H	0.269627	0.033499	-0.717468	0.578368	-2.070301
H	0.147730	-0.002159	0.137555	-0.923634	-2.433466
C	-0.614169	-1.350725	1.463192	-0.717503	0.239601
H	0.296534	0.060815	1.764794	-1.292637	-0.634570
C	-0.628723	0.288632	1.496231	-1.384419	1.589569
H	0.126450	-0.091558	2.309102	-1.016761	2.242298

H	0.090011	-0.078672	0.561928	-1.225138	2.159921
H	0.153728	-0.021559	1.630451	-2.466769	1.491253
C	-1.317794	0.691263	1.485420	0.704468	0.151211
C	0.505474	-0.319723	1.659274	1.405294	-1.078734
C	0.053479	-0.383834	1.277892	1.530078	1.294092
C	-0.130497	0.237735	1.648760	2.787927	-1.152624
H	0.138852	0.018135	1.802364	0.825220	-1.987990
C	-0.218889	0.247211	1.267638	2.919659	1.208495
H	0.159827	0.032302	1.131303	1.061722	2.264182
C	-0.214529	-0.434273	1.456232	3.576417	-0.008124
H	0.127356	0.000488	1.792711	3.266070	-2.120768
H	0.131657	-0.002885	1.108652	3.500261	2.116544
H	0.131152	0.010604	1.449609	4.661149	-0.069083
H	0.323745	-0.355240	-0.965153	-0.156047	0.908038
B	1.092105	1.144780	4.207914	-0.921736	-0.092392
C	-0.811056	-0.319578	4.291479	-0.730987	-1.668093
H	0.203749	0.014961	3.977384	0.262754	-2.003309
H	0.145687	-0.033419	5.353822	-0.836376	-1.948069
H	0.153511	-0.014765	3.742061	-1.490505	-2.239143
C	-0.817169	-0.310257	4.306156	-2.408562	0.467283
H	0.144804	-0.009699	4.126224	-2.499652	1.544129
H	0.169387	-0.004859	3.641455	-3.108433	-0.055487
H	0.147637	-0.050488	5.335093	-2.760514	0.281035
C	-0.777610	-0.329790	4.559129	0.298874	0.851402
H	0.174415	0.011181	4.084126	0.226657	1.837976
H	0.138917	-0.031618	5.649008	0.292112	1.025883
H	0.203497	-0.000695	4.295823	1.272696	0.422593
C	-0.864451	-0.428144	-5.574583	-0.427364	-0.601584
H	0.169888	-0.001571	-5.926407	-1.376088	-0.180023
H	0.162499	-0.012695	-6.325437	0.342145	-0.382856
H	0.176607	0.002941	-5.516279	-0.547364	-1.689510

VIb

Electronic energy: -1309.061430

Electronic energy + zero-point energy: -1308.601124

Electronic energy + thermal energy correction: -1308.559714

Electronic energy + thermal enthalpy correction: -1308.558532

Electronic energy + thermal free energy correction: -1269.679187

Table S36. Cartesian coordinates, Mulliken and APT charges of all atoms at **VIb** in the reaction of styrene with PMDSO.

Symbol	X	Y	Z	Mulliken	APT
Si	1.111992	2.252972	1.056521	-0.969688	-0.411402
O	-0.545941	-1.644663	2.782647	-1.114319	-0.147385
Si	0.928278	1.996031	3.901612	-0.060835	0.411648
C	-0.737654	-0.439189	4.660871	0.947499	-0.994868
H	0.184196	-0.001169	5.038209	0.288433	-1.785863
H	0.174498	0.011013	3.918801	1.617288	-1.443924
H	0.169080	-0.010995	5.496938	1.560571	-0.634529
C	-0.770878	-0.443189	3.172171	1.144878	1.666044
H	0.193275	0.007026	2.764541	0.612511	2.533151
H	0.150489	-0.009119	3.933709	1.850592	2.022066
H	0.219859	0.009798	2.349251	1.722960	1.227456
C	-0.908398	-0.432127	1.097374	0.323462	-1.789315
H	0.288639	0.009828	1.160575	1.328813	-1.350909
H	0.149084	-0.019812	1.991397	0.159393	-2.403915
H	0.182922	0.019450	0.212721	0.319912	-2.430994
C	-0.890515	-0.441815	0.715128	-2.763865	-0.910223
H	0.140226	0.009589	0.297124	-2.818840	-1.921087
H	0.166116	-0.050044	1.650551	-3.333122	-0.886463
H	0.145497	0.002106	-0.005547	-3.234493	-0.231928
C	-0.895341	-1.283868	-1.535178	-0.708175	-0.435708
H	0.195819	0.060299	-1.510481	-1.320096	0.464673
C	-0.607476	0.281857	-2.044399	-1.347909	-1.701398
H	0.123986	-0.103976	-3.071420	-1.043154	-1.975938
H	0.101063	-0.077534	-1.416914	-1.107961	-2.577866
H	0.164427	-0.025797	-2.050682	-2.439093	-1.609486
C	-1.249553	0.690088	-1.607068	0.706003	-0.269077
C	0.248074	-0.355015	-1.251991	1.356113	0.950822
C	0.252695	-0.368955	-1.978492	1.576981	-1.335227
C	0.064383	0.243324	-1.253584	2.737107	1.079163
H	0.082463	0.024850	-0.960185	0.742330	1.799639
C	-0.356321	0.250160	-1.978473	2.959230	-1.193960
H	0.149252	0.029003	-2.271360	1.146387	-2.290530
C	-0.134956	-0.445496	-1.614597	3.568208	0.010550
H	0.137395	0.001573	-0.966552	3.177688	2.033324
H	0.127487	-0.006206	-2.271661	3.575542	-2.043099
H	0.128472	0.011630	-1.615569	4.649480	0.116915
H	0.227460	-0.365463	0.703883	-0.542762	0.966339
B	1.054182	1.073687	-3.986733	-1.116040	0.910711
C	-0.788463	-0.303441	-4.180880	-2.592490	0.352742
H	0.180579	-0.003569	-3.319291	-3.241513	0.554786

H	0.151021	-0.042392	-5.036294	-3.037131	0.889255
H	0.141478	-0.010702	-4.410930	-2.643159	-0.717291
C	-0.778808	-0.323826	-4.730954	0.093494	0.213844
H	0.194207	0.011047	-4.379614	1.075570	0.550772
H	0.176169	0.011626	-4.657107	0.067824	-0.880800
H	0.139686	-0.027191	-5.804798	0.017954	0.457329
C	-0.780544	-0.312618	-3.457681	-0.938023	2.395670
H	0.149280	-0.017846	-2.759093	-1.718063	2.723041
H	0.219074	0.026423	-2.999610	0.043000	2.561678
H	0.152589	-0.026195	-4.333198	-0.990405	3.065177
C	-0.840973	-0.429856	5.294242	-1.035471	1.233494
H	0.171630	-0.001229	5.746153	-1.735591	0.521216
H	0.163031	-0.013681	6.083859	-0.371127	1.606192
H	0.185769	0.003597	4.912303	-1.619912	2.078372

VIc

Electronic energy: -1309.059006

Electronic energy + zero-point energy: -1308.598417

Electronic energy + thermal energy correction: -1308.555894

Electronic energy + thermal enthalpy correction: -1308.554712

Electronic energy + thermal free energy correction: -1308.678609

Table S37. Cartesian coordinates, Mulliken and APT charges of all atoms at **VIc** in the reaction of styrene with PMDSO.

Symbol	X	Y	Z	Mulliken	APT
Si	1.076987	2.378791	1.192476	-0.622403	-0.108035
O	-0.447722	-1.720026	2.911622	-0.946133	-0.038065
Si	0.742714	2.058094	4.328101	-0.149801	0.080308
C	-0.684426	-0.452361	4.892677	0.529086	-1.590533
H	0.188489	0.002170	4.929551	-0.272771	-2.337424
H	0.180921	0.017166	4.206617	1.298165	-1.962207
H	0.166149	-0.012825	5.894232	0.972078	-1.517568
C	-0.743719	-0.449928	4.235974	1.295219	1.296489
H	0.187332	0.004563	4.085397	0.939168	2.322284
H	0.153086	-0.006303	5.160492	1.886075	1.276249
H	0.190295	0.005493	3.401248	1.964684	1.056175
C	-0.754882	-0.439341	0.868842	-0.161065	1.698035

H	0.144100	-0.031756	1.804813	-0.152463	2.268029
H	0.244801	0.029243	0.407321	0.830506	1.762835
H	0.165108	-0.005111	0.181315	-0.871229	2.171483
C	-0.912618	-0.440690	1.238208	0.816994	-1.335295
H	0.241920	-0.025459	1.900388	1.592907	-0.925811
H	0.184569	-0.012460	1.677498	0.487943	-2.285293
H	0.174429	0.047422	0.265686	1.277133	-1.526996
C	-0.928864	-1.324649	-1.407152	-0.613961	-0.394658
H	0.324770	0.051289	-1.451283	-1.317606	0.437173
C	-0.649615	0.278024	-1.443908	-1.187480	-1.790202
H	0.124959	-0.107425	-2.436666	-1.126825	-2.273564
H	0.150342	-0.066612	-0.744859	-0.671457	-2.471641
H	0.126953	-0.025000	-1.151491	-2.242576	-1.784161
C	-0.633621	0.730029	-1.932177	0.690676	-0.160745
C	0.219758	-0.348326	-2.148000	1.214447	1.149930
C	0.204780	-0.410432	-2.262243	1.580675	-1.225721
C	-0.350893	0.261992	-2.642617	2.489380	1.368422
H	0.156559	0.020502	-1.918505	0.578637	2.002513
C	-0.327778	0.268549	-2.753768	2.861661	-0.995012
H	0.169561	0.031224	-2.144130	1.241193	-2.252331
C	-0.184577	-0.462504	-2.955109	3.343247	0.299854
H	0.123419	-0.000923	-2.791528	2.829583	2.392610
H	0.122476	-0.005431	-2.993611	3.494228	-1.849020
H	0.124159	0.010065	-3.344407	4.342390	0.474578
H	-0.047094	-0.443975	0.820112	-1.980382	-0.570817
B	0.922172	1.041000	-3.939727	-1.835126	0.267788
C	-0.775267	-0.315985	-4.800417	-0.739280	-0.478592
H	0.177610	0.008538	-4.525846	-0.619220	-1.533789
H	0.139447	-0.024809	-5.853267	-1.070752	-0.462120
H	0.216087	0.010648	-4.756046	0.247690	-0.003438
C	-0.773056	-0.311539	-3.814763	-1.771243	1.848889
H	0.148458	-0.022367	-4.737359	-2.211422	2.264471
H	0.159064	-0.010208	-2.977949	-2.352835	2.255690
H	0.201976	0.016904	-3.757690	-0.747350	2.232942
C	-0.786478	-0.305303	-3.591691	-3.207903	-0.452245
H	0.176972	0.002238	-2.650512	-3.646652	-0.098605
H	0.151136	-0.036903	-4.387020	-3.926754	-0.191506
H	0.146657	-0.007078	-3.557192	-3.147952	-1.545464
C	-0.846463	-0.433943	5.650128	-1.349858	0.690604
H	0.175802	0.001749	5.770141	-2.181807	-0.012781
H	0.161240	-0.017360	6.623554	-0.856958	0.805487
H	0.181818	0.001337	5.364676	-1.773389	1.660459

VIq

Electronic energy: -1309.052990

Electronic energy + zero-point energy: -1308.594196

Electronic energy + thermal energy correction: -1308.551815

Electronic energy + thermal enthalpy correction: -1308.550633

Electronic energy + thermal free energy correction: -1308.676444

Table S38. Cartesian coordinates, Mulliken and APT charges of all atoms at **VIq** in the reaction of styrene with PMDSO.

Symbol	X	Y	Z	Mulliken	APT
Si	1.149116	2.431953	1.398019	-0.813723	0.072684
O	-0.514352	-1.676623	3.130994	-1.095944	0.260839
Si	0.543169	1.938584	4.511523	-0.241844	0.350729
C	-0.700244	-0.430076	5.231026	0.133077	-1.353757
H	0.195857	0.003848	5.330182	-0.786489	-1.941498
H	0.174031	0.019649	4.583301	0.817727	-1.912690
H	0.178565	-0.014298	6.223695	0.592857	-1.268663
C	-0.733379	-0.430705	4.302300	1.392271	1.274843
H	0.197985	0.006525	4.029753	1.225869	2.322553
H	0.158388	-0.005331	5.231900	1.974830	1.253063
H	0.186541	0.012368	3.510258	2.001365	0.823035
C	-0.725883	-0.453323	0.978997	-2.646257	-0.166618
H	0.155212	-0.046557	1.761211	-3.232611	0.327808
H	0.174474	0.003964	0.001559	-2.927271	0.234548
H	0.175859	0.000174	0.992242	-2.904533	-1.232436
C	-0.706284	-0.451301	1.038778	-0.078642	1.778494
H	0.150456	-0.033281	1.924918	-0.161010	2.418297
H	0.276244	0.025292	0.759379	0.978049	1.698217
H	0.140416	-0.005581	0.206342	-0.595409	2.268469
C	-0.914866	-1.254918	-1.226596	-0.539250	-0.331783
H	0.322602	0.037614	-1.423558	-1.142352	0.556607
C	-0.473573	0.268929	-1.389492	-1.227776	-1.668223
H	0.157253	-0.100093	-2.358873	-1.017702	-2.159801
H	0.126028	-0.065662	-0.614461	-0.930684	-2.395553
H	0.128087	-0.019242	-1.313078	-2.314132	-1.563523
C	-1.124704	0.726187	-1.573766	0.840261	-0.230299
C	0.550119	-0.339628	-1.762005	1.499797	1.022775
C	0.005480	-0.430749	-1.745879	1.673120	-1.376764
C	-0.238680	0.259252	-2.058219	2.848214	1.117587
H	0.171758	0.018532	-1.671530	0.909875	1.932767

C	-0.253734	0.268354	-2.039141	3.030440	-1.270378
H	0.165021	0.030023	-1.648230	1.234858	-2.367695
C	-0.261526	-0.461241	-2.196606	3.646713	-0.028833
H	0.129473	-0.000779	-2.190146	3.292007	2.103713
H	0.122829	-0.006321	-2.153595	3.615794	-2.182227
H	0.119645	0.009987	-2.429198	4.705113	0.049104
H	-0.160373	-0.422946	5.537849	-1.043328	1.086378
B	1.039315	0.975553	-4.018743	-1.479922	0.363612
C	-0.805966	-0.319194	-3.990268	-0.960285	1.859675
H	0.165831	-0.005910	-3.172209	-1.377666	2.459867
H	0.208539	0.023167	-3.957042	0.132667	1.929538
H	0.146966	-0.020146	-4.931284	-1.284036	2.336340
C	-0.787897	-0.298767	-3.640332	-2.989834	0.064248
H	0.178142	-0.001660	-2.789535	-3.355425	0.651995
H	0.145484	-0.030919	-4.511060	-3.601242	0.358390
H	0.156351	-0.001206	-3.453280	-3.197276	-0.994719
C	-0.847041	-0.308272	-4.729185	-0.582118	-0.725050
H	0.122656	-0.032953	-4.917831	-1.081396	-1.682710
H	0.136705	-0.019924	-5.681125	-0.184774	-0.343469
H	0.282207	0.072027	-4.098039	0.298808	-0.920810
C	-0.791249	-0.435737	1.567665	0.368629	-1.398863
H	0.241879	-0.025363	2.209105	1.205861	-1.090271
H	0.185103	-0.017382	2.086974	-0.146067	-2.218144
H	0.175965	0.034107	0.633917	0.792653	-1.771368

VIIa

Electronic energy: -1309.067073

Electronic energy + zero-point energy: -1308.608932

Electronic energy + thermal energy correction: -1308.566534

Electronic energy + thermal enthalpy correction: -1308.565352

Electronic energy + thermal free energy correction: -1308.691192

Table S39. Cartesian coordinates, Mulliken and APT charges of all atoms at **VIIa** in the reaction of styrene with PMDSO.

Symbol	X	Y	Z	Mulliken	APT
Si	1.090728	2.226960	-1.352510	-1.029429	-0.502440
O	-0.510748	-1.626274	-3.152103	-0.813032	-0.258421

Si	0.753822	1.981637	-3.950860	0.401305	0.464463
C	-0.727520	-0.433413	-3.945293	0.252736	2.352692
H	0.176678	-0.010122	-4.374650	-0.706103	2.667410
H	0.174668	0.008158	-2.918679	0.299634	2.733701
H	0.157639	-0.022647	-4.524011	1.057976	2.823857
C	-0.739625	-0.434457	-3.230129	2.098426	0.033706
H	0.182923	-0.001314	-3.273565	2.278539	-1.047441
H	0.144853	-0.016300	-3.774646	2.908144	0.537032
H	0.255110	0.006078	-2.176616	2.157422	0.334842
C	-0.904959	-0.424823	-1.582945	-2.916816	-0.695301
H	0.145331	-0.047071	-2.517848	-3.097062	-1.236644
H	0.159302	-0.022104	-0.754754	-3.405791	-1.221010
H	0.165976	-0.014731	-1.685921	-3.387186	0.291128
C	-0.965817	-0.423019	-1.351495	-0.067620	-2.154026
H	0.140574	-0.029871	-2.351314	-0.106017	-2.599681
H	0.252332	0.004656	-1.088147	0.985964	-1.995084
H	0.129138	-0.023597	-0.627228	-0.478299	-2.868251
C	-0.664608	-0.879833	0.797302	-1.234992	-0.456325
H	0.041962	-0.051798	1.073706	-1.722334	-1.402655
C	-0.449168	0.171085	1.093899	-2.130826	0.742400
H	0.123446	-0.089072	2.136600	-2.066178	1.090694
H	0.134301	-0.030677	0.455325	-1.874216	1.602797
H	0.141999	-0.037784	0.891471	-3.179918	0.500498
C	-1.607234	0.456625	1.373630	0.111086	-0.378579
C	0.736063	-0.209688	1.694610	0.855518	-1.539242
C	0.047583	-0.244220	1.547435	0.780943	0.853436
C	-0.069758	0.100845	2.094959	2.183385	-1.478292
H	0.159925	0.025678	1.600274	0.369320	-2.508512
C	-0.089191	0.100763	1.954039	2.114430	0.916222
H	0.168928	0.046864	1.337719	0.248094	1.777381
C	-0.200476	-0.262651	2.221084	2.835611	-0.245676
H	0.147519	0.011023	2.320052	2.717140	-2.399649
H	0.146964	0.012729	2.062786	2.591690	1.888425
H	0.145931	0.021437	2.536951	3.874361	-0.196876
H	0.298874	-0.391080	-1.113457	-0.353421	0.833017
B	0.820088	0.851114	4.774442	-0.524086	0.512677
C	-0.773961	-0.296933	5.283284	0.785949	-0.198175
H	0.272767	0.079700	4.382581	1.391476	-0.393196
H	0.135891	-0.022327	5.930068	1.404529	0.437233
H	0.144883	-0.024904	5.770744	0.622827	-1.166194
C	-0.784425	-0.294625	4.486965	-1.822359	-0.336912
H	0.145976	-0.016099	4.419254	-2.745850	0.250074
H	0.297813	0.066550	3.503042	-1.681605	-0.813979
H	0.141868	-0.026931	5.206889	-1.963277	-1.152894
C	-0.807709	-0.296319	4.608382	-0.523826	2.085656

H	0.182740	0.003480	3.858780	-1.230323	2.457913
H	0.135473	-0.015435	5.579618	-0.842773	2.502847
H	0.180739	0.003255	4.397965	0.470009	2.497355
C	-0.880468	-0.423518	-5.760754	0.386199	-0.093246
H	0.165888	-0.006582	-6.234656	-0.570354	0.157408
H	0.155986	-0.022437	-6.337610	1.189057	0.384005
H	0.172983	-0.005983	-5.828765	0.515893	-1.179911

VIIb

Electronic energy: -1309.065665

Electronic energy + zero-point energy: -1308.605941

Electronic energy + thermal energy correction: -1308.563083

Electronic energy + thermal enthalpy correction: -1308.561901

Electronic energy + thermal free energy correction: -1308.687969

Table S40. Cartesian coordinates, Mulliken and APT charges of all atoms at **VIIb** in the reaction of styrene with PMDSO.

Symbol	X	Y	Z	Mulliken	APT
Si	1.508315	2.239681	1.321360	1.113453	-0.137445
O	-0.528872	-1.639669	3.144069	0.894331	-0.127741
Si	0.937819	1.979944	4.095362	-0.417160	-0.103094
C	-0.747578	-0.434105	4.616858	-0.895540	1.656975
H	0.177950	-0.007213	5.086049	-0.044110	2.164993
H	0.168362	0.003481	3.750306	-1.202039	2.254277
H	0.162518	-0.020360	5.335735	-1.725532	1.647893
C	-0.777032	-0.428081	3.279204	-1.942921	-0.873485
H	0.183778	0.000504	3.029577	-1.760250	-1.925266
H	0.145388	-0.016599	3.943918	-2.815543	-0.822497
H	0.225151	-0.001175	2.342647	-2.195042	-0.360823
C	-0.996044	-0.402018	1.188030	0.434141	1.648527
H	0.267719	-0.007481	1.271793	-0.662133	1.615700
H	0.165786	-0.022539	2.030743	0.808292	2.241280
H	0.116755	-0.016205	0.251693	0.664500	2.166396
C	-0.946515	-0.425504	1.488924	3.002575	-0.373743
H	0.138222	-0.013075	1.178287	3.525840	0.540237
H	0.166155	-0.046622	2.530009	3.264304	-0.581933
H	0.134233	-0.022696	0.852351	3.369314	-1.188716

C	-0.743379	-0.873843	-0.835766	1.245431	-0.348960
H	0.090588	-0.031396	-0.894083	1.513751	-1.413680
C	-0.601008	0.165801	-1.446949	2.336599	0.523003
H	0.155151	-0.097442	-2.544663	2.271236	0.608626
H	0.145953	-0.037947	-1.050063	2.315253	1.549168
H	0.157921	-0.032875	-1.211585	3.326290	0.117638
C	-1.226645	0.460018	-1.368899	-0.105320	-0.136953
C	0.429201	-0.223828	-1.174419	-1.127612	-1.098729
C	-0.113277	-0.271889	-2.017006	-0.495501	1.056330
C	0.088399	0.113816	-1.537828	-2.446404	-0.855572
H	0.125735	0.034597	-0.707259	-0.865991	-2.045046
C	-0.109297	0.111077	-2.386988	-1.818596	1.297496
H	0.149324	0.046763	-2.214124	0.252147	1.821550
C	-0.306463	-0.254060	-2.141511	-2.812359	0.352529
H	0.162621	0.012769	-1.353775	-3.199898	-1.619048
H	0.152960	0.009620	-2.878314	-2.070917	2.235856
H	0.138782	0.019875	-2.430354	-3.843197	0.539925
H	0.067662	-0.409793	1.234404	0.203681	-1.330278
B	0.851968	0.876200	-4.870001	0.314179	-0.314643
C	-0.784508	-0.301660	-4.413867	1.406616	-1.360396
H	0.330689	0.074936	-3.314705	1.354870	-1.422589
H	0.143475	-0.025319	-4.786883	1.215576	-2.374503
H	0.140743	-0.019538	-4.660296	2.436110	-1.073036
C	-0.822403	-0.303368	-5.219320	0.721458	1.173356
H	0.188637	0.002950	-5.050490	-0.086992	1.893587
H	0.177890	0.001638	-4.709485	1.626235	1.522577
H	0.131086	-0.016206	-6.301620	0.939604	1.190119
C	-0.812486	-0.296787	-5.076948	-1.168738	-0.812231
H	0.262541	0.056341	-4.098787	-1.560938	-1.131343
H	0.158432	-0.026954	-5.472470	-1.858133	-0.057870
H	0.140978	-0.016961	-5.722688	-1.203944	-1.701075
C	-0.872100	-0.429112	5.691530	-0.077693	-1.065265
H	0.164598	-0.008617	6.232589	0.769549	-0.626978
H	0.155232	-0.023750	6.359834	-0.948439	-1.062262
H	0.178889	-0.005325	5.464792	0.176102	-2.107475

VIIc

Electronic energy: -1309.062465

Electronic energy + zero-point energy: -1308.603103

Electronic energy + thermal energy correction: -1308.559996

Electronic energy + thermal enthalpy correction: -1308.558814

Electronic energy + thermal free energy correction: -1308.685857

Table S41. Cartesian coordinates, Mulliken and APT charges of all atoms at **VIIc** in the reaction of styrene with PMDSO.

Symbol	X	Y	Z	Mulliken	APT
Si	1.426509	2.333164	1.126096	-0.658177	-0.219405
O	-0.508956	-1.695573	2.939651	-0.929182	-0.110632
Si	0.731450	2.031013	4.334385	-0.155877	0.141260
C	-0.740454	-0.444821	5.052779	0.592687	-1.447064
H	0.181363	-0.006386	5.136316	-0.175333	-2.225704
H	0.181871	0.007773	4.409142	1.390235	-1.835860
H	0.157051	-0.024639	6.052088	1.013991	-1.275170
C	-0.734505	-0.444422	4.195595	1.259310	1.399180
H	0.182465	0.000481	3.972153	0.878211	2.402846
H	0.144833	-0.017173	5.131585	1.830631	1.454629
H	0.179741	-0.001547	3.390366	1.952538	1.126918
C	-0.803559	-0.409054	0.951323	-0.292057	1.651340
H	0.179544	-0.036641	1.896072	-0.461506	2.176255
H	0.222063	0.001204	0.646248	0.750132	1.813971
H	0.112791	-0.028041	0.183495	-0.928110	2.110286
C	-0.883950	-0.391266	1.393780	0.820704	-1.409382
H	0.248830	-0.026640	1.997231	1.578659	-0.890811
H	0.187449	-0.026454	1.981084	0.489712	-2.274997
H	0.129857	-0.008792	0.482150	1.311079	-1.762758
C	-0.844888	-0.889684	-1.019976	-0.638827	-0.516806
H	0.313316	-0.046893	-1.320118	-1.403565	0.216916
C	-0.696599	0.145890	-1.242826	-1.148632	-1.937865
H	0.149590	-0.100327	-2.296926	-1.109799	-2.263384
H	0.160203	-0.029960	-0.663343	-0.568889	-2.672276
H	0.096426	-0.021016	-0.900351	-2.185940	-2.026100
C	-0.958224	0.444262	-1.712720	0.627071	-0.215532
C	0.337526	-0.226806	-2.043950	0.989326	1.110274
C	0.209903	-0.232186	-2.079549	1.555055	-1.214845
C	-0.380624	0.114767	-2.692084	2.178932	1.415780
H	0.201619	0.036562	-1.796518	0.298730	1.912797
C	-0.336186	0.111448	-2.724449	2.752888	-0.911672
H	0.167285	0.034803	-1.872337	1.322751	-2.256462
C	-0.065043	-0.249883	-3.042478	3.080569	0.405615
H	0.131758	0.010963	-2.934400	2.403419	2.453096
H	0.129819	0.004763	-2.991590	3.432176	-1.719155

H	0.147916	0.015633	-3.553718	4.010246	0.641173
H	-0.128094	-0.478440	1.008158	-2.072387	-0.704117
B	0.922092	0.865991	-4.331471	-1.615383	0.302124
C	-0.818285	-0.292379	-4.933842	-0.393164	-0.488735
H	0.163032	0.002779	-4.711514	-0.405334	-1.561976
H	0.132735	-0.008145	-6.031491	-0.473399	-0.389741
H	0.229378	0.022911	-4.645566	0.581684	-0.078878
C	-0.802078	-0.299291	-4.134319	-1.556830	1.870560
H	0.149565	-0.012051	-4.828768	-2.267906	2.344859
H	0.205631	0.022393	-3.126896	-1.903393	2.140985
H	0.184607	-0.006108	-4.288057	-0.567934	2.313530
C	-0.794075	-0.282193	-4.030239	-2.974004	-0.448544
H	0.244128	0.046685	-3.008058	-2.895639	-0.849066
H	0.146633	-0.027219	-4.049286	-3.859855	0.197697
H	0.139427	-0.021472	-4.682747	-3.143414	-1.314378
C	-0.840534	-0.432952	5.633276	-1.362828	0.808386
H	0.170120	-0.004898	5.808434	-2.173882	0.091630
H	0.157746	-0.025136	6.592013	-0.865085	1.003185
H	0.179785	-0.004998	5.286633	-1.816886	1.744369

VIIq

Electronic energy: -1309.056640

Electronic energy + zero-point energy: -1308.595841

Electronic energy + thermal energy correction: -1308.552307

Electronic energy + thermal enthalpy correction: -1308.551125

Electronic energy + thermal free energy correction: -1308.679955

Table S42. Cartesian coordinates, Mulliken and APT charges of all atoms at **VIIq** in the reaction of styrene with PMDSO.

Symbol	X	Y	Z	Mulliken	APT
Si	1.712162	2.382800	-1.545486	-0.847371	-0.385225
O	-0.507450	-1.651919	-3.410901	-0.798329	-0.297061
Si	0.571795	1.922765	-4.628211	0.209027	0.016794
C	-0.743388	-0.422619	-5.132120	0.228187	1.844586
H	0.189618	-0.004306	-5.297365	-0.792216	2.209666
H	0.174223	0.008848	-4.347488	0.679532	2.463316
H	0.174366	-0.024716	-6.058080	0.798670	1.994311

C	-0.744158	-0.423881	-4.278137	2.008743	-0.470274
H	0.194675	0.001145	-4.090492	2.101837	-1.545747
H	0.153283	-0.017856	-5.126214	2.656536	-0.212500
H	0.175950	0.002273	-3.390477	2.388320	0.050948
C	-0.810502	-0.424632	-1.677063	-2.717386	-0.770528
H	0.159294	-0.041763	-2.610146	-2.891039	-1.314610
H	0.149054	-0.027695	-0.834254	-3.105328	-1.353788
H	0.157904	-0.017748	-1.733779	-3.291706	0.164064
C	-0.936780	-0.430340	-1.543669	0.293646	-1.922906
H	0.159670	-0.034911	-2.530559	0.275789	-2.395396
H	0.245971	0.000977	-1.310765	1.332414	-1.653189
H	0.113721	-0.022696	-0.799810	-0.026269	-2.662047
C	-0.571640	-0.897033	0.633232	-1.009298	-0.395627
H	0.158098	-0.038257	0.817689	-1.351309	-1.425229
C	-0.495806	0.156744	1.028101	-2.097741	0.603887
H	0.182850	-0.093063	2.106371	-2.111288	0.830297
H	0.158482	-0.031821	0.506379	-1.983936	1.565857
H	0.133033	-0.029450	0.765373	-3.088843	0.221830
C	-1.183949	0.473246	1.321003	0.277167	-0.179471
C	0.504701	-0.217313	1.480916	1.220796	-1.223148
C	-0.144746	-0.263986	1.831192	0.670229	1.078683
C	-0.205606	0.107153	2.022853	2.481383	-1.009114
H	0.140699	0.033716	1.154263	0.947644	-2.223316
C	-0.098103	0.103872	2.378802	1.935333	1.296600
H	0.165802	0.036887	1.783918	-0.027065	1.911836
C	-0.292876	-0.257906	2.466690	2.863219	0.262041
H	0.148870	0.010889	2.109612	3.173637	-1.844417
H	0.149085	0.011929	2.745847	2.191144	2.289071
H	0.142426	0.021182	2.893529	3.848416	0.429830
H	-0.152342	-0.450338	-5.860546	-0.196887	-0.740411
B	0.769578	0.857456	4.779883	-0.697126	0.083145
C	-0.789275	-0.296729	5.279866	0.678224	-0.501778
H	0.263087	0.069387	4.373354	1.271187	-0.706540
H	0.147612	-0.023802	5.890538	1.271256	0.189387
H	0.143303	-0.022321	5.805409	0.580699	-1.459840
C	-0.773244	-0.295520	4.262654	-1.810492	-0.909589
H	0.146133	-0.018903	4.194557	-2.814388	-0.474655
H	0.296815	0.065681	3.239174	-1.527556	-1.205700
H	0.147213	-0.023472	4.846457	-1.857086	-1.837668
C	-0.808001	-0.300465	4.859088	-0.965154	1.639372
H	0.176898	0.007540	4.140699	-1.705822	2.006592
H	0.130856	-0.015657	5.865719	-1.377164	1.830388
H	0.187937	0.005095	4.773833	-0.052662	2.240477
C	-0.910906	-0.390100	-1.520802	-0.146747	1.403459
H	0.258408	-0.026721	-1.985430	0.849134	1.387943

H	0.191428	-0.028716	-2.153905	-0.778339	2.039199
H	0.093774	-0.012929	-0.539487	-0.038882	1.871690

VIIIa

Electronic energy: -1309.068695

Electronic energy + zero-point energy: -1308.610190

Electronic energy + thermal energy correction: -1308.569039

Electronic energy + thermal enthalpy correction: -1308.567858

Electronic energy + thermal free energy correction: -1308.689358

Table S43. Cartesian coordinates, Mulliken and APT charges of all atoms at **VIIIa** in the reaction of styrene with PMDSO.

Symbol	X	Y	Z	Mulliken	APT
Si	-0.138010	2.175507	-0.471459	-1.158269	-0.435457
O	-0.447479	-1.663155	-2.038421	-0.242389	-0.794789
Si	0.549297	2.003509	-3.332440	0.189861	0.080171
C	-0.717641	-0.429965	-2.869764	0.762487	1.825603
H	0.199653	-0.001741	-2.398761	-0.055934	2.384449
H	0.275290	0.000330	-2.147863	1.587112	1.789950
H	0.137581	-0.025909	-3.752121	1.097563	2.386665
C	-0.642534	-0.427922	-4.267312	1.592353	-0.783466
H	0.179409	-0.009645	-4.611245	1.264952	-1.772137
H	0.173707	-0.026499	-5.146969	1.900519	-0.203268
H	0.183199	0.009065	-3.626797	2.469404	-0.927020
C	-1.005889	-0.422690	-1.451361	-2.796312	-0.200615
H	0.145960	-0.046157	-2.295303	-2.817982	-0.898761
H	0.140698	-0.029586	-0.830184	-3.686164	-0.359747
H	0.195339	-0.011197	-1.865776	-2.862046	0.815090
C	-0.632614	-0.397456	0.172311	-0.871568	-2.206032
H	0.161273	-0.019545	-0.660212	-0.627314	-2.872607
H	0.287648	-0.005121	0.890871	-0.042687	-2.238474
H	0.173772	-0.029927	0.691355	-1.761295	-2.585728
C	-0.491196	-0.791076	1.295448	-2.093240	0.253437
H	0.022650	-0.070760	1.464065	-2.946443	-0.420744
C	-0.306727	0.156289	1.003591	-2.589068	1.670844
H	0.164646	-0.095187	1.916338	-2.837293	2.236013
H	0.128783	-0.028952	0.458713	-1.831817	2.257774

H	0.139582	-0.035158	0.371084	-3.482607	1.648117
C	-0.649322	0.442088	2.456756	-1.185795	0.184902
C	0.835493	-0.197532	3.298285	-1.142756	-0.948413
C	-0.712549	-0.220147	2.774073	-0.293362	1.230374
C	0.054496	0.098498	4.390591	-0.286391	-1.025455
H	0.154349	0.020570	3.081397	-1.811502	-1.779638
C	-0.212611	0.086203	3.869633	0.566439	1.155872
H	0.144887	0.041807	2.150158	-0.278630	2.120504
C	-0.195810	-0.231122	4.693669	0.576148	0.032300
H	0.146977	0.008629	5.013974	-0.291831	-1.917446
H	0.129238	0.013716	4.076802	1.238912	1.986353
H	0.142140	0.017374	5.546725	1.247292	-0.025611
H	0.744740	-0.386746	-0.202953	-0.158691	0.675843
B	0.497821	0.837951	0.674525	2.806143	-0.031233
C	-0.890493	-0.339901	-0.792824	2.960300	-0.578151
H	0.560031	0.110354	-1.131885	1.942349	-0.842960
H	0.203886	-0.026864	-1.515722	3.364745	0.140648
H	0.134808	-0.016568	-0.843940	3.543092	-1.507323
C	-0.758659	-0.298950	1.859895	2.524214	-1.029786
H	0.133362	0.046554	2.153270	1.469741	-0.899726
H	0.140413	-0.026233	1.613451	2.670672	-2.087724
H	0.169448	-0.006292	2.763530	3.097722	-0.783471
C	-0.825456	-0.286883	0.938597	2.845841	1.527841
H	0.193159	-0.020899	1.995149	2.891778	1.814430
H	0.126175	-0.021218	0.390704	3.658728	2.023356
H	0.289112	0.044310	0.529829	1.908278	1.936778
C	-0.914108	-0.441107	-4.592143	-1.215595	0.290830
H	0.172050	0.006618	-4.183504	-2.036350	0.891100
H	0.142317	-0.023675	-5.501683	-0.851055	0.786831
H	0.167709	-0.007590	-4.879838	-1.629281	-0.683562

VIIIb

Electronic energy: -1309.067469

Electronic energy + zero-point energy: -1308.607837

Electronic energy + thermal energy correction: -1308.565014

Electronic energy + thermal enthalpy correction: -1308.563833

Electronic energy + thermal free energy correction: -1308.690771

Table S44. Cartesian coordinates, Mulliken and APT charges of all atoms at **VIIIb** in the reaction of styrene with PMDSO.

Symbol	X	Y	Z	Mulliken	APT
Si	-0.184258	2.143075	0.079525	0.121054	1.138042
O	-0.429735	-1.606659	1.801974	-0.523799	0.913011
Si	0.480236	1.950463	2.471865	-1.532150	-0.166657
C	-0.613762	-0.433589	1.445112	-1.750449	-1.742982
H	0.204127	-0.000528	0.463577	-2.191957	-1.532759
H	0.194986	0.015403	1.263533	-0.786397	-2.230656
H	0.148443	-0.019596	1.964287	-2.404375	-2.456693
C	-0.631316	-0.420857	4.176984	-0.888200	-0.686886
H	0.167114	-0.006558	4.804564	-0.712556	0.195442
H	0.168119	-0.026688	4.696121	-1.601210	-1.340589
H	0.213715	-0.003659	4.090353	0.064448	-1.223437
C	-0.794411	-0.381878	-0.524396	-1.648808	1.565346
H	0.257878	-0.010402	-0.563061	-2.258431	0.652073
H	0.134013	-0.023484	0.205046	-2.115081	2.237855
H	0.170335	-0.025449	-1.518102	-1.700002	2.021924
C	-0.662741	-0.421901	0.619777	1.223868	2.603226
H	0.123427	-0.017751	0.146590	0.869191	3.528636
H	0.146167	-0.037514	1.704642	1.188649	2.737317
H	0.204336	-0.013907	0.310661	2.268000	2.462834
C	-1.286695	-0.764670	-1.873913	0.925037	1.112050
H	0.144858	-0.035165	-1.686374	1.949476	0.760935
C	-0.808173	0.159995	-2.544461	0.973049	2.483830
H	0.157767	-0.106642	-3.619969	1.211091	2.428489
H	0.126234	-0.034136	-2.456523	0.020366	3.026406
H	0.146984	-0.034443	-2.070674	1.734364	3.112761
C	0.262277	0.398273	-2.642713	0.201961	0.076658
C	0.029766	-0.181490	-2.405276	0.436951	-1.296066
C	0.350472	-0.211177	-3.584618	-0.800894	0.383079
C	-0.026410	0.094634	-3.049031	-0.291508	-2.290105
H	0.139786	0.031192	-1.692941	1.211091	-1.572984
C	-0.191475	0.093013	-4.232333	-1.531754	-0.611473
H	0.128554	0.034227	-3.810225	-1.015750	1.424850
C	-0.102902	-0.229956	-3.970072	-1.288725	-1.959172
H	0.146617	0.013273	-2.830522	-0.079079	-3.334904
H	0.130885	0.003166	-4.949652	-2.300004	-0.327938
H	0.133750	0.013207	-4.473899	-1.858754	-2.735617
H	0.380997	-0.461534	0.187060	0.682991	-0.267478
B	0.669360	0.950236	1.413878	2.711075	-0.959125
C	-0.758821	-0.308918	0.183840	3.640881	-0.603680
H	0.161515	-0.012424	-0.734829	3.424805	-1.160790

H	0.161507	0.009957	-0.047796	3.644525	0.467640
H	0.137670	-0.020350	0.483343	4.672526	-0.857460
C	-0.699773	-0.287282	1.526971	2.087151	-2.411318
H	0.244681	-0.017862	2.299498	1.314946	-2.501232
H	0.203103	0.001082	0.578045	1.677550	-2.777394
H	0.132997	-0.024422	1.804884	2.901910	-3.101432
C	-0.821874	-0.330663	2.640115	2.608670	0.026466
H	0.483876	0.111518	2.630659	1.592613	0.454583
H	0.117136	-0.021629	3.601186	2.708906	-0.497760
H	0.133791	-0.033675	2.611635	3.317323	0.862997
C	-0.823437	-0.426915	2.739056	-3.261716	0.564078
H	0.178569	0.004216	1.778522	-3.725220	0.818540
H	0.154817	-0.023642	3.259077	-3.921758	-0.142659
H	0.164922	-0.009513	3.336398	-3.206928	1.482340

VIIIq

Electronic energy: -1309.056085

Electronic energy + zero-point energy: -1308.595290

Electronic energy + thermal energy correction: -1308.551808

Electronic energy + thermal enthalpy correction: -1308.550627

Electronic energy + thermal free energy correction: -1308.677636

Table S45. Cartesian coordinates, Mulliken and APT charges of all atoms at **VIIIq** in the reaction of styrene with PMDSO.

Symbol	X	Y	Z	Mulliken	APT
Si	1.446235	2.295285	0.224346	-0.582566	-0.329030
O	-0.550580	-1.621947	-1.507798	0.250781	-0.346883
Si	0.434705	1.834969	-2.105674	1.732931	-0.112611
C	-0.759111	-0.414354	-2.563240	2.077208	1.697873
H	0.219058	0.001081	-3.043140	1.202365	2.152834
H	0.168759	0.002606	-1.671373	2.309956	2.291839
H	0.175450	-0.023388	-3.257215	2.924649	1.774259
C	-0.858534	-0.411430	-0.973588	3.152587	-0.657883
H	0.174188	0.004027	-0.753406	3.104515	-1.729821
H	0.131542	-0.018002	-1.451831	4.118973	-0.450018
H	0.156538	-0.000035	-0.016586	3.128201	-0.122667
C	-1.051528	-0.427379	-0.655092	-2.258147	-0.611356

H	0.162492	-0.029711	-1.614220	-2.096396	-1.110883
H	0.125049	-0.030790	-0.046211	-2.947868	-1.208541
H	0.189090	-0.011630	-0.862534	-2.748077	0.349580
C	-1.029513	-0.422933	0.697341	0.384032	-1.914117
H	0.117271	-0.028832	-0.199649	0.730384	-2.435867
H	0.259844	0.000774	1.313731	1.259647	-1.669729
H	0.133437	-0.027578	1.281334	-0.239766	-2.601336
C	-0.879341	-0.798665	2.118131	-1.557840	-0.245298
H	0.027179	-0.061393	2.194863	-2.013743	-1.244434
C	-0.434763	0.150955	2.085379	-2.672896	0.805531
H	0.158292	-0.100452	3.083826	-3.093753	1.009529
H	0.151569	-0.026755	1.682805	-2.327428	1.768735
H	0.137748	-0.032231	1.442301	-3.495532	0.477834
C	-0.480335	0.436284	3.270057	-0.637756	-0.071472
C	0.826621	-0.199977	3.865884	0.016202	-1.172047
C	-0.484672	-0.220308	3.837414	-0.357096	1.189026
C	-0.178135	0.097233	4.920144	0.909760	-1.023893
H	0.139997	0.031714	3.485718	-0.193302	-2.169049
C	-0.159474	0.095543	4.892725	0.540774	1.343823
H	0.121068	0.034446	3.446300	-0.858598	2.070522
C	-0.119722	-0.227769	5.443324	1.190263	0.240968
H	0.135405	0.007567	5.342941	1.388185	-1.905265
H	0.132405	0.005901	5.291525	0.727651	2.339191
H	0.128440	0.014279	6.266342	1.890186	0.360077
H	0.009699	-0.427765	-3.376610	1.899344	-0.896617
B	1.002194	0.872079	-4.559811	-0.933376	0.012175
C	-0.943477	-0.319815	-3.785411	-1.692600	1.152653
H	0.431301	0.080948	-2.783330	-1.230067	1.149238
H	0.108943	-0.030536	-4.210050	-1.566820	2.156225
H	0.138126	-0.004606	-3.623234	-2.758284	0.952221
C	-0.811439	-0.310626	-4.317674	-1.293561	-1.500407
H	0.123978	-0.034120	-5.121458	-0.976100	-2.175645
H	0.282990	0.071718	-3.416917	-0.712996	-1.764985
H	0.137434	-0.011603	-4.072669	-2.345374	-1.686849
C	-0.848109	-0.298545	-5.575091	0.220867	0.391247
H	0.158185	-0.004522	-5.782349	0.923088	-0.423048
H	0.140968	-0.009094	-6.526447	-0.280672	0.640947
H	0.173610	0.005405	-5.284623	0.786824	1.284159
C	-0.585745	-0.385160	0.430904	0.153292	1.433314
H	0.283297	-0.023459	0.456399	1.249063	1.357286
H	0.198117	-0.027477	-0.450807	-0.101038	2.034674
H	0.133254	-0.019928	1.329358	-0.152234	1.975004

$IX^{H}a$

Electronic energy: -1309.062962

Electronic energy + zero-point energy: -1308.601623

Electronic energy + thermal energy correction: -1308.560088

Electronic energy + thermal enthalpy correction: -1308.558906

Electronic energy + thermal free energy correction: -1308.678110

Table S46. Cartesian coordinates, Mulliken and APT charges of all atoms at **IX^{H}a** in the reaction of styrene with PMDSO.

Symbol	X	Y	Z	Mulliken	APT
Si	0.554283	2.317230	-0.376906	-0.953965	-0.340832
O	-0.426288	-1.625378	-1.976500	-0.194304	-0.765286
Si	0.431793	2.003195	-3.402601	0.061092	-0.032984
C	-0.572411	-0.433052	-3.215269	0.425758	1.818048
H	0.191437	-0.000928	-2.771314	-0.431887	2.339150
H	0.193240	0.004728	-2.561490	1.287578	1.992896
H	0.162652	-0.019506	-4.188492	0.632678	2.282400
C	-0.749146	-0.431602	-4.339335	1.490928	-0.848530
H	0.165224	-0.003517	-4.399268	1.326271	-1.931495
H	0.161110	-0.025591	-5.364318	1.549745	-0.459542
H	0.188518	0.016022	-3.854329	2.458006	-0.685043
C	-0.873762	-0.419081	-1.326986	-2.545711	0.231973
H	0.150994	-0.049640	-2.052721	-2.800264	-0.549698
H	0.135247	-0.036040	-0.673347	-3.409376	0.401931
H	0.251928	-0.022301	-1.899004	-2.379520	1.155250
C	-0.828438	-0.417775	0.224385	-1.124861	-2.133935
H	0.148232	-0.017136	-0.567018	-0.818245	-2.824792
H	0.232048	0.006235	1.116071	-0.519701	-2.328200
H	0.146586	-0.025983	0.484412	-2.172604	-2.339936
C	-0.434268	-0.744239	1.335982	-1.676293	0.608691
H	0.049080	-0.071737	1.423071	-2.701969	0.219594
C	-0.291999	0.127927	1.021828	-1.717183	2.105886
H	0.160013	-0.089235	1.899956	-1.968411	2.721697
H	0.175560	-0.023424	0.646396	-0.744929	2.461358
H	0.128389	-0.024564	0.242823	-2.455275	2.321603
C	-0.655166	0.354766	2.597020	-0.959137	0.300645
C	0.820944	-0.172439	3.317393	-1.227384	-0.882442
C	-0.622028	-0.202312	3.133493	0.025108	1.149447
C	-0.036057	0.078101	4.475539	-0.533908	-1.212186
H	0.163649	0.026465	2.943406	-1.996628	-1.555491

C	-0.397193	0.069181	4.293065	0.727160	0.821683
H	0.151072	0.041374	2.624075	0.257796	2.079519
C	-0.127893	-0.199048	4.973329	0.459880	-0.363593
H	0.141998	0.008833	4.995315	-0.766559	-2.139367
H	0.143457	0.013139	4.661847	1.495203	1.499007
H	0.146451	0.016493	5.873674	1.010390	-0.623722
H	-0.126519	-0.619216	-0.019317	0.416127	0.329606
B	1.524834	1.131757	0.385629	2.286617	0.001918
C	-0.977033	-0.311021	-1.079400	2.775273	-0.370066
H	0.213263	0.024779	-1.506409	2.228822	-1.215851
H	0.238949	-0.025888	-1.791583	2.737386	0.463294
H	0.092820	-0.048600	-0.992514	3.835619	-0.666199
C	-0.934984	-0.283959	1.422905	2.155151	-1.196867
H	0.209457	-0.001858	2.273052	1.494577	-0.995662
H	0.131375	-0.020559	0.955508	1.849778	-2.140850
H	0.105392	-0.036464	1.843271	3.163528	-1.358601
C	-0.821626	-0.294165	0.905577	2.634823	1.472940
H	0.205349	-0.019992	1.965755	2.422451	1.642695
H	0.127012	-0.053180	0.754937	3.716518	1.627963
H	0.179024	-0.006680	0.320713	2.131239	2.253359
C	-0.838856	-0.442173	-4.585714	-1.419785	-0.170623
H	0.173359	0.010866	-4.256359	-2.273975	0.430390
H	0.147392	-0.027659	-5.592174	-1.136173	0.165031
H	0.171535	-0.005153	-4.660532	-1.755529	-1.212603

IX^{H}b

Electronic energy: -1309.066598

Electronic energy + zero-point energy: -1308.606750

Electronic energy + thermal energy correction: -1308.565371

Electronic energy + thermal enthalpy correction: -1308.564189

Electronic energy + thermal free energy correction: -1308.683949

Table S47. Cartesian coordinates, Mulliken and APT charges of all atoms at **IX^{H}b** in the reaction of styrene with PMDSO.

Symbol	X	Y	Z	Mulliken	APT
Si	0.653877	2.203004	0.115750	0.231820	1.150399
O	-0.448082	-1.596069	1.832143	-0.383811	0.903206

Si	0.504948	1.950105	2.536032	-1.430289	-0.117451
C	-0.636267	-0.433084	1.450973	-1.888294	-1.603356
H	0.201924	-0.001850	0.643563	-2.570353	-1.310987
H	0.210296	0.014299	0.981353	-1.002452	-2.045278
H	0.146479	-0.017781	2.044108	-2.386957	-2.381599
C	-0.620310	-0.418948	4.163340	-0.708624	-0.769176
H	0.166268	-0.010154	4.822237	-0.451988	0.069635
H	0.168609	-0.024929	4.691686	-1.425378	-1.411306
H	0.212951	0.003941	3.996001	0.208201	-1.346214
C	-0.766585	-0.388586	-0.435562	-1.546688	1.619785
H	0.251579	-0.002154	-0.487543	-2.167175	0.714837
H	0.129629	-0.025150	0.320253	-1.989032	2.278761
H	0.166619	-0.021824	-1.415994	-1.612109	2.102538
C	-0.762841	-0.418373	0.624584	1.381610	2.584847
H	0.123065	-0.015140	0.180270	1.029941	3.525498
H	0.141898	-0.038406	1.712156	1.395174	2.700284
H	0.195468	-0.009919	0.270643	2.407999	2.421777
C	-1.319446	-0.761974	-1.861554	0.942524	1.082415
H	0.147998	-0.033861	-1.730218	1.955646	0.680592
C	-0.766192	0.153543	-2.530377	1.028734	2.453330
H	0.159362	-0.108041	-3.612134	1.233691	2.390501
H	0.130742	-0.032346	-2.414213	0.102904	3.035127
H	0.143494	-0.033878	-2.076908	1.829403	3.047335
C	1.121632	0.388886	-2.601266	0.131227	0.087168
C	0.195743	-0.185524	-2.345890	0.277751	-1.293994
C	-0.344730	-0.212896	-3.526937	-0.867824	0.447510
C	-0.071534	0.089509	-2.959135	-0.529070	-2.245809
H	0.120095	0.038723	-1.642431	1.043116	-1.612335
C	-0.298317	0.086611	-4.144660	-1.677131	-0.504811
H	0.107146	0.033216	-3.764046	-1.018639	1.497882
C	-0.249937	-0.221541	-3.865950	-1.520035	-1.861559
H	0.152772	0.015210	-2.726817	-0.383471	-3.299042
H	0.134383	0.003107	-4.850595	-2.439437	-0.180002
H	0.133976	0.014497	-4.344846	-2.152741	-2.604549
H	-0.528907	-0.585622	0.230060	0.844581	-0.251586
B	0.971625	1.090341	1.133077	2.559156	-1.076905
C	-0.800621	-0.313578	-0.099941	3.535449	-0.862229
H	0.160622	-0.012896	-1.026766	3.237353	-1.365870
H	0.139765	0.000406	-0.320623	3.723958	0.194897
H	0.130210	-0.030225	0.200798	4.506677	-1.292962
C	-0.613636	-0.296481	1.305299	1.827797	-2.477887
H	0.258289	-0.015354	2.053641	1.027199	-2.462525
H	0.175461	-0.005808	0.371827	1.421687	-2.885665
H	0.132999	-0.040357	1.664442	2.582971	-3.197880
C	-0.859434	-0.325067	2.402071	2.692429	-0.135858

H	0.421693	0.083821	2.650126	1.714751	0.298378
H	0.107505	-0.034650	3.277557	3.004299	-0.726956
H	0.133055	-0.040853	2.275036	3.403730	0.689705
C	-0.854403	-0.427584	2.984225	-3.054976	0.750665
H	0.175027	0.003123	2.082323	-3.559952	1.116635
H	0.150977	-0.023649	3.505122	-3.741245	0.069855
H	0.163062	-0.007792	3.637148	-2.868345	1.611864

IXq

Electronic energy: -1309.052895

Electronic energy + zero-point energy: -1308.590127

Electronic energy + thermal energy correction: -1308.549679

Electronic energy + thermal enthalpy correction: -1308.548497

Electronic energy + thermal free energy correction: -1308.665969

Table S48. Cartesian coordinates, Mulliken and APT charges of all atoms at **IXq** in the reaction of styrene with PMDSO.

Symbol	X	Y	Z	Mulliken	APT
Si	0.980222	2.171273	-0.046168	-0.751393	0.233589
O	-0.664780	-1.668915	1.777216	0.238126	0.233548
Si	0.568165	1.783316	1.906634	1.859396	0.129276
C	-0.735030	-0.404064	2.196897	2.467923	-1.651428
H	0.189372	0.000522	2.741128	1.723257	-2.243357
H	0.171198	-0.005820	1.241724	2.657140	-2.156225
H	0.175134	-0.021858	2.776301	3.400260	-1.659620
C	-0.756033	-0.394815	0.440602	2.890368	0.765030
H	0.175947	-0.000960	0.339729	2.826253	1.853617
H	0.137939	-0.026967	0.640128	3.940699	0.509626
H	0.177375	-0.004211	-0.523950	2.616596	0.322932
C	-1.060666	-0.425214	0.850766	-2.420690	0.307889
H	0.166650	0.005536	1.858997	-2.303715	0.701236
H	0.115300	-0.026786	0.294754	-3.111198	0.954794
H	0.167100	-0.007760	0.924663	-2.879743	-0.685704
C	-0.817516	-0.418435	-0.332943	-0.007249	1.972794
H	0.118760	-0.017985	0.611249	0.314185	2.420387
H	0.270019	0.006778	-1.018385	0.846912	1.955938
H	0.139444	-0.030483	-0.784023	-0.773391	2.617037

C	-1.014367	-0.714653	-1.940107	-1.652678	0.203151
H	0.029300	-0.059100	-2.012088	-2.120303	1.196858
C	-0.480976	0.135301	-2.022321	-2.761484	-0.852928
H	0.161134	-0.089281	-3.052228	-3.123412	-1.002032
H	0.128258	-0.026246	-1.651840	-2.438314	-1.835480
H	0.150515	-0.030173	-1.409776	-3.619013	-0.558307
C	-0.494012	0.380170	-3.044821	-0.660637	0.088309
C	1.125793	-0.184777	-3.548565	0.008989	1.222577
C	-0.395259	-0.203996	-3.637548	-0.322742	-1.144007
C	-0.227422	0.080774	-4.545103	0.974776	1.130886
H	0.173478	0.031871	-3.142185	-0.245381	2.198978
C	-0.272882	0.079468	-4.635153	0.646034	-1.242340
H	0.127032	0.036565	-3.311917	-0.831490	-2.047695
C	-0.076406	-0.205955	-5.096144	1.310481	-0.107568
H	0.135704	0.011767	-4.898652	1.466191	2.035080
H	0.135974	0.008818	-5.058268	0.878644	-2.217616
H	0.138896	0.019655	-5.873660	2.066104	-0.184203
H	-0.048671	-0.416950	3.046387	2.366176	0.957893
B	1.208267	1.091097	3.951108	-0.731729	0.022311
C	-0.892891	-0.260690	3.607371	-1.598923	-1.270729
H	0.279186	0.016842	2.729353	-1.170898	-1.770236
H	0.108149	-0.050024	4.436921	-1.555198	-1.992637
H	0.143002	-0.029159	3.388603	-2.653483	-1.068972
C	-0.788971	-0.278459	4.006592	-1.329784	1.499772
H	0.130078	-0.046887	5.003596	-1.170951	1.937572
H	0.252979	0.022213	3.293324	-0.780969	2.127800
H	0.135365	-0.039374	3.771579	-2.397645	1.581010
C	-0.938669	-0.313268	4.872052	0.546325	-0.224460
H	0.124330	-0.004141	4.970828	1.213692	0.637240
H	0.123699	-0.039725	5.873177	0.122450	-0.417600
H	0.165070	-0.004533	4.618161	1.146160	-1.106056
C	-0.529366	-0.399530	-0.303250	0.081305	-1.473028
H	0.333098	-0.001207	-0.610219	1.128875	-1.357943
H	0.187949	-0.010691	0.635375	0.075140	-2.035479
H	0.144038	-0.018876	-1.078521	-0.404120	-2.072399

X^{O}a

Electronic energy: -1309.103731

Electronic energy + zero-point energy: -1308.642999

Electronic energy + thermal energy correction: -1308.600932

Electronic energy + thermal enthalpy correction: -1308.599750

Electronic energy + thermal free energy correction: -1308.722330

Table S49. Cartesian coordinates, Mulliken and APT charges of all atoms at $\mathbf{X^{O}a}$ in the reaction of styrene with PMDSO.

Symbol	X	Y	Z	Mulliken	APT
Si	-0.098727	1.510206	-0.663887	1.696666	-0.181908
O	-0.582410	-1.490831	2.169154	-0.202136	-0.411809
Si	0.745255	1.954278	1.737636	-1.680124	0.090079
C	-0.586879	-0.435723	1.591709	-1.834604	1.974108
H	0.190610	0.000414	2.567971	-1.691790	2.452152
H	0.172272	-0.000012	0.911159	-1.072766	2.373068
H	0.156940	-0.022317	1.210169	-2.824034	2.260399
C	-0.965854	-0.410627	0.048485	-2.123234	-0.645945
H	0.164583	-0.007827	0.078346	-2.074734	-1.741798
H	0.144368	-0.017032	-0.242680	-3.143778	-0.362809
H	0.317705	-0.001871	-0.747090	-1.449692	-0.303944
C	-0.802803	-0.417391	-0.026673	3.458681	0.046543
H	0.212300	0.022712	1.024224	3.520027	-0.253036
H	0.157319	-0.010832	-0.604172	4.156783	-0.573724
H	0.164140	0.009270	-0.094907	3.784739	1.090198
C	-0.810112	-0.437288	-0.300256	1.086159	-1.915852
H	0.309842	0.093933	0.751706	0.781139	-1.955233
H	0.217271	0.019500	-0.916982	0.220302	-2.179173
H	0.123718	-0.012355	-0.480643	1.883950	-2.647709
C	-0.485401	-0.417094	-2.571798	1.737671	0.101474
H	0.082920	-0.045564	-3.014555	2.298207	-0.735027
C	-0.423119	0.084818	-2.898512	2.464688	1.412979
H	0.181700	-0.050063	-3.957918	2.359737	1.682193
H	0.154912	-0.001725	-2.303109	2.082796	2.251444
H	0.164824	-0.021160	-2.671891	3.532500	1.323792
C	-1.142357	0.177250	-3.087383	0.325102	0.041206
C	0.493301	-0.124953	-3.548060	-0.215849	-1.167926
C	0.187199	-0.149838	-3.055772	-0.520457	1.160056
C	0.109478	0.021004	-3.949754	-1.545513	-1.262837
H	0.171026	0.033094	-3.572930	0.419132	-2.051818
C	-0.183388	0.018882	-3.461799	-1.850368	1.070851
H	0.188999	0.052939	-2.684612	-0.141385	2.108757
C	-0.187550	-0.117575	-3.908881	-2.372773	-0.141159
H	0.162536	0.026362	-4.290451	-1.937948	-2.217713
H	0.162574	0.028708	-3.411680	-2.485492	1.951569

H	0.157908	0.032481	-4.211953	-3.413595	-0.213483
H	0.815611	-0.314725	-0.169621	0.803858	0.894034
B	1.133314	1.195692	3.336289	0.717361	-0.041897
C	-1.037096	-0.217621	4.722452	-0.156125	0.122394
H	0.142502	-0.074406	4.958578	-0.740512	-0.782199
H	0.106282	-0.070156	5.584342	0.503272	0.313790
H	0.142707	-0.077107	4.684917	-0.868485	0.965498
C	-0.889277	-0.194969	2.997096	1.488821	1.373428
H	0.090687	-0.079216	3.800014	2.193673	1.645836
H	0.229078	-0.088263	2.064357	2.073364	1.332429
H	0.158389	-0.075822	2.890079	0.790053	2.219326
C	-0.821929	-0.182203	3.487628	1.792131	-1.274579
H	0.179448	-0.089260	2.562130	2.366639	-1.447714
H	0.109714	-0.084429	4.288243	2.526361	-1.089770
H	0.123384	-0.081614	3.723255	1.285718	-2.225110
C	-0.959991	-0.435532	2.917223	-3.055202	-0.477323
H	0.166815	0.002751	3.895229	-2.971693	0.008588
H	0.137207	-0.022935	2.499735	-4.045010	-0.247123
H	0.148054	-0.003959	3.079339	-2.997410	-1.560631

X^{H}a

Electronic energy: -1309.116958

Electronic energy + zero-point energy: -1308.654611

Electronic energy + thermal energy correction: -1308.612802

Electronic energy + thermal enthalpy correction: -1308.611620

Electronic energy + thermal free energy correction: -1308.735047

Table S50. Cartesian coordinates, Mulliken and APT charges of all atoms at **X^{H}a** in the reaction of styrene with PMDSO.

Symbol	X	Y	Z	Mulliken	APT
Si	0.704409	2.054820	0.053293	-1.652780	-0.370844
O	-0.318011	-1.664657	-1.536234	-1.628436	0.097779
Si	0.989138	2.063073	-2.954212	-0.755335	0.069890
C	-0.975345	-0.425657	-4.328921	-2.046659	0.174285
H	0.167622	-0.003813	-4.280542	-2.741510	-0.672272
H	0.159536	-0.000962	-4.244412	-2.633552	1.096482
H	0.153052	-0.006125	-5.317333	-1.570697	0.165805

C	-0.708488	-0.462798	-3.078518	0.368441	1.561698
H	0.239906	0.069628	-2.301728	1.140039	1.529205
H	0.136774	0.000698	-4.052927	0.873205	1.574025
H	0.157512	-0.003896	-2.979018	-0.201927	2.493252
C	-0.819013	-0.431037	0.468977	-3.445024	-0.791779
H	0.170405	-0.005662	-0.089262	-3.781918	-1.673003
H	0.174780	0.008561	1.539365	-3.550453	-1.009552
H	0.170080	-0.002626	0.222359	-4.111920	0.042472
C	-0.634824	-0.471474	0.349263	-0.606362	-1.896055
H	0.141540	-0.002855	-0.343715	-0.911803	-2.690478
H	0.306334	0.080682	0.199163	0.460531	-1.696572
H	0.183594	0.004389	1.374115	-0.747544	-2.261005
C	-1.216706	-0.403206	1.144443	-1.163851	1.120033
H	0.109152	-0.055453	1.373564	-2.113175	1.628849
C	-0.251958	0.063627	0.415122	-0.245901	2.114049
H	0.147014	-0.058962	1.074627	0.019372	2.950315
H	0.248011	0.064121	0.076357	0.681413	1.641411
H	0.171539	-0.024971	-0.469777	-0.750426	2.515901
C	0.081958	0.158003	2.445400	-0.589007	0.618886
C	0.388192	-0.110039	3.597341	-1.379372	0.522864
C	-0.264937	-0.132110	2.518998	0.744704	0.189318
C	-0.026209	-0.002577	4.789609	-0.861047	0.015967
H	0.137759	0.021245	3.557393	-2.416475	0.853807
C	-0.086622	0.012767	3.708182	1.264535	-0.313905
H	-0.304091	0.122568	1.631117	1.373671	0.228644
C	0.143291	-0.100332	4.850426	0.466178	-0.403658
H	0.141106	0.013936	5.671226	-1.495240	-0.044532
H	0.135799	0.038713	3.733483	2.299689	-0.644931
H	0.144919	0.020961	5.777252	0.875054	-0.797927
H	-1.813527	-0.444866	-0.730415	1.600925	-0.111312
B	2.124129	0.692559	-0.684921	2.851345	-0.124885
C	-0.939613	-0.149459	-2.202117	3.448766	-0.370518
H	0.107925	-0.081791	-2.612308	3.201787	-1.363762
H	0.129315	-0.081023	-2.942030	3.104929	0.371887
H	0.138437	-0.107180	-2.191669	4.551361	-0.308188
C	-0.847734	-0.137568	0.291654	3.338762	-1.360381
H	0.240782	-0.094388	1.340112	3.005202	-1.275236
H	0.090902	-0.082740	-0.066239	2.988469	-2.343698
H	0.129622	-0.104038	0.324036	4.441094	-1.417739
C	-0.824850	-0.148036	-0.111383	3.380837	1.327658
H	0.206665	-0.095056	0.914121	3.045823	1.561350
H	0.141263	-0.108256	-0.085910	4.484307	1.351955
H	0.120351	-0.085455	-0.741280	3.068777	2.179152
C	-0.672822	-0.458577	-3.118678	0.182559	-1.542865
H	0.169397	-0.010147	-3.110420	-0.501234	-2.400885

H	0.159884	0.006885	-4.059130	0.747163	-1.563579
H	0.242658	0.060556	-2.301611	0.904083	-1.647874
Si	0.704409	2.054820	0.053293	-1.652780	-0.370844

X^O b

Electronic energy: -1309.104533

Electronic energy + zero-point energy: -1308.642264

Electronic energy + thermal energy correction: -1308.600899

Electronic energy + thermal enthalpy correction: -1308.599717

Electronic energy + thermal free energy correction: -1308.721149

Table S51. Cartesian coordinates, Mulliken and APT charges of all atoms at **X^O b** in the reaction of styrene with PMDSO.

Symbol	X	Y	Z	Mulliken	APT
Si	1.444029	1.497911	-0.911358	0.473080	-0.830709
O	-0.616934	-1.500250	2.617424	0.198845	-0.174659
Si	-0.037786	1.955668	2.747489	-1.409505	-0.327905
C	-0.467418	-0.422407	1.576851	-2.343877	0.835495
H	0.226097	0.006312	1.761187	-2.050205	1.876164
H	0.253005	-0.010100	0.526683	-2.125002	0.607424
H	0.152863	-0.025537	1.720183	-3.429677	0.753837
C	-0.590964	-0.405812	2.288603	-1.880595	-2.104853
H	0.172841	-0.013168	3.010245	-1.447701	-2.808379
H	0.171227	-0.022006	2.285220	-2.969468	-2.245119
H	0.183648	-0.001719	1.294892	-1.502307	-2.371345
C	-1.092432	-0.440698	-0.572223	0.579223	1.009105
H	0.172641	0.006252	-1.147807	-0.167211	1.568142
H	0.205045	0.090446	0.497935	0.405945	1.169425
H	0.170487	0.019934	-0.808787	1.572280	1.410327
C	-1.030295	-0.454296	0.227404	1.550942	-1.858212
H	0.143954	-0.008533	0.000006	1.474050	-2.928711
H	0.188145	0.019153	0.152986	2.602529	-1.557218
H	0.285553	0.095283	1.260804	1.232920	-1.670224
C	-0.757555	-0.423860	-2.737050	0.993657	-1.172058
H	0.117266	-0.042761	-2.932896	0.739714	-2.223727
C	-0.556867	0.072366	-2.905020	2.509068	-0.999222
H	0.179619	-0.047737	-3.961053	2.806426	-1.048496

H	0.177116	-0.001353	-2.504728	2.865967	-0.042434
H	0.151081	-0.013217	-2.363074	3.041909	-1.786636
C	-0.845539	0.198874	-3.661117	0.168409	-0.318248
C	0.583765	-0.129928	-4.058271	-1.110205	-0.738358
C	-0.045679	-0.141537	-4.114154	0.609152	0.932832
C	-0.053814	0.028094	-4.875829	-1.913310	0.051687
H	0.176733	0.035887	-3.708612	-1.478362	-1.701139
C	-0.019054	0.028071	-4.932029	-0.192605	1.728552
H	0.141679	0.048645	-3.816753	1.589117	1.296611
C	-0.119875	-0.122176	-5.319867	-1.457813	1.293387
H	0.147777	0.024162	-5.165681	-2.899483	-0.302279
H	0.159585	0.024253	-5.264188	0.175079	2.696177
H	0.141691	0.028921	-5.955748	-2.082962	1.914147
H	0.070662	-0.369816	-0.826283	-0.947472	-1.279401
B	1.427396	1.195354	3.298046	1.190220	0.783273
C	-0.961024	-0.218782	4.878512	1.354205	0.356027
H	0.132877	-0.074591	4.980337	1.573558	-0.719640
H	0.122944	-0.074607	5.356044	2.185561	0.899906
H	0.143340	-0.073334	5.484063	0.457105	0.562134
C	-0.924959	-0.208532	3.181592	0.644423	2.334560
H	0.157541	-0.081675	2.141387	0.538741	2.685260
H	0.156475	-0.082902	3.666155	-0.336918	2.479587
H	0.113593	-0.075701	3.677945	1.338952	3.031360
C	-0.933543	-0.191611	2.532063	2.632857	0.611586
H	0.219532	-0.086816	1.451933	2.577029	0.823950
H	0.088946	-0.079885	2.952071	3.388293	1.294860
H	0.116638	-0.072060	2.636634	3.032227	-0.410694
C	-0.912533	-0.440520	4.481778	-2.112205	-0.024849
H	0.174304	0.007700	4.813925	-1.939482	1.005154
H	0.131407	-0.022196	4.486934	-3.195185	-0.209385
H	0.164771	-0.003164	5.215110	-1.645839	-0.692733

$X^{H}b$

Electronic energy: -1309.117333

Electronic energy + zero-point energy: -1308.656332

Electronic energy + thermal energy correction: -1308.615034

Electronic energy + thermal enthalpy correction: -1308.613852

Electronic energy + thermal free energy correction: -1308.734757

Table S52. Cartesian coordinates, Mulliken and APT charges of all atoms at $\mathbf{X^{H}b}$ in the reaction of styrene with PMDSO.

Symbol	X	Y	Z	Mulliken	APT
Si	0.365475	2.033552	0.217989	0.565629	1.675113
O	-0.223242	-1.605436	-0.190583	1.463736	0.351770
Si	0.945385	2.053692	-1.385532	1.952269	-0.689114
C	-0.673376	-0.457754	-1.204274	1.146290	-2.368572
H	0.190607	0.006965	-0.190883	1.293339	-2.760207
H	0.280609	0.070995	-1.402212	0.071184	-2.301888
H	0.141258	-0.011418	-1.915045	1.585919	-3.080350
C	-0.670119	-0.457990	-3.077864	1.636734	0.055124
H	0.183723	-0.008113	-3.197800	2.169694	1.006617
H	0.156447	-0.000767	-3.870643	1.976080	-0.623715
H	0.288488	0.069943	-3.218730	0.564841	0.231584
C	-0.665010	-0.436835	1.316199	1.683182	2.731077
H	0.195511	0.010791	2.269782	1.877259	2.224690
H	0.182756	-0.002238	0.824533	2.648813	2.897029
H	0.179065	-0.000407	1.532258	1.238565	3.709911
C	-0.507569	-0.467365	-1.289425	0.034045	2.658243
H	0.151557	0.008280	-0.988258	-0.508840	3.563251
H	0.173121	-0.017329	-1.893931	0.895491	2.966462
H	0.320379	0.066112	-1.911810	-0.632348	2.049491
C	-1.413676	-0.480996	1.217687	-0.970854	1.176773
H	0.014961	0.052196	0.454975	-1.694592	0.868324
C	-0.485149	0.089012	1.976908	-1.546582	2.378523
H	0.191360	-0.040279	2.545309	-2.439669	2.089872
H	0.159130	-0.021272	2.681689	-0.829709	2.816864
H	0.151495	-0.015867	1.276490	-1.840762	3.169144
C	0.835147	0.184853	2.074578	-0.683281	-0.026344
C	0.064675	-0.122714	1.508930	-0.806348	-1.305240
C	-0.130729	-0.127908	3.408733	-0.268860	0.068320
C	-0.098699	0.014331	2.251893	-0.522176	-2.445910
H	0.011707	0.105672	0.472226	-1.132753	-1.383908
C	-0.296764	0.009240	4.155155	0.018629	-1.076186
H	0.115154	0.032689	3.876636	-0.169945	1.045337
C	-0.027877	-0.114689	3.580818	-0.104814	-2.338988
H	0.158653	0.028845	1.788714	-0.630035	-3.424103
H	0.130838	0.012518	5.189876	0.339399	-0.977131
H	0.143257	0.018672	4.161457	0.118142	-3.230641
H	-1.570129	-0.432800	-1.521196	-1.085287	-0.255396
B	1.466283	0.692330	-2.094765	-2.182705	-0.432292
C	-0.716954	-0.156171	-1.334352	-3.349120	0.452627
H	0.166745	-0.090852	-0.304565	-3.558681	0.113039

H	0.167879	-0.081110	-1.272943	-3.116748	1.531425
H	0.137679	-0.108865	-1.880428	-4.305238	0.374612
C	-0.786906	-0.144146	-2.038684	-2.573347	-2.032406
H	0.116894	-0.091783	-2.606728	-1.875233	-2.670397
H	0.174242	-0.086722	-1.014641	-2.618781	-2.443063
H	0.144510	-0.105261	-2.479827	-3.571432	-2.201980
C	-0.767224	-0.156930	-3.665712	-2.057882	0.050848
H	0.124565	-0.087715	-4.248441	-1.340138	-0.550867
H	0.140963	-0.108509	-4.177158	-3.031310	-0.050604
H	0.129903	-0.082370	-3.786263	-1.757606	1.106830
C	-0.943108	-0.424445	-1.143439	3.815684	-0.890160
H	0.160453	-0.002117	-0.148120	4.034431	-1.294288
H	0.157144	-0.005812	-1.888252	4.242193	-1.573507
H	0.158508	-0.005703	-1.234893	4.329014	0.074498

X^O^c

Electronic energy: -1309.100055

Electronic energy + zero-point energy: -1308.638582

Electronic energy + thermal energy correction: -1308.597883

Electronic energy + thermal enthalpy correction: -1308.596701

Electronic energy + thermal free energy correction: -1308.716719

Table S53. Cartesian coordinates, Mulliken and APT charges of all atoms at **X^{O}c** in the reaction of styrene with PMDSO.

Symbol	X	Y	Z	Mulliken	APT
Si	1.452899	1.488139	-0.836233	0.165405	0.665252
O	-0.535115	-1.494845	2.602776	0.223534	0.247836
Si	-0.313340	1.966731	3.059199	-1.279977	-0.130544
C	-0.550230	-0.409417	2.637879	-2.425990	1.321907
H	0.170126	-0.016631	3.390727	-2.309906	2.111465
H	0.190014	-0.007576	1.667959	-2.163397	1.758886
H	0.166015	-0.017559	2.612504	-3.483961	1.029675
C	-0.450001	-0.422056	2.164516	-1.915367	-1.681589
H	0.225976	0.000610	2.401119	-1.263989	-2.532579
H	0.156422	-0.025425	2.479304	-2.936369	-1.935535
H	0.229271	-0.018173	1.074000	-1.916708	-1.565920
C	-1.216388	-0.445666	-0.400355	0.706014	-1.076071

H	0.172584	0.087361	0.675156	0.557618	-1.218917
H	0.172030	0.000392	-0.955412	0.123033	-1.821194
H	0.168806	0.017708	-0.608547	1.769292	-1.239320
C	-0.930693	-0.395922	-1.044293	-1.712368	0.728121
H	0.191626	0.004873	-1.853418	-2.022694	0.055582
H	0.260426	0.022679	-0.127141	-2.217545	0.409058
H	0.178347	0.002300	-1.283578	-2.067079	1.737757
C	-0.428580	-0.426662	-2.532520	0.929771	1.178592
H	0.123851	-0.030774	-2.418951	2.011666	1.018427
C	-0.473869	0.080347	-2.796489	0.687273	2.669741
H	0.183767	-0.052708	-3.791823	1.041934	2.969340
H	0.170453	-0.008371	-2.731997	-0.375024	2.935026
H	0.162897	-0.007824	-2.048424	1.210912	3.272941
C	-0.984877	0.192649	-3.626426	0.440810	0.269118
C	0.588010	-0.127027	-3.851957	1.064740	-0.968242
C	-0.102024	-0.138547	-4.424651	-0.667050	0.588739
C	-0.053465	0.027815	-4.828567	0.605030	-1.846870
H	0.161237	0.038749	-3.241473	1.922865	-1.242177
C	-0.132883	0.026252	-5.404101	-1.131586	-0.288687
H	0.140668	0.046006	-4.277022	-1.179874	1.535598
C	-0.086505	-0.121606	-5.613810	-0.498806	-1.511807
H	0.152521	0.026467	-4.976792	1.110286	-2.797904
H	0.149343	0.021834	-6.004647	-1.994863	-0.012559
H	0.146330	0.028109	-6.376145	-0.860659	-2.196264
H	0.057257	-0.334734	0.122291	0.624005	1.696495
B	1.593310	1.207827	3.178373	1.609375	-0.077008
C	-0.914529	-0.205899	3.312852	1.783916	-1.710767
H	0.151390	-0.086186	3.984926	1.033334	-2.162685
H	0.152362	-0.079732	2.346711	1.701772	-2.236051
H	0.117232	-0.078182	3.730527	2.769680	-1.972684
C	-0.996963	-0.212855	4.654341	1.771283	0.634806
H	0.133558	-0.076172	4.614860	1.508236	1.704910
H	0.143190	-0.076198	5.436114	1.145456	0.175376
H	0.118943	-0.077179	5.013056	2.811772	0.573796
C	-0.946217	-0.194959	2.143645	2.717306	0.552510
H	0.125924	-0.070413	2.023315	2.581348	1.639517
H	0.089515	-0.083921	2.507996	3.744754	0.391513
H	0.218101	-0.088581	1.135471	2.665137	0.110747
C	-0.957757	-0.444869	4.907032	-1.526471	-0.469905
H	0.163988	0.000981	5.504950	-1.250545	0.405837
H	0.127835	-0.022567	5.109363	-2.579968	-0.707692
H	0.167211	0.011411	5.246846	-0.912686	-1.311620

$X^{H}c$

Electronic energy: -1309.118162

Electronic energy + zero-point energy: -1308.656327

Electronic energy + thermal energy correction: -1308.614132

Electronic energy + thermal enthalpy correction: -1308.612950

Electronic energy + thermal free energy correction: -1308.738695

Table S54. Cartesian coordinates, Mulliken and APT charges of all atoms at $\mathbf{X^{H}c}$ in the reaction of styrene with PMDSO.

Symbol	X	Y	Z	Mulliken	APT
Si	1.502806	2.078641	-0.256548	-1.008088	-0.607067
O	-0.309787	-1.702887	1.192895	-1.530180	0.002513
Si	0.774768	2.116127	2.826242	-1.259020	0.124625
C	-0.936917	-0.431390	3.597970	-2.980550	0.256294
H	0.159578	-0.003242	3.214787	-3.513239	1.134683
H	0.168780	-0.005994	3.365747	-3.583645	-0.629416
H	0.153974	-0.008067	4.689651	-2.918942	0.347853
C	-0.584482	-0.466376	3.491783	-0.431477	-1.420571
H	0.233355	0.066536	3.068784	0.572359	-1.525319
H	0.160359	-0.001739	4.584013	-0.337052	-1.370186
H	0.184663	-0.007487	3.241545	-1.018888	-2.312882
C	-0.559190	-0.457618	-0.055534	-0.231462	-2.302504
H	0.175091	-0.011336	0.493837	-0.899739	-2.976864
H	0.177846	0.014136	-1.034901	-0.029013	-2.753335
H	0.241753	0.057501	0.490169	0.714494	-2.216438
C	-0.942669	-0.428802	-1.310517	-2.567105	-0.743161
H	0.185924	0.004794	-2.281949	-2.359886	-1.206904
H	0.151269	-0.003461	-0.790113	-3.322202	-1.344117
H	0.174276	0.009280	-1.499658	-2.999442	0.247234
C	-0.855621	-0.492079	-1.103051	0.198842	0.601317
H	-0.185215	0.053397	-0.749904	1.194972	0.308631
C	-0.683063	0.070024	-0.629045	-0.065214	2.034311
H	0.175708	-0.043250	-1.201349	0.532948	2.755265
H	0.121323	-0.025645	-0.711957	-1.121836	2.323381
H	0.140956	0.019443	0.423753	0.220849	2.117878
C	-0.102704	0.224881	-2.591963	0.138375	0.397786
C	-0.282306	-0.143424	-3.182775	0.856319	-0.654759
C	-0.078652	-0.150785	-3.430740	-0.655354	1.192626
C	0.082393	0.038182	-4.548096	0.771624	-0.912769
H	0.067659	0.047252	-2.551313	1.496579	-1.268545

C	0.088069	0.026449	-4.799199	-0.741836	0.938531
H	0.145843	0.045421	-3.005052	-1.215850	2.021368
C	-0.185758	-0.131207	-5.366786	-0.033455	-0.118984
H	0.165606	0.022797	-4.976916	1.342525	-1.732987
H	0.147247	0.015884	-5.424716	-1.366931	1.571704
H	0.133600	0.022074	-6.433334	-0.099795	-0.317334
H	-1.612846	-0.418694	1.437248	1.329154	-0.083296
B	1.830651	0.667704	1.531747	2.568696	0.020052
C	-0.867443	-0.131642	0.572931	3.276441	-1.119734
H	0.248431	-0.105448	-0.493783	2.998615	-1.041437
H	0.128955	-0.102966	0.606839	4.376343	-1.030249
H	0.099593	-0.084865	0.883197	3.041691	-2.152447
C	-0.921464	-0.144749	3.101499	3.011096	-0.220609
H	0.139730	-0.082351	3.811005	2.509404	0.460191
H	0.106303	-0.088276	3.458850	2.815605	-1.245824
H	0.137088	-0.104191	3.227931	4.095293	-0.053919
C	-0.707353	-0.136241	1.046868	3.026202	1.527911
H	0.147484	-0.085354	1.639283	2.571005	2.340742
H	0.137813	-0.108185	1.152398	4.118887	1.646929
H	0.213503	-0.091006	-0.010005	2.794596	1.745075
C	-0.613374	-0.462075	3.241391	-0.312793	1.686761
H	0.173843	-0.002907	2.847161	-0.832036	2.569172
H	0.157077	-0.009296	4.328935	-0.221479	1.803751
H	0.195528	0.072515	2.813776	0.694213	1.646805

Xq

Electronic energy: -1309.104701

Electronic energy + zero-point energy: -1308.644076

Electronic energy + thermal energy correction: -1308.603019

Electronic energy + thermal enthalpy correction: -1308.601837

Electronic energy + thermal free energy correction: -1308.722651

Table S55. Cartesian coordinates, Mulliken and APT charges of all atoms at **Xq** in the reaction of styrene with PMDSO.

Symbol	X	Y	Z	Mulliken	APT
Si	0.587267	1.613020	-0.692556	1.433614	-0.109620
O	-0.595281	-1.471308	2.564790	-0.431742	-0.443250

Si	0.317376	1.846244	2.474100	-2.005757	-0.076098
C	-0.655060	-0.417284	2.601048	-2.367400	1.779030
H	0.202653	0.008822	3.552321	-1.999838	2.179371
H	0.168524	0.005370	1.793093	-1.869610	2.329102
H	0.163399	-0.023166	2.537404	-3.445712	1.976051
C	-0.739451	-0.380246	0.812814	-2.682681	-0.678608
H	0.186598	-0.001043	0.706645	-2.541229	-1.760448
H	0.154697	-0.022308	0.722900	-3.755404	-0.463963
H	0.330353	-0.012118	-0.026738	-2.169227	-0.194094
C	-0.777024	-0.434381	0.056857	3.163602	-0.018544
H	0.201638	0.022194	1.133484	3.115668	-0.212984
H	0.164889	-0.008729	-0.394954	3.830210	-0.764295
H	0.157506	0.002333	-0.083496	3.610601	0.972575
C	-0.716107	-0.464537	-0.241349	0.609850	-1.731827
H	0.362131	0.105133	0.827238	0.358195	-1.704839
H	0.230006	0.016237	-0.803667	-0.318363	-1.884539
H	0.137490	-0.008738	-0.434707	1.277034	-2.581716
C	-0.645066	-0.439700	-2.610801	1.671602	-0.038134
H	0.085979	-0.044633	-2.875526	2.215208	-0.957468
C	-0.433005	0.083655	-3.008512	2.533948	1.167845
H	0.177548	-0.054384	-4.098862	2.579717	1.290801
H	0.176718	-0.003106	-2.586429	2.151033	2.104735
H	0.162304	-0.016389	-2.635636	3.555952	1.045072
C	-0.901127	0.187368	-3.297473	0.334289	-0.081928
C	0.763480	-0.130426	-3.623894	-0.259897	-1.310383
C	-0.045665	-0.138840	-3.589925	-0.388389	1.083732
C	-0.146917	0.024837	-4.208368	-1.521554	-1.375439
H	0.198404	0.036289	-3.400593	0.278975	-2.229450
C	-0.134778	0.025553	-4.176100	-1.651802	1.023973
H	0.168908	0.047018	-3.344547	0.037181	2.053516
C	-0.124273	-0.118353	-4.488487	-2.227495	-0.205564
H	0.149792	0.026025	-4.441446	-1.956954	-2.343949
H	0.157368	0.025834	-4.383340	-2.190438	1.945282
H	0.152752	0.030082	-4.939286	-3.214877	-0.253795
H	-0.144756	-0.421676	3.530308	-2.840138	-0.737180
B	0.885560	1.186447	3.709372	0.568331	-0.210114
C	-0.857538	-0.192795	3.465855	1.313625	1.238209
H	0.177485	-0.077134	3.419150	0.596391	2.075776
H	0.105026	-0.079985	4.282189	2.017170	1.469396
H	0.258374	-0.087976	2.530488	1.896453	1.272363
C	-0.744455	-0.185804	3.649201	1.642893	-1.447825
H	0.118633	-0.080113	4.396954	2.444855	-1.338054
H	0.128376	-0.080954	3.837889	1.152792	-2.416907
H	0.252003	-0.090359	2.664760	2.132007	-1.531990
C	-1.008969	-0.218417	5.159724	-0.210335	-0.207140

H	0.115888	-0.074714	5.345585	-0.752388	-1.148446
H	0.114956	-0.070578	5.985747	0.507933	-0.082942
H	0.118804	-0.080673	5.264898	-0.946656	0.609085
C	-0.783410	-0.456162	-0.163490	0.414417	1.378122
H	0.229606	0.017056	-0.701769	-0.539084	1.433370
H	0.257509	0.079428	0.906911	0.202444	1.273164
H	0.132880	-0.001913	-0.316359	0.954359	2.320916

XI°

Electronic energy: -680.018574

Sum of electronic and zero-point Energies= -679.787872

Sum of electronic and thermal Energies= -679.768156

Sum of electronic and thermal Enthalpies= -679.766974

Sum of electronic and thermal Free Energies= -679.842435

Table S56. Cartesian coordinates, Mulliken and APT charges of all atoms at **XI°** in the reaction of styrene with PMDSO.

Symbol	X	Y	Z	Mulliken	APT
Si	-1.824013	-0.329375	-0.469992	1.088412	1.496089
C	-3.625706	0.003376	-0.036190	-0.842422	-0.420083
H	-4.292417	-0.607990	-0.652202	0.184363	-0.005007
H	-3.820079	-0.240801	1.013649	0.180948	0.014222
H	-3.889908	1.052974	-0.195160	0.184617	0.017690
C	-1.434756	-2.161160	-0.314367	-0.871325	-0.413284
H	-2.090391	-2.756176	-0.957329	0.179840	0.008292
H	-0.397944	-2.367786	-0.595523	0.206915	0.024152
H	-1.578639	-2.497799	0.718169	0.177261	0.015904
C	-0.683389	0.684689	0.670097	-0.538950	-0.386170
H	-0.883289	0.337128	1.693364	0.138493	-0.032212
C	-1.017209	2.182569	0.588808	-0.534680	0.076866
H	-0.294269	2.778922	1.155864	0.183864	-0.031677
H	-1.013091	2.548514	-0.443533	0.177547	-0.002927
H	-2.012866	2.377882	0.997825	0.158988	-0.018021
C	0.753607	0.356890	0.344800	-0.806136	0.155743
C	1.442344	-0.622227	1.074113	0.221261	-0.115759
C	1.425402	0.977191	-0.717548	0.114606	-0.130244
C	2.753554	-0.970892	0.757303	-0.083291	0.010437

H	0.939199	-1.116222	1.902544	0.149269	0.033677
C	2.738666	0.633214	-1.036064	-0.145196	0.020375
H	0.918326	1.737494	-1.305270	0.128112	0.047598
C	3.409759	-0.342902	-0.301142	-0.096799	-0.105220
H	3.264620	-1.730469	1.341416	0.159821	0.031317
H	3.237791	1.130398	-1.862489	0.152655	0.030649
H	4.432453	-0.609441	-0.548238	0.144239	0.036940
H	-1.556945	0.130125	-1.862064	-0.012408	-0.359347

XI^H

Electronic energy: -1163.90524

Sum of electronic and zero-point Energies= -1163.566011

Sum of electronic and thermal Energies= -1163.533361

Sum of electronic and thermal Enthalpies= -1163.532180

Sum of electronic and thermal Free Energies= -1163.637631

Table S57. Cartesian coordinates, Mulliken and APT charges of all atoms at **XI^H** in the reaction of styrene with PMDSO.

Symbol	X	Y	Z	Mulliken	APT
Si	0.193846	-0.636775	0.128850	0.397254	2.037855
O	1.736246	-0.476221	-0.471755	-0.321520	-1.681738
Si	3.245301	0.090381	-0.053184	0.953695	2.070152
C	3.225538	1.966584	-0.019036	-0.783754	-0.460111
H	3.022903	2.382721	-1.010985	0.190402	0.017109
H	2.457489	2.339798	0.667432	0.187413	0.012743
H	4.193061	2.354273	0.318196	0.159194	0.011540
C	3.719099	-0.559304	1.643517	-0.713335	-0.463102
H	3.576806	-1.643510	1.703731	0.202285	0.014724
H	4.773050	-0.346360	1.852986	0.177305	0.015626
H	3.121576	-0.091938	2.432685	0.210499	0.019710
C	-0.299694	-2.438329	0.022969	-0.851216	-0.448334
H	0.406506	-3.054434	0.589206	0.170267	0.010132
H	-1.300828	-2.598715	0.435307	0.207331	0.024693
H	-0.296586	-2.787058	-1.014763	0.183295	0.018938
C	0.123901	-0.044183	1.909447	-0.520388	-0.464538
H	0.708515	-0.706212	2.556535	0.200082	0.018573
H	0.517122	0.972502	2.020918	0.252647	0.017786

H	-0.909666	-0.048453	2.271433	0.201543	0.033011
C	-0.928759	0.441078	-0.958407	-0.932116	-0.431957
H	-0.856547	0.014816	-1.968674	0.085091	-0.025019
C	-0.402835	1.882855	-1.003238	-0.228158	0.067160
H	-1.080007	2.535342	-1.565300	0.191454	-0.036146
H	-0.284154	2.312889	-0.002255	0.231456	0.000095
H	0.579309	1.909770	-1.482793	0.154295	-0.006895
C	-2.362509	0.311684	-0.504495	-0.261138	0.169945
C	-3.170448	-0.719351	-1.006416	0.624470	-0.118620
C	-2.920600	1.173438	0.449315	-0.526462	-0.131812
C	-4.481975	-0.889320	-0.570310	-0.133308	0.013334
H	-2.759576	-1.396367	-1.752502	0.166097	0.037029
C	-4.234271	1.007519	0.888484	-0.162798	0.016969
H	-2.325380	1.986287	0.856107	0.096471	0.048299
C	-5.021695	-0.025053	0.382797	-0.000538	-0.103229
H	-5.085141	-1.694398	-0.979416	0.150802	0.031462
H	-4.642593	1.690766	1.627313	0.157531	0.030625
H	-6.044537	-0.152449	0.722720	0.152761	0.035829
C	4.441228	-0.530951	-1.351004	-0.822456	-0.442271
H	5.455816	-0.170110	-1.152201	0.175852	0.009290
H	4.466246	-1.625019	-1.366052	0.191090	0.014398
H	4.146713	-0.184863	-2.346640	0.186606	0.016745

XIq

Electronic energy: -719.330744

Sum of electronic and zero-point Energies= -719.071466

Sum of electronic and thermal Energies= -719.049077

Sum of electronic and thermal Enthalpies= -719.047895

Sum of electronic and thermal Free Energies= -719.128991

Table S58. Cartesian coordinates, Mulliken and APT charges of all atoms at **XIq** in the reaction of styrene with PMDSO.

Symbol	X	Y	Z	Mulliken	APT
Si	-1.724040	0.337180	0.202255	1.153888	1.613213
C	-3.479092	-0.056683	-0.361444	-0.853958	-0.435235
H	-4.207204	0.529056	0.209421	0.177208	-0.010152
H	-3.615750	0.179600	-1.422188	0.181430	0.010653

H	-3.715557	-1.115815	-0.217247	0.175564	0.013184
C	-1.421958	2.191638	0.098841	-0.822647	-0.432392
H	-2.142439	2.732209	0.721709	0.176936	0.005106
H	-0.415455	2.445818	0.446090	0.219455	0.020260
H	-1.527846	2.554368	-0.929294	0.183889	0.011269
C	-0.497431	-0.574076	-0.946156	-0.749068	-0.412911
H	-0.630630	-0.123148	-1.939684	0.109556	-0.034319
C	-0.838551	-2.068894	-1.044037	-0.448533	0.075945
H	-0.075230	-2.612251	-1.611952	0.186014	-0.033839
H	-0.915921	-2.542801	-0.059336	0.183145	-0.002985
H	-1.799718	-2.210478	-1.546478	0.158080	-0.015833
C	0.917010	-0.293237	-0.502263	-0.535621	0.164116
C	1.607124	0.821826	-0.999755	0.478017	-0.117937
C	1.569968	-1.097645	0.441865	-0.205090	-0.130455
C	2.896431	1.126882	-0.569991	-0.187577	0.015256
H	1.121372	1.457523	-1.737144	0.160178	0.034227
C	2.861296	-0.796497	0.874408	-0.223572	0.017969
H	1.065728	-1.970448	0.847543	0.129491	0.045727
C	3.531544	0.317522	0.372150	0.020897	-0.106350
H	3.407701	1.994788	-0.975845	0.149493	0.030981
H	3.344590	-1.437512	1.605840	0.151185	0.030059
H	4.537417	0.550204	0.706959	0.152849	0.036069
C	-1.479179	-0.247321	1.975038	-0.728364	-0.433507
H	-0.476973	0.003947	2.336509	0.223358	0.018672
H	-2.208516	0.233932	2.635263	0.185967	0.009464
H	-1.611987	-1.330607	2.066331	0.197828	0.013745

Hexamethyldisiloxane (HMDSO)

V

Electronic energy: -1348.423865

Electronic energy + zero-point energy: -1347.933714

Electronic energy + thermal energy correction: -1347.889266

Electronic energy + thermal enthalpy correction: -1347.888084

Electronic energy + thermal free energy correction: -1347.019444

Table S59. Cartesian coordinates, Mulliken and APT charges of all atoms at **V** in the reaction of styrene with HMDSO.

Symbol	X	Y	Z	Mulliken	APT
H	0.182264	0.008658	6.249952	-1.742442	-0.516020
H	0.165966	-0.089034	-1.792296	-0.942057	-2.214971
H	0.167857	0.007532	1.170822	-2.358822	-2.175662
H	0.167344	0.001417	6.913634	-0.555288	0.623058
C	-0.651579	-0.442772	4.839847	1.100609	-1.332313
C	-0.828488	-0.430349	6.027659	-1.154314	0.381250
C	-0.563732	0.106790	-2.709964	-1.250221	-1.686403
H	0.142691	-0.027219	-3.563897	-0.910502	-2.287265
H	0.120624	-0.032019	-2.746861	-2.345254	-1.685300
H	0.164350	0.037742	-3.017935	1.305956	-2.099019
C	-1.010031	-0.418229	0.874942	-2.368152	-1.120631
Si	0.843341	2.008873	4.520861	-0.066756	0.107693
H	0.136781	-0.074694	-4.932324	-2.787728	-0.869241
H	0.186942	0.010347	5.855016	-1.855190	1.205427
H	0.133892	-0.076668	-5.495416	-0.086802	-1.390418
O	-0.364280	-1.598410	3.244119	-1.059669	-0.227011
H	0.210806	0.034678	-0.220144	-2.367334	-1.073982
H	0.158151	0.004482	1.236970	-3.301038	-0.673506
C	-0.060455	-0.161972	-2.528855	1.630508	-1.183812
H	0.134104	0.007849	-2.245730	3.635961	-1.907183
Si	1.430733	1.967436	1.567472	-0.891785	-0.206127
C	-0.843510	-0.162279	-4.749795	-2.533356	0.187813
C	-0.357919	-0.414393	-2.787773	-0.719518	-0.249165
C	-0.202883	0.056710	-2.092934	2.950942	-1.075412
C	-0.725849	-0.179163	-5.433397	0.035073	-0.295062
H	0.145983	-0.087835	-3.981698	-3.235429	0.558401
B	1.017663	0.796321	-4.333677	-0.955480	0.423840
C	-1.681666	0.285520	-2.346828	0.703328	-0.142345
C	-1.048590	-0.449531	1.150328	0.723912	-1.057225
H	0.125658	-0.091254	-6.444180	-0.173833	0.093443
H	0.124450	-0.118317	-5.680185	-2.771657	0.729570
H	0.162985	0.008967	5.046067	1.561761	1.922957
H	0.166144	-0.067434	-5.237515	1.102808	-0.105012
C	-0.708426	-0.447938	4.176862	0.948391	1.655595
H	0.112284	-0.082289	-2.108070	-1.322488	0.379235
C	-0.001974	-0.175860	-1.460116	3.397052	0.084032
C	-0.932881	-0.442520	1.019765	-0.928115	1.590535
H	0.191739	0.010356	3.325999	1.624498	1.507756
H	0.167125	-0.003348	1.592340	-1.684601	2.141010
C	0.828193	-0.167280	-1.734981	1.186987	1.029216
H	0.196807	0.012700	3.942698	0.301886	2.509049
H	0.143509	0.017806	-1.109575	4.423039	0.164899
C	-0.813564	-0.168621	-4.287355	-0.646323	2.039959
H	0.137982	0.034305	-0.042581	-1.184081	1.665645

C	-0.214781	0.053472	-1.293244	2.501359	1.142835
H	0.118737	-0.093267	-5.258059	-0.885653	2.505156
H	0.222735	0.022310	1.162415	0.041274	2.081544
H	0.185372	0.038411	-1.606779	0.502304	1.864367
H	0.137864	-0.084067	-3.529327	-1.251205	2.570257
H	0.165345	-0.066907	-4.079232	0.409063	2.278388
H	0.129294	0.014830	-0.813284	2.829404	2.063293
H	0.182429	-0.003115	1.617669	0.765585	-2.049229
H	0.314435	0.014354	1.498754	1.587079	-0.475586
H	0.143642	0.065126	0.067765	0.844620	-1.179581
H	0.169232	0.016406	3.999925	1.789711	-1.474018
H	0.193733	0.008231	4.975982	0.542642	-2.265675
H	0.181420	0.005154	5.742893	1.699157	-1.160031

VI

Electronic energy: -1348.364951

Electronic energy + zero-point energy: -1347.877581

Electronic energy + thermal energy correction: -1347.834856

Electronic energy + thermal enthalpy correction: -1347.833674

Electronic energy + thermal free energy correction: -1347.957130

Table S60. Cartesian coordinates, Mulliken and APT charges of all atoms at **VI** in the reaction of styrene with HMDSO.

Symbol	X	Y	Z	Mulliken	APT
H	0.176400	-0.001613	5.665729	-1.860705	0.858110
H	0.092727	-0.073981	-1.106622	-0.847473	-2.623698
H	0.140872	0.004097	0.665829	-2.526277	-1.994468
H	0.166188	-0.018549	6.194593	-0.351934	1.632495
C	-0.755678	-0.438891	5.168771	0.508318	-1.337346
C	-0.750550	-0.435322	5.315897	-0.942053	1.343526
C	-0.653241	0.263442	-1.789903	-1.177974	-1.822310
H	0.119351	-0.106891	-2.808003	-0.922972	-2.172141
H	0.159525	-0.024597	-1.727846	-2.269980	-1.795332
H	0.156297	0.029418	-2.175057	1.287668	-2.326239
C	-0.920825	-0.451297	0.849587	-2.517082	-0.914052
Si	0.470617	2.029779	4.169856	0.025340	0.194308
H	0.142788	-0.007332	-4.184601	-2.813205	-1.274930

H	0.179988	0.001725	4.783698	-1.231317	2.257469
H	0.187630	0.023003	-4.648877	-0.145631	-1.109369
O	-0.548550	-1.690115	2.896216	-0.903231	-0.209293
H	0.126679	-0.005797	-0.004928	-2.999017	-0.427897
H	0.152028	-0.047640	1.755351	-3.100335	-0.717682
C	0.129328	-0.372796	-2.167090	1.669195	-1.308082
H	0.130313	-0.005100	-2.750169	3.627753	-1.948357
Si	1.925557	2.439275	1.131581	-0.753040	-0.278446
C	-0.790110	-0.303200	-4.039098	-2.894036	-0.192276
C	-0.970372	-1.253410	-1.445489	-0.577232	-0.476157
C	-0.272858	0.238235	-2.493658	3.003758	-1.093108
C	-0.772276	-0.317488	-4.869366	-0.284055	-0.043582
H	0.189456	0.001693	-3.109127	-3.445645	-0.005668
B	0.925800	0.989399	-4.091785	-1.508679	0.580739
C	-1.120658	0.715872	-1.820542	0.789746	-0.243356
C	-0.724482	-0.434350	1.086711	0.757952	-1.427276
H	0.139842	-0.020534	-5.949556	-0.500029	0.026420
H	0.150439	-0.027165	-4.854521	-3.523367	0.202561
H	0.152943	-0.008454	4.540320	2.249073	1.272713
H	0.204935	0.009843	-4.682556	0.669095	0.464026
C	-0.675270	-0.448196	3.664148	1.616701	1.078376
H	0.455360	0.034583	-1.560312	-1.245348	0.380422
C	-0.233014	-0.425231	-2.503818	3.551235	0.191314
C	-0.880152	-0.442376	0.858746	-0.504708	1.588348
H	0.221166	0.012065	2.956452	2.196705	0.474279
H	0.163474	-0.039652	1.742430	-0.857171	2.134444
C	0.537720	-0.339589	-1.843256	1.378192	1.054223
H	0.204372	0.011182	3.181568	1.403737	2.039123
H	0.126557	0.011995	-2.762084	4.593460	0.357454
C	-0.758205	-0.308221	-3.701655	-1.489429	2.116773
H	0.098560	-0.011485	-0.019509	-1.039468	1.963799
C	-0.309118	0.242791	-2.173225	2.708858	1.261218
H	0.151098	-0.018869	-4.617686	-1.713840	2.689450
H	0.268651	0.016354	0.718145	0.558662	1.816255
H	0.141181	0.025879	-1.594605	0.756689	1.910674
H	0.160476	-0.012778	-2.955749	-2.241205	2.402537
H	0.207024	0.023131	-3.363354	-0.503607	2.452547
H	0.123898	-0.000702	-2.176253	3.099896	2.277905
H	0.135034	-0.041351	2.071450	0.941376	-1.874017
H	0.230350	0.014601	0.791376	1.642989	-0.849318
H	0.172211	0.006666	0.350982	0.664581	-2.231849
H	0.180580	0.008874	4.576933	1.141957	-2.008038
H	0.175941	-0.004398	5.468863	-0.383456	-1.899748
H	0.162003	-0.016534	6.076218	1.061069	-1.062751

VII

Electronic energy: -1348.367282

Electronic energy + zero-point energy: -1347.878863

Electronic energy + thermal energy correction: -1347.834443

Electronic energy + thermal enthalpy correction: -1347.833262

Electronic energy + thermal free energy correction: -1347.961175

Table S61. Cartesian coordinates, Mulliken and APT charges of all atoms at **VII** in the reaction of styrene with HMDSO.

Symbol	X	Y	Z	Mulliken	APT
H	0.169822	-0.009374	5.763176	-1.841075	0.411967
H	0.134334	-0.033865	-0.833990	-0.699072	-2.674771
H	0.165774	-0.015786	1.078789	-2.704292	-1.733140
H	0.158103	-0.026641	6.391765	-0.423271	1.279521
C	-0.759610	-0.442700	4.957389	0.787729	-1.355629
C	-0.825746	-0.432113	5.483489	-0.996682	1.053139
C	-0.613121	0.143149	-1.486680	-1.131431	-1.901460
H	0.125518	-0.090648	-2.525365	-0.939765	-2.221635
H	0.142785	-0.020962	-1.330281	-2.213696	-1.930901
H	0.126389	0.036654	-1.954562	1.327410	-2.343635
C	-0.732394	-0.416580	0.950569	-2.551295	-0.652975
Si	0.648174	2.026010	4.163783	0.072839	0.212500
H	0.184519	0.016230	-3.619168	-2.895356	-1.192940
H	0.175001	-0.002282	5.097105	-1.408795	1.993160
H	0.167781	-0.000687	-4.759264	-0.480296	-1.395548
O	-0.464421	-1.690090	2.852043	-0.811739	-0.102524
H	0.144922	-0.034455	0.014220	-3.037366	-0.354443
H	0.163617	-0.038628	1.791608	-3.040934	-0.153137
C	-0.148656	-0.239519	-2.093738	1.645622	-1.313670
H	0.135941	0.004002	-2.897591	3.543205	-1.915505
Si	1.420988	2.391469	1.002168	-0.684535	-0.232245
C	-0.777971	-0.308308	-3.849314	-2.935209	-0.123233
C	-1.518229	-0.921426	-1.167254	-0.573143	-0.514858
C	-0.315501	0.109165	-2.637797	2.906078	-1.072089
C	-0.774929	-0.300417	-4.887753	-0.402829	-0.310252
H	0.174681	-0.002789	-3.059027	-3.499180	0.386090
B	0.854358	0.907920	-4.131879	-1.514108	0.515296
C	0.507504	0.455956	-1.740726	0.765770	-0.267399

C	-0.687354	-0.400269	1.262283	0.689683	-1.549317
H	0.142580	-0.007485	-5.964171	-0.535248	-0.101130
H	0.147486	-0.010267	-4.776658	-3.524614	-0.022196
H	0.147444	-0.018823	4.735784	2.146459	1.504412
H	0.237988	0.026085	-4.630241	0.615716	0.002827
C	-0.691821	-0.441854	3.829647	1.541200	1.367344
H	0.530724	-0.036279	-1.503649	-1.269505	0.268328
C	-0.193848	-0.247146	-2.862372	3.349789	0.229709
C	-0.858062	-0.423318	0.845837	-0.300594	1.638660
H	0.181008	-0.003015	3.046163	2.193694	0.963359
H	0.197351	-0.035647	1.739929	-0.654205	2.162663
C	0.014468	-0.228260	-1.972108	1.247407	1.041457
H	0.191363	-0.000554	3.495257	1.201297	2.354483
H	0.142754	0.015282	-3.294233	4.329181	0.418423
C	-0.785541	-0.300792	-3.895763	-1.331803	2.071274
H	0.065577	-0.030801	-0.030442	-0.788581	2.082283
C	-0.628078	0.116173	-2.520180	2.500058	1.286303
H	0.145145	-0.011411	-4.534816	-2.049074	2.610918
H	0.234952	0.004683	0.747893	0.777621	1.822075
H	0.184215	0.041516	-1.730765	0.606015	1.884284
H	0.202269	0.018456	-2.864463	-1.589550	2.346895
H	0.173959	-0.009555	-4.111909	-0.326907	2.448418
H	0.136707	0.010225	-2.688713	2.815785	2.314359
H	0.196593	-0.025844	1.956846	0.319031	-2.313403
H	0.244857	-0.022486	1.757542	1.540319	-1.060018
H	0.140255	-0.013459	0.362366	1.073644	-2.036096
H	0.184741	0.005319	4.293732	1.517645	-1.834030
H	0.175480	-0.006764	5.155327	-0.008297	-2.083768
H	0.157154	-0.026997	5.908363	1.288150	-1.130038

VIII

Electronic energy: -1348.366356

Electronic energy + zero-point energy: -1347.878068

Electronic energy + thermal energy correction: -1347.833576

Electronic energy + thermal enthalpy correction: -1347.832394

Electronic energy + thermal free energy correction: -1347.960669

Table S62. Cartesian coordinates, Mulliken and APT charges of all atoms at **VIII** in the reaction of styrene with HMDSO.

Symbol	X	Y	Z	Mulliken	APT
H	0.174778	-0.008517	3.504350	-2.121749	-2.044181
H	0.216249	-0.018712	-1.589117	2.693604	0.335547
H	0.307975	-0.013383	1.185909	1.884888	-1.594542
H	0.167217	-0.027988	3.470032	-3.690477	-1.213570
C	-0.950511	-0.434406	4.032976	-1.532540	1.075666
C	-0.641393	-0.435801	2.887609	-2.783341	-1.422262
C	-0.085942	0.147914	-1.916175	2.487373	-0.692846
H	0.169301	-0.104360	-2.851849	3.049614	-0.848869
H	0.139460	-0.029867	-1.155543	2.919846	-1.352639
H	0.094094	0.033164	-3.362136	1.764576	1.371944
C	-0.315243	-0.403060	0.607687	1.029974	-1.965061
Si	1.005727	2.012160	2.397546	-1.885885	0.179021
H	0.124357	-0.016146	4.998316	1.494177	-0.514386
H	0.164967	-0.001237	2.012333	-3.070685	-2.016039
H	0.151825	-0.013491	4.256473	1.499070	2.085387
O	-0.520241	-1.677932	1.568426	-0.529264	-0.111529
H	0.162505	-0.038079	-0.107321	1.403614	-2.706633
H	0.176548	-0.044471	1.321305	0.359873	-2.458907
C	-0.827016	-0.228467	-3.814463	0.895671	0.900791
H	0.137136	0.004871	-5.299835	0.718095	2.443078
Si	-0.817053	2.303039	-0.202078	0.056159	-0.519011
C	-0.888280	-0.311206	3.987222	1.800614	-0.810955
C	-0.859118	-0.817704	-2.072859	0.986101	-0.954625
C	-0.083047	0.101777	-4.922367	0.305700	1.509213
C	-0.847009	-0.310839	3.245829	1.823168	1.807362
H	0.427571	0.078062	3.438603	0.875709	-1.058950
B	0.786050	0.857179	3.151593	2.441688	0.360713
C	-0.436950	0.461970	-3.272555	0.404225	-0.305275
C	-0.236387	-0.372335	-0.307000	0.567371	1.328902
H	0.111774	-0.027970	2.831739	2.452589	2.604177
H	0.125584	-0.027133	4.041310	2.405443	-1.723207
H	0.153717	-0.023510	2.104933	-3.994559	1.504485
H	0.342491	0.071936	2.632929	0.908183	1.756485
C	-0.739342	-0.440332	1.475725	-3.121470	1.285234
H	0.038953	-0.063937	-2.167967	0.819636	-2.038460
C	-0.199382	-0.239760	-5.550468	-0.796177	0.932991
C	-0.875528	-0.404221	-0.825685	-1.673948	-1.040306
H	0.165537	-0.001707	1.213955	-2.648993	2.240335
H	0.193155	-0.042698	-0.019472	-2.399339	-1.182947
C	0.854757	-0.207502	-3.946550	-0.697542	-0.877167

H	0.194835	0.007739	0.546140	-3.477040	0.827668
H	0.126511	0.014264	-6.414190	-1.255849	1.406373
C	-0.801812	-0.288769	2.173066	3.662664	0.119608
H	0.156275	-0.022431	-1.372227	-1.596466	-1.988566
C	-0.006941	0.103387	-5.051287	-1.288012	-0.276622
H	0.140109	-0.021054	2.105717	4.009448	-0.917154
H	0.216582	-0.006510	-1.526955	-2.080582	-0.300236
H	0.131147	0.025684	-3.583790	-1.091024	-1.824187
H	0.243002	0.017445	1.158169	3.417233	0.463258
H	0.146578	-0.011937	2.499500	4.507708	0.745731
H	0.140862	0.007015	-5.532813	-2.136779	-0.758376
H	0.329615	-0.019948	0.333802	1.439211	1.515962
H	0.208104	-0.028170	0.095136	-0.247619	1.941354
H	0.179034	-0.030766	-1.316236	0.803762	1.678907
H	0.183633	-0.001137	3.864976	-1.189674	2.103515
H	0.222877	-0.000159	4.613064	-0.758907	0.558759
H	0.120305	-0.029951	4.644597	-2.443693	1.119532

IX

Electronic energy: -1348.361027

Electronic energy + zero-point energy: -1347.869351

Electronic energy + thermal energy correction: -1347.825246

Electronic energy + thermal enthalpy correction: -1347.824064

Electronic energy + thermal free energy correction: -1347.949767

Table S63. Cartesian coordinates, Mulliken and APT charges of all atoms at **IX** in the reaction of styrene with HMDSO.

Symbol	X	Y	Z	Mulliken	APT
H	0.170386	-0.011356	-3.305775	-1.829622	2.187492
H	0.238517	-0.014334	1.680154	2.682829	-0.178917
H	0.255954	-0.005196	-1.282298	1.539330	1.947783
H	0.164550	-0.027668	-3.113890	-3.401089	1.391834
C	-1.061617	-0.435842	-4.392320	-1.331085	-0.651018
C	-0.592460	-0.431257	-2.662361	-2.406950	1.509709
C	0.087190	0.130009	1.993974	2.393854	0.834011
H	0.177386	-0.096869	2.942753	2.914045	1.041671
H	0.115059	-0.024792	1.238120	2.794795	1.519623

H	0.127954	0.037797	3.491652	1.781894	-1.238657
C	-0.089242	-0.388673	-0.528663	0.763936	2.096481
Si	1.238124	1.969289	-2.562923	-1.503618	-0.162367
H	0.120379	-0.043612	-4.837578	1.891276	0.719230
H	0.178720	0.008770	-1.692659	-2.527196	2.001906
H	0.157167	-0.030104	-4.414254	1.075819	-1.815707
O	-0.472177	-1.739343	-1.710163	-0.117615	-0.059205
H	0.154972	-0.045862	0.212458	1.121596	2.820621
H	0.211284	-0.044072	-1.059120	-0.089881	2.540164
C	-0.633692	-0.210578	3.902916	0.866424	-0.820794
H	0.137551	0.006929	5.441895	0.757239	-2.316909
Si	-1.802129	2.203165	0.236319	0.098095	0.455070
C	-0.776070	-0.266244	-3.770858	1.766275	0.958523
C	-0.856779	-0.750327	2.103009	0.872751	0.986442
C	-0.116893	0.083883	5.015582	0.288303	-1.432278
C	-0.768420	-0.263531	-3.448567	1.589522	-1.800105
H	0.274087	0.016951	-3.658115	0.777039	1.414803
B	0.747723	1.077069	-2.862196	1.908551	-0.352547
C	-0.588261	0.411279	3.304195	0.301146	0.321653
C	0.040832	-0.371776	0.418915	0.582726	-1.389118
H	0.118164	-0.040758	-3.576645	2.544640	-2.334471
H	0.122844	-0.044458	-3.514617	2.501421	1.733638
H	0.160087	-0.025803	-2.458850	-3.607154	-1.528900
H	0.232478	0.009937	-2.731009	1.001386	-2.387177
C	-0.635799	-0.426660	-1.892289	-2.666517	-1.507008
H	0.053416	-0.055455	2.180832	0.627744	2.055815
C	-0.177265	-0.213571	5.586484	-0.874250	-0.919059
C	-0.664575	-0.414875	0.722815	-1.731195	0.702392
H	0.161910	-0.010468	-2.021806	-2.179030	-2.482479
H	0.199990	-0.014706	-0.072597	-2.467361	0.576123
C	0.742643	-0.195588	3.914831	-0.864786	0.829325
H	0.186344	0.005131	-0.830429	-2.906905	-1.399887
H	0.131137	0.015626	6.454425	-1.323860	-1.394340
C	-0.694192	-0.284965	-1.731167	3.034970	-0.330835
H	0.174365	-0.024841	1.124630	-1.856540	1.717208
C	0.024306	0.085215	5.024997	-1.443772	0.226701
H	0.217822	0.014978	-0.979599	2.943159	0.458736
H	0.197651	-0.013095	1.528018	-1.987626	0.002217
H	0.139093	0.027781	3.496495	-1.319468	1.725197
H	0.121660	-0.027051	-1.210589	3.180891	-1.283788
H	0.101059	-0.044283	-2.277346	3.971940	-0.123985
H	0.142487	0.010398	5.459209	-2.344856	0.655028
H	0.257122	0.004164	-0.209557	1.429252	-1.671315
H	0.220145	-0.022598	0.059349	-0.278149	-1.966954
H	0.174891	-0.030595	1.452747	0.789244	-1.684264

H	0.165667	-0.004282	-4.514133	-1.119551	-1.718236
H	0.171104	0.006198	-4.930342	-0.562534	-0.086255
H	0.115350	-0.029083	-4.875742	-2.297337	-0.448438

X

Electronic energy: -1348.415266

Electronic energy + zero-point energy: -1347.924980

Electronic energy + thermal energy correction: -1347.880568

Electronic energy + thermal enthalpy correction: -1347.879387

Electronic energy + thermal free energy correction: -1348.007546

Table S64. Cartesian coordinates, Mulliken and APT charges of all atoms at **X** in the reaction of styrene with HMDSO.

Symbol	X	Y	Z	Mulliken	APT
H	0.217183	0.000634	-3.412091	-1.093386	2.364382
H	0.169599	0.004499	2.369039	2.812473	-0.334007
H	0.172968	0.020989	-0.184551	2.111299	1.872243
H	0.156943	-0.024355	-3.045429	-2.797985	2.014999
C	-0.942886	-0.443064	-4.838456	-1.914848	-0.449206
C	-0.468481	-0.422713	-2.781499	-1.761444	1.764932
C	-0.239547	0.074861	2.681976	2.592514	0.692303
H	0.179571	-0.050483	3.668882	3.048854	0.843566
H	0.150215	-0.012722	1.969087	3.094424	1.354279
H	0.153013	0.049193	3.516128	1.389960	-1.637386
C	-0.744915	-0.457769	-0.246996	1.018255	1.832330
Si	0.122759	1.966179	-3.047834	-1.417062	-0.083532
H	0.113514	-0.078564	-4.650813	2.575129	1.095194
H	0.273823	-0.012746	-1.742494	-1.593911	2.072462
H	0.148158	-0.076205	-5.409175	0.690903	-1.398461
O	-0.566797	-1.512722	-2.650710	0.095695	-0.498415
H	0.142823	-0.012828	-0.100203	0.625977	2.846894
H	0.186269	0.063876	-1.260419	0.768222	1.496595
C	0.000117	-0.140555	4.073531	0.615024	-1.117203
H	0.151573	0.025037	5.237773	0.119104	-2.854145
Si	0.651191	1.603396	0.995340	0.269343	0.635844
C	-0.925163	-0.205180	-4.096507	1.624741	1.035349
C	-0.985365	-0.439413	2.712754	1.084093	0.977675

C	-0.123846	0.030874	5.045528	-0.106681	-1.808225
C	-0.941570	-0.213257	-4.594380	1.401163	-1.611906
H	0.151410	-0.085611	-4.820558	0.828690	1.284127
B	1.391158	1.199921	-3.424362	1.422389	-0.455989
C	-0.514433	0.201575	3.793560	0.350422	0.231757
C	-0.654414	-0.482958	0.521371	0.529646	-1.159879
H	0.124360	-0.075703	-5.064397	2.391645	-1.725670
H	0.168290	-0.074326	-3.351508	1.641790	1.848264
H	0.163953	-0.021478	-2.005356	-3.632613	-0.697085
H	0.134428	-0.072532	-4.177429	1.128976	-2.595575
C	-0.579384	-0.401474	-1.947564	-2.602645	-1.072407
H	0.081037	-0.044448	2.893363	0.941277	2.053904
C	-0.118112	-0.121239	5.765272	-1.115047	-1.169292
C	-0.795826	-0.415647	1.198494	-1.572171	1.014156
H	0.170038	-0.013327	-2.259148	-2.606169	-2.124380
H	0.179982	0.013038	0.240166	-2.098590	0.951045
C	0.416792	-0.128904	4.528645	-0.667221	0.857553
H	0.267148	-0.011883	-0.897716	-2.287741	-1.046870
H	0.145564	0.027941	6.522545	-1.677382	-1.708643
C	-0.903946	-0.192722	-2.346464	2.615257	-0.787880
H	0.169747	-0.005164	1.592857	-1.718890	2.028255
C	-0.084718	0.023295	5.499314	-1.392383	0.171308
H	0.216937	-0.085256	-1.501370	2.641504	-0.081077
H	0.197797	0.003446	1.893992	-2.045938	0.311026
H	0.158313	0.033061	4.325004	-0.895118	1.902332
H	0.111193	-0.070694	-1.914945	2.502590	-1.796000
H	0.084275	-0.083231	-2.824287	3.607479	-0.751714
H	0.153247	0.022351	6.049150	-2.176749	0.685639
H	0.253691	0.017264	0.633991	1.576253	-1.466547
H	0.235053	0.121610	-0.542732	0.282407	-1.278803
H	0.165505	0.000609	1.124319	-0.097960	-1.827550
H	0.159781	-0.003910	-5.078729	-1.761832	-1.507405
H	0.169969	0.010856	-5.545349	-1.321074	0.141509
H	0.130017	-0.021422	-4.996670	-2.975110	-0.208683

XI

Electronic energy: -719.330744

Sum of electronic and zero-point Energies= -719.071466

Sum of electronic and thermal Energies= -719.049077

Sum of electronic and thermal Enthalpies= -719.047895

Sum of electronic and thermal Free Energies= -719.128991

Table S65. Cartesian coordinates, Mulliken and APT charges of all atoms at **XI** in the reaction of styrene with HMDSO.

Symbol	X	Y	Z	Mulliken	APT
Si	-1.724040	0.337180	0.202255	1.153888	1.613213
C	-3.479092	-0.056683	-0.361444	-0.853958	-0.435235
H	-4.207204	0.529056	0.209421	0.177208	-0.010152
H	-3.615750	0.179600	-1.422188	0.181430	0.010653
H	-3.715557	-1.115815	-0.217247	0.175564	0.013184
C	-1.421958	2.191638	0.098841	-0.822647	-0.432392
H	-2.142439	2.732209	0.721709	0.176936	0.005106
H	-0.415455	2.445818	0.446090	0.219455	0.020260
H	-1.527846	2.554368	-0.929294	0.183889	0.011269
C	-0.497431	-0.574076	-0.946156	-0.749068	-0.412911
H	-0.630630	-0.123148	-1.939684	0.109556	-0.034319
C	-0.838551	-2.068894	-1.044037	-0.448533	0.075945
H	-0.075230	-2.612251	-1.611952	0.186014	-0.033839
H	-0.915921	-2.542801	-0.059336	0.183145	-0.002985
H	-1.799718	-2.210478	-1.546478	0.158080	-0.015833
C	0.917010	-0.293237	-0.502263	-0.535621	0.164116
C	1.607124	0.821826	-0.999755	0.478017	-0.117937
C	1.569968	-1.097645	0.441865	-0.205090	-0.130455
C	2.896431	1.126882	-0.569991	-0.187577	0.015256
H	1.121372	1.457523	-1.737144	0.160178	0.034227
C	2.861296	-0.796497	0.874408	-0.223572	0.017969
H	1.065728	-1.970448	0.847543	0.129491	0.045727
C	3.531544	0.317522	0.372150	0.020897	-0.106350
H	3.407701	1.994788	-0.975845	0.149493	0.030981
H	3.344590	-1.437512	1.605840	0.151185	0.030059
H	4.537417	0.550204	0.706959	0.152849	0.036069
C	-1.479179	-0.247321	1.975038	-0.728364	-0.433507
H	-0.476973	0.003947	2.336509	0.223358	0.018672
H	-2.208516	0.233932	2.635263	0.185967	0.009464
H	-1.611987	-1.330607	2.066331	0.197828	0.013745

Regeneration of $NaHBMe_3$

As it was not possible to compute energy profiles for the regeneration of $HBMe_3^-$ using the same protocol as for other reactions (omitting cations would result in a system with double negative

charge, for which there would be no minimum on the potential energy hypersurface), free energy of each step in this reaction was using a different protocol.

$$\Delta G_{substrates} = \left(\Delta G_{BMe_3OSiMe_2HNa} - \Delta G_{BMe_3} - \Delta G_{OSiMe_2HNa} + E_{BSSE}\right) + \Delta G_{NaH}$$

$$\Delta G_{TS} = \left(\Delta G_{NaHBMe_3OSiMe_2HNa} - \Delta G_{NaH} - \Delta G_{BMe_3} - \Delta G_{OSiMe_2HNa} + E_{BSSE}\right)$$

$$\Delta G_{products} = \left(\Delta G_{NaHBMe_3} - \Delta G_{NaH} - \Delta G_{BMe_3} + E_{BSSE}\right) + \Delta G_{OSiMe_2HNa}$$

Where ΔG_{OSiMe_2HNa}, ΔG_{NaH} and ΔG_{BMe_3} are Gibbs free energies calculated for individual reagents and E_{BSSE} is basis set superposition error as described by Boys and Bernardi (S. F. Boys and F. Bernardi, Mol. Phys., 19 (1970) 553). All other computation parameters, including functional, basis set, solvation model and temperature were the same as in the rest of the study.

BMe_3

Electronic energy: -144.531111

Electronic energy + zero-point energy: -144.418178

Electronic energy + thermal energy correction: -144.410190

Electronic energy + thermal enthalpy correction: -144.409008

Electronic energy + thermal free energy correction: -144.455016

Table S66. Cartesian coordinates, Mulliken and APT charges of all atoms at **BMe_3**.

Symbol	X	Y	Z	Mulliken	APT
B	2.632584	0.546426	-0.005786	0.616620	0.884320
C	1.817826	1.895195	0.000989	-0.731687	-0.309523
H	2.271966	2.647933	0.655852	0.175063	0.007416
H	1.879118	2.316962	-1.013905	0.184349	0.019375
H	0.757574	1.792558	0.250031	0.166183	-0.014664
C	1.864448	-0.829086	0.043705	-0.732082	-0.308145
H	2.489744	-1.716983	-0.085667	0.164497	-0.018949
H	1.357583	-0.908633	1.016117	0.182478	0.018583

H	1.058802	-0.851480	-0.701129	0.178027	0.012771
C	4.207256	0.574335	-0.051982	-0.728865	-0.307131
H	4.580115	0.187157	0.907999	0.182723	0.021786
H	4.598364	-0.113669	-0.811134	0.176589	0.009154
H	4.647411	1.563937	-0.205091	0.166105	-0.014991

NaH

Electronic energy: -162.828589

Electronic energy + zero-point energy: -162.826226

Electronic energy + thermal energy correction: -162.823184

Electronic energy + thermal enthalpy correction: -162.822002

Electronic energy + thermal free energy correction: -162.849838

Table S67. Cartesian coordinates, Mulliken and APT charges of all atoms at **NaH**.

Symbol	X	Y	Z	Mulliken	APT
Na	-0.701776	3.627907	0.000000	0.731737	0.815975
H	-2.672643	3.627907	0.000000	-0.731737	-0.815975

$OSiMe_2HNa$

Electronic energy: -607.412623

Electronic energy + zero-point energy: -607.326697

Electronic energy + thermal energy correction: -607.314618

Electronic energy + thermal enthalpy correction: -607.313436

Electronic energy + thermal free energy correction: -607.373288

Table S68. Cartesian coordinates, Mulliken and APT charges of all atoms at **$OSiMe_2HNa$**.

Symbol	X	Y	Z	Mulliken	APT
C	-1.530595	1.531923	-0.313328	-0.764778	-0.418950
Si	-0.674017	-0.002026	0.400175	0.573181	1.911543
H	-1.099962	2.451200	0.098132	0.183670	-0.015250
H	-2.603908	1.532793	-0.090601	0.181796	-0.014666

H	-1.412131	1.565479	-1.402836	0.173073	-0.014148
H	-1.014093	-0.000354	1.869203	-0.107403	-0.470418
O	0.907628	-0.026832	0.131940	-0.965590	-1.459976
C	-1.581070	-1.502265	-0.321965	-0.763169	-0.419763
H	-2.652830	-1.471201	-0.093992	0.181614	-0.015123
H	-1.177467	-2.437875	0.079884	0.184135	-0.014650
H	-1.468615	-1.530564	-1.412232	0.173024	-0.014143
Na	2.934019	-0.004036	-0.172161	0.950447	0.945545

BMe_3OSiMe_2HNa

Electronic energy: -751.989400

Electronic energy + zero-point energy: -751.785853

Electronic energy + thermal energy correction: -751.763910

Electronic energy + thermal enthalpy correction: -751.762728

Electronic energy + thermal free energy correction: -751.841664

Electronic energy of $OSiMe_2HNa$ fragment in BMe_3OSiMe_2HNa basis set: -607.380430

Electronic energy of BMe_3 fragment in BMe_3OSiMe_2HNa basis set: -144.487410

Electronic energy of $OSiMe_2HNa$ fragment in $OSiMe_2HNa$ basis set: -607.378054

Electronic energy of BMe_3 fragment in BMe_3 basis set: -144.486751

Table S69. Cartesian coordinates, Mulliken and APT charges of all atoms at **BMe_3OSiMe_2HNa**.

Symbol	X	Y	Z	Mulliken	APT
C	2.591906	-1.421754	0.152997	-0.809617	-0.405355
Si	1.509142	0.109756	0.338503	0.554158	1.857335
H	2.213344	-2.263006	0.743583	0.173781	-0.009758
H	3.614381	-1.219343	0.488536	0.188988	0.008391
H	2.644446	-1.732889	-0.896503	0.180213	-0.000374
H	1.497137	0.497039	1.779126	-0.047537	-0.397212
O	-0.001380	-0.297917	-0.175033	-0.723156	-1.487317
C	2.283966	1.502637	-0.654021	-0.736318	-0.430542
H	3.340068	1.617484	-0.385728	0.182432	0.001903
H	1.779447	2.455405	-0.476127	0.201549	0.029473
H	2.227496	1.283524	-1.725746	0.188764	0.009154
B	-1.326086	0.489745	0.081053	1.078953	1.121280

C	-2.301875	0.226436	-1.220785	-0.728010	-0.204925
C	-1.985561	-0.092707	1.470315	-0.808911	-0.206340
C	-1.029980	2.087138	0.213079	-0.987028	-0.233179
H	-0.618125	2.513654	-0.712658	0.145866	-0.056460
H	-0.329718	2.332110	1.026156	0.140897	-0.056515
H	-1.957881	2.633587	0.429148	0.123167	-0.038804
H	-2.705319	-0.793541	-1.376570	0.099207	-0.131061
H	-1.800280	0.508321	-2.156866	0.163221	-0.049692
H	-3.199306	0.853174	-1.138831	0.158149	-0.032295
H	-2.255777	-1.166419	1.471223	0.096963	-0.127173
H	-2.920577	0.431713	1.708487	0.146237	-0.039859
H	-1.310844	0.050676	2.325961	0.156954	-0.048900
Na	-1.095977	-2.128543	-0.419703	0.861076	0.928225

$NaHBMe_3$

Electronic energy: -307.393616

Electronic energy + zero-point energy: -307.269298

Electronic energy + thermal energy correction: -307.257551

Electronic energy + thermal enthalpy correction: -307.256370

Electronic energy + thermal free energy correction: -307.310854

Electronic energy of NaH fragment in $NaHBMe_3$ basis set: -162.801139

Electronic energy of BMe_3 fragment in $NaHBMe_3$ basis set: -144.484965

Electronic energy of NaH fragment in NaH basis set: -162.800168

Electronic energy of BMe_3 fragment in BMe_3 basis set: -144.484655

Table S70. Cartesian coordinates, Mulliken and APT charges of all atoms at **$NaHBMe_3$**.

Symbol	X	Y	Z	Mulliken	APT
Na	-1.913548	-0.000179	-0.481753	0.668252	0.787259
H	2.582000	-0.000202	0.481034	0.132040	-0.048813
H	0.560497	-1.393140	1.541381	0.163687	-0.024803
C	2.038621	-0.000267	-0.475733	-0.749740	-0.160555
H	0.560358	1.393924	1.541108	0.163666	-0.024848
H	2.376944	-0.883129	-1.033913	0.152491	-0.049389
C	-0.007371	-1.366351	0.601846	-0.632129	-0.148150

H	2.377453	0.882143	-1.034316	0.152507	-0.049379
B	0.437309	0.000127	-0.233251	0.157416	0.629738
C	-0.007211	1.366775	0.601399	-0.632033	-0.148041
H	0.244272	-2.284458	0.055694	0.170748	-0.041502
H	-1.060788	-1.498927	0.937766	0.057756	-0.139631
H	0.244463	2.284772	0.055071	0.170749	-0.041510
H	-1.060748	1.499364	0.936950	0.057751	-0.139652
H	-0.106204	0.000050	-1.380308	-0.033161	-0.400724

$NaHBMe_3$ regeneration – transition state

Electronic energy: -914.786832

Electronic energy + zero-point energy: -914.580158

Electronic energy + thermal energy correction: -914.553619

Electronic energy + thermal enthalpy correction: -914.552437

Electronic energy + thermal free energy correction: -914.645780

Electronic energy of $OSiMe_2HNa$ fragment in transition state basis set: -607.384413

Electronic energy of BMe_3 fragment in transition state basis set: -144.528697

Electronic energy of NaH fragment in transition state basis set: -162.802733

Electronic energy of $OSiMe_2HNa$ fragment in $OSiMe_2HNa$ basis set: -607.383269

Electronic energy of BMe_3 fragment in BMe_3 basis set: -144.528263

Electronic energy of NaH fragment in NaH basis set: -162.801584

Table S71. Cartesian coordinates, Mulliken and APT charges of all atoms at **transition state of $NaHBMe_3$ regeneration.**

Symbol	X	Y	Z	Mulliken	APT
C	-3.824488	-0.070918	0.785439	-0.850219	-0.412390
Si	-2.090016	-0.612855	0.243479	0.539596	1.859221
H	-3.777856	0.732596	1.528826	0.166564	-0.015365
H	-4.384219	-0.903329	1.227208	0.174997	-0.016801
H	-4.396254	0.299315	-0.073688	0.161981	-0.014655
H	-1.456445	-1.216167	1.475577	-0.067635	-0.459345

O	-1.200210	0.577032	-0.364369	-0.852596	-1.439517
C	-2.340890	-2.063385	-0.950235	-0.766091	-0.413085
H	-2.919296	-2.867028	-0.479694	0.183155	-0.018761
H	-1.386725	-2.485835	-1.283177	0.153886	-0.009381
H	-2.885073	-1.733297	-1.842692	0.181832	-0.011885
B	1.553862	0.379794	-0.012684	0.916289	1.052477
C	1.704047	1.214909	-1.352623	-0.841517	-0.331683
C	1.328833	1.032201	1.412340	-0.813225	-0.306812
C	1.603930	-1.204402	-0.105322	-0.792838	-0.341412
H	0.574842	-1.528269	-0.306893	0.256196	0.039301
H	1.890697	-1.699656	0.831393	0.184480	-0.026581
H	2.202828	-1.584739	-0.942458	0.168853	-0.038323
H	1.854672	2.293229	-1.206269	0.144280	-0.043867
H	0.814734	1.043337	-1.974494	0.216711	0.024949
H	2.566720	0.866419	-1.928104	0.151457	0.006036
H	0.432551	0.607286	1.879679	0.183299	0.013716
H	1.256962	2.127948	1.424465	0.111446	-0.061615
H	2.182523	0.784887	2.053398	0.146439	0.004478
Na	-0.536273	2.541380	-0.306294	0.847692	0.926056
Na	4.466461	-1.338319	0.319654	0.849997	0.828883
H	4.461275	0.643945	0.302031	-0.755028	-0.793637
